# Reversing Lanmodulin's Metal‐Binding Sequence in Short Peptides Surprisingly Increases the Lanthanide Affinity

**DOI:** 10.1002/anie.202510453

**Published:** 2025-09-23

**Authors:** Sophie M. Gutenthaler‐Tietze, Jerome Kretzschmar, Satoru Tsushima, Robin Steudtner, Björn Drobot, Lena J. Daumann

**Affiliations:** ^1^ Department of Chemistry Ludwig‐Maximilians‐Universität München Butenandtstraße 5–13 81377 München Germany; ^2^ Institute of Ressource Ecology Helmholtz‐Zentrum Dresden‐Rossendorf e.V Bautzner Landstraße 400 01328 Dresden Germany; ^3^ Chair of Bioinorganic Chemistry Heinrich‐Heine‐Universität Düsseldorf Universitätsstraße 1 40225 Düsseldorf Germany; ^4^ Institute of Integrated Research Institute of Science Tokyo Tokyo 152–8550 Japan

**Keywords:** CD, EF‐hand peptides, Fluorescence, ITC, Lanmodulin, Lanthanides, NMR

## Abstract

Rare earth elements (REEs) are essential for a clean energy future, high tech, and medicine. For these applications the chemically similar elements need to be tediously separated. Recent discoveries that specific lanthanide‐binding proteins such as lanmodulin (LanM) exist in nature have prompted the development of bio‐inspired separation methods for REEs. Peptides hold great potential for tuning binding sites in various applications as they are easily synthesised, modifiable, and can be immobilised. Here we use the EF‐hand binding site sequences of LanM, the naturally lanthanide‐binding EF‐hand protein from *Methylorubrum extorquens* AM1, as a blueprint for peptides with potential applications in REE recycling. We show with time‐resolved laser‐induced fluorescence spectroscopy (TRLFS), isothermal titration calorimetry (ITC), and nuclear magnetic resonance (NMR) spectroscopy in combination with molecular dynamics (MD) simulations and circular dichroism (CD) spectroscopy the surprising result that reversing the natural sequence of LanM's metal‐binding loops leads to an increased binding affinity of about one order of magnitude for three out of four natural sequences. Furthermore, we were able to identify structural features responsible for the affinity boost and were able to obtain – only by exchanging one amino acid – a linear uncapped 12‐amino‐acid peptide with a 150 nM affinity for lanthanides.

## Graphical abstract

Accidentally reversing the lanthanide‐binding sequences of lanmodulin‐inspired short peptides lead to the serendipitous discovery of a set of peptides with a one order of magnitude higher lanthanide‐binding affinity than the uncapped peptides synthesised in the correct direction.

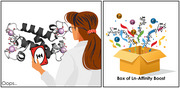

## Introduction

Metal‐binding peptides based on calcium‐binding EF‐hand proteins such as calmodulin have long been under investigation for their lanthanide(Ln)‐binding capabilities^[^ ^1^ ^]^ as it has been shown in the literature that calmodulin and related proteins are capable of binding Lns with higher affinity than the target metal calcium.^[^ ^2^, ^3^, ^4^, ^5^ ^]^

Those bio‐inspired peptides were a great starting point for the design of potent Ln‐chelators for a wide range of applications, including bio probes known as lanthanide‐binding tags (LBT),^[^ ^6^, ^7^ ^]^ possible medical applications,^[^ ^8^ ^]^ and the separation of Lns and actinides^[^ ^9^ ^]^ However, with the discovery of Ln‐dependent bacteria^[^ ^10^, ^11^, ^12^ ^]^ also natural proteins specific for Ln‐binding, such as a variety of lanmodulins^[^ ^13^, ^14^, ^15^ ^]^ (LanM) and lanpepsy,^[^ ^16^ ^]^ have been discovered. These proteins, designed by nature to bind Lns with a high affinity and selectivity, present a unique starting point for optimising artificial Ln‐binding peptides. Recently, we have investigated the four EF‐hand binding loop sequences as uncapped 12‐amino‐acid peptides and their affinities for the lanthanides Tb(III) and Eu(III) as well as the actinide Cm(III) detached from the protein scaffold.^[^ ^17^ ^]^ For those four peptides we found lower micromolar affinity (on average around 5 µM) for Eu(III) and Tb(III) and a slightly higher affinity for the actinide Cm(III) as well as no observable binding to Ca(II) under the tested conditions. We concluded that the *f*‐element (lanthanide/actinide) specificity of the full protein LanM seems to be preserved in the peptide sequences, but the reported pM affinity for the high‐affinity sites of LanM was lost due to the high flexibility of the short peptides and the absence of cooperativity and the necessary preorganisation by the protein scaffold.^[^ ^14^ ^]^ Nevertheless, the potential of these peptide sequences for rare earth element (REE)‐recycling has been recently shown in different proof‐of‐concept experiments. For example, Renner and co‐workers immobilised a slightly prolonged (16 amino acids) and modified (acetylated N‐terminus and amidated C‐terminus) LanM EF1‐binding loop peptide on a gold sensor for selectively binding REEs^[^ ^18^ ^]^ as well as showed the successful peptide‐functionalisation of membrane fibres for REE recovery.^[^ ^19^ ^]^ Sree et al. used immobilised LanM EF1 as 12‐aminoacid peptide (free N‐terminus) for the separation of Eu(III) from K(I), Mg(II), Ca(II), Al(III) and some transition metals,^[^ ^20^ ^]^ and Cao and co‐workers successfully tested the LanM EF2‐binding loop peptide as well as two modified versions (prolonged or acetylated N‐terminus/ amidated C‐terminus) for the selective recovery of Sc(III).^[^ ^21^ ^]^ In addition, the possible application of LanM‐derived peptides in biosensors for cerium detection has been shown recently.^[^ ^22^ ^]^

Peptides, as tuneable ligands, offer significant potential for bio‐based selective adsorption due to their versatility in immobilisation. Optimising these bio‐inspired peptide sequences is crucial for enhancing the efficiency of peptide‐based recycling methods for Lns and the separation of Lns and actinides. Therefore, the further optimisation of such bio‐inspired peptide sequences is of high interest. Herein, we present the serendipitous finding that reversing the 12‐amino‐acid peptide sequences of some LanM metal‐binding loops yield higher affinity metal chelators than uncapped peptides based on native sequences.

## Results and Discussion

**Figure 1:**
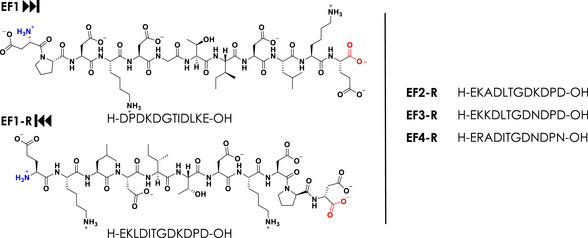
Comparison of the structures of the native sequence EF1 (= ”forward”) and the reversely‐linked peptide EF1‐R on the left and the sequences of the reverse peptides EF2‐R, EF3‐R, and EF4‐R on the right; Free N‐terminus highlighted blue and free C‐terminus in red.

Starting our investigation into the metal‐binding properties of the EF‐hand loop peptides^[^ ^17^ ^]^ of LanM, first isolated from *Methylorubrum extorquens* AM1, as uncapped peptides we accidentally synthesised one sequence the wrong way by fully reversing it. In solid‐phase peptide synthesis (SPPS) peptides are assembled from C‐ to N‐terminus. This contrasts with natural protein biosynthesis, which proceeds from N‐ to C‐terminus and defines the reading direction of proteins/ peptides. Thus, by accidently reading the sequence as C‐ to N‐terminus we ended up with a completely reversed peptide. Curious whether this “reverse” EF‐hand (EF‐R, R  =  reverse sequence) would still bind Lns, we were surprised by the excellent affinity this chelator presented. Researchers often use scrambled amino acid sequences as negative controls for metal‐binding to show that not only the number of carboxylate side chains is relevant, but the sequence as well.^[^ ^18^, ^23^ ^]^ Here, we investigate all four EF‐hand peptides of LanM synthesised in reversed order (EF1‐R, EF2‐R, EF3‐R, EF4‐R, free amine at N‐termini, and free carboxylate at C‐termini) and compare them to the previously reported four EF‐hands with the native (= “forward”, also with free amine at N‐termini and free carboxylate at C‐termini) sequences (Figure 1). A beneficial side effect is that the R‐sequences are less prone to aspartimide formation as well as other side reactions during the solid phase peptide synthesis and are thus easier to synthesise with less side products in a higher yield.^[^ ^24^, ^25^, ^26^ ^]^

### Initial Binding Studies on the Reverse Peptides

To track Ln‐binding to the peptides we used the luminescent properties of Eu(III) in time‐resolved laser‐induced fluorescence spectroscopy (TRLFS). The luminescence lifetimes of the Eu(III)‐aquo ion and peptide‐Eu(III)‐complexes are distinct due to the different number of Eu(III)‐coordinating water molecules and thus different extent of quenching.^[^ ^27^ ^]^ In addition, the shape of the emission spectra allows for some distinction of the Eu‐species in different environments (ligand field).^[^ ^28^, ^29^ ^]^ To retrieve the metal‐binding information, we have previously successfully used parallel factor analysis (PARAFAC) for TRLFS data deconvolution. For the forward peptides we showed with the combination of isothermal titration calorimetry (ITC) and TRLFS experiments that the obtained data could be best explained with more than one EF:Eu(III) species.^[^ ^17^ ^]^ In order to increase the observability of potential EF:Eu(III) complexes of higher stoichiometry, we employed a modified setup for the reverse peptides that enabled us to probe two regimes simultaneously: Excess of peptide and excess of Eu(III) (Figure S3). The selected chemical system provides the opportunity to thermodynamically investigate the following reactions:

$$\mathrm{EF}+{\mathrm{Eu}}^{\mathrm{III}}\;\to \;1:1\;\mathrm{EF}:{\mathrm{Eu}}^{\mathrm{III}}\;\;\;K_{D1:1}$$

$$\;1:1\;\mathrm{EF}:{\mathrm{Eu}}^{\mathrm{III}}+{\mathrm{Eu}}^{\mathrm{III}}\;\to \;1:2\;\mathrm{EF}:{\mathrm{Eu}}^{\mathrm{III}}\;\;\;K_{D1:2}$$

**Figure 2:**
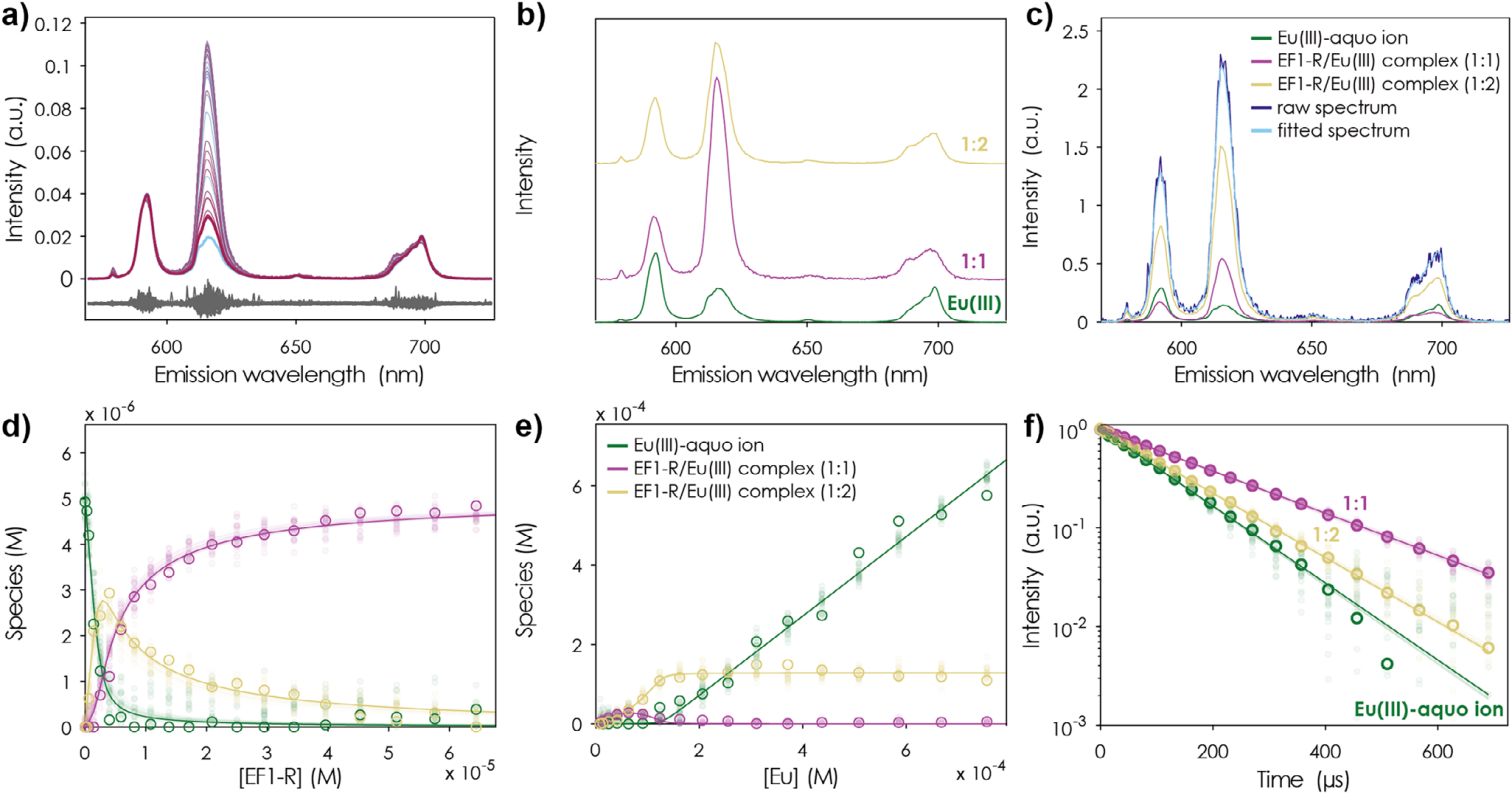
Combined EF1‐R to Eu(III) and Eu(III) to EF1‐R TRLFS titration experiment (10 mM MOPSO, pH 6.6, 100 mM KCl), *λ*_ex_ (Eu) = 394 nm. a) t_0_ spectra of the PARAFAC model (blue to red for increasing peptide/Eu(III) complex concentration) normalised to the ^5^D_0_→ ^7^F_1_ transition; residues shown in grey. b) Stacked spectra of the three different species: Eu(III)‐aquo ion, the 1:1 EF1‐R to Eu(III) complex and the 1:2 EF1‐R to Eu(III) complex. c) Deconvoluted spectra of a representative step showing the spectrum of the Eu(III)‐aquo ion, the 1:1 EF1‐R to Eu(III) complex, and the 1:2 EF1‐R to Eu(III) complex. d) Speciation of the first part of the titration experiment with increasing peptide concentration. e) Speciation of the second part of the titration experiment with increasing Eu(III) concentration. f) Lifetimes of the observed species. The colour code shown in E applies for B, D, and F. Overlaid lines/symbols represent Monte Carlo (MC runs used for error estimation (see Supporting Information Section 3.3).

With excess peptide we find that a 1:1 complex dominates for all reverse peptides (Figures 2d and S21–S23D) while with excess Eu(III) the equilibrium shifts to the 1:2 EF:Eu(III) complex (Figures 2e and S21–S23E). The presence of 1:2 EF:Eu(III) complexes has also been previously demonstrated for the uncapped forward EF‐hand sequences.^[^ ^17^ ^]^ To validate the new setup, the forward peptide EF1 was used as control and analysed analogously to show the robustness of the new titration setup and analysis workflow (Figure S20 and Supporting Information Section 2.3 for a detailed description of the used workflow). Interestingly, the experiment suggests that the formation of a 1:2 EF:Eu(III) complex plays a larger role in the speciations for the reverse peptides than for the original sequence EF1, i.e., the forward peptides. The formation of a 1:2 EF:Ln(III) complex is corroborated by NMR titration experiments with La(III), Eu(III), and Lu(III) (La(III) and Lu(III) were used due to their diamagnetism and Eu(III) in order to link the NMR data to the TRLFS), indicating the predominance of unique EF:Ln(III) species at higher Ln(III) concentrations. Thereby, NMR spectra display speciation changes upon formation of the 1:1 and 1:2 EF:Ln(III) complexes. Especially the signals due to Gly, Pro, and Lys are useful probes in that context (*c.f*. Figures S36–S42). For instance, the signals of the AB spin system of Gly's diasterotopic Hα show a bimodal titration behaviour: Over the first few titration steps the metal‐free peptide's distinct signals successively shift towards one another and merge until as of higher EF:Ln(III) ratios the signals diverge again. By contrast, adding La(III) to a calmodulin‐derived 13‐amino acid peptide is reported to simply show unimodal titration behaviour, just continuously increasing Gly's Hα signals’ distance approaching asymptotic saturation for excess La(III) upon formation of a 1:1 complex.^[^ ^30^ ^]^ We thus conclude that Gly's signals signature – bimodal versus unimodal – is indicative of forming a 1:2 complex beyond the 1:1 complex, as observed for both EF1 and EF1‐R with all three tested Lns. Depending on the Ln, some minor variations in terms of distinctiveness, magnitude of shifting and broadening as well as the ratio at which the second complex emerges are observed. The overall spectral changes due to the formation of a 1:2 complex were notably well observable in the Lu(III) to EF1‐R titration series, allowing ^1^H signal assignment of a 1:2 complex, as demonstrated for EF1‐R‐Lu(III) in the Supporting Information (Table S14 and Figures S43–S46). Observations of EF:Ln(III) 1:2 complexes are also supported by MD simulations of an EF1‐R:Eu(III) 1:2 complex, featuring binding motifs and structural characteristics which are in line with the NMR results (Figure S82).

The obtained affinity (*K*_D_) values and luminescence lifetimes for all four reverse peptides with Eu(III) are shown in Table 1. Spectra, luminescence lifetimes, and correspondingly derived species distributions are shown for EF1‐R in Figure 2 and provided as Supporting Information for EF2‐R, EF3‐R, and EF4‐R (Figures S21–S23). We find similar binding affinities and luminescence lifetimes for the 1:1 complex with Eu(III) for the peptides EF1‐R, EF2‐R, and EF3‐R, all of which being around one order of magnitude higher in affinity as the forward peptides (EF1: 4.4 ± 0.3 µM (this work), EF2: 4.4 ± 0.8 µM,^[^ ^17^ ^]^ EF3: 3.1 ± 2.8 µM^[^ ^17^ ^]^) while EF4‐R is markedly different with a very similar affinity as the corresponding forward peptide EF4 (2.4 ± 1.9 µM^[^ ^17^ ^]^).

**Table 1:** *K*_D_ values determined by TRLFS and ITC (pH 6.6, 10 mM MOPSO, 100 mM KCl) as well as the by TRLFS obtained lifetimes (*τ*) of the observed complexes; the lifetimes of the Eu(III)‐aquo ions are shown in Table S9. All errors were obtained by a MC approach.

	*K*_D_ value by ITC (µM)		*K*_D_ values (µM) and lifetimes (µs) by TRLFS			
EF‐Eu(III)	1:1	1:2	1:1	*τ*	1:2	*τ*
EF1‐R	0.52 ± 0.03	10.9 ± 0.6	0.50 ± 0.11	204 ± 3	1.1 ± 0.3	134 ± 2
EF2‐R	0.66 ± 0.03	13.6 ± 0.7	0.59 ± 0.15	199 ± 2	2.9 ± 1.1	131 ± 4
EF3‐R	0.30 ± 0.01	9.3 ± 0.4	0.32 ± 0.09	202 ± 2	1.6 ± 0.6	134 ± 2
EF4‐R	8.8 ± 0.5	87 ± 14	2.5 ± 0.5	201 ± 2	10.8 ± 4.1	131 ± 2

We corroborated these findings with isothermal titration calorimetry (ITC) under similar conditions using a setup in which each replicate was measured with a different peptide concentration in the cell, but the same Eu(III) concentration in the syringe. This enabled a global analysis of the binding by employing a global fit over all three replicates, again supporting the observability of both complexes (Supporting Information Section 2.2). With this we were able to observe the same affinity trend (Table 1): An affinity increased by about one order of magnitude for EF1‐R, EF2‐R, and EF3‐R in comparison to the forward sequences (EF1: 8.6 ± 0.2 µM, EF2: 8.6 ± 0.1 µM, and EF3: 8.0 ± 1.2 µM)^[^ ^17^ ^]^ and a very similar affinity of EF4‐R when compared to EF4 (8.9 ± 0.2 µM^[^ ^17^ ^]^). It is evident that both methods, TRLFS and ITC, reveal the same affinity trend, which results especially for the 1:1 complex in a very consistent picture. While a similar trend can also be observed for the 1:2 complex, the differences between the methods are more pronounced. We explain this with the fundamentally different underlying mechanisms of the two techniques: In TRLFS, the luminescence of the Eu(III) ion is used to directly probe the coordination sphere of the metal. In contrast, ITC evaluates the measured heat signal, which represents a superposition of all contributing processes, such as stripping of hydration shells, carboxylate binding, etc. Integration of the thermogram results in a rather featureless, sigmoidal curve. We believe that this curve complicates the clear characterization of the 1:2 complex by ITC, even though we applied a global analysis across three different concentration sets and thus leads to greater deviations.

For the reverse peptides, ITC again shows that the formation of the EF‐Eu(III) complexes is overall exergonic, but endothermic, thus, though thermodynamically favoured, mainly driven by entropic effects.^[^ ^17^ ^]^ This is also in accordance with observations for other EF‐hand protein‐based peptides in the literature.^[^ ^18^, ^23^ ^]^ Interestingly, the entropy contribution is larger for EF1‐R through EF3‐R in comparison to the previously investigated forward peptides.^[^ ^17^ ^]^ Additionally, we also tested all reverse peptides for their Ca(II)‐binding by ITC and CD. For the ITC we used a setup which used ten times the amount of CaCl_2_ in comparison to EuCl_3_ in the syringe. With this, we were not able to observe any complex formation with Ca(II) as the obtained data hardly differs from the control measurement (Figure S19). Thus, from these experiments we can exclude an affinity in the lower millimolar range as observed in the literature for other uncapped dodecapeptides.^[^ ^31^, ^32^ ^]^ By CD spectroscopy we were able to observe for all reverse peptides (see Figure S31) structural changes when adding a greater excess of CaCl_2_ suggesting a Ca(II)‐affinity in the middle to higher millimolar range. Interestingly, while a 500‐times excess of Ca(II) already leads to a saturation of all reverse peptides, a 1000‐times excess of Ca(II) does not introduce any structural change in the forward peptide EF1 which perfectly fits the data previously reported.^[^ ^17^ ^]^ So while the affinity of some of the reverse peptides is in comparison to the forward peptides increased for Lns, the affinity for Ca(II) increased as well (albeit still far below the Ln‐binding affinity).

To understand the one order of magnitude higher Ln‐affinity of the reverse peptides compared to the forward peptides when looking at the 1:1 complex, as well as the outlier EF4‐R, we examined the role of different amino acids and functional groups in the sequences: (1) The role of the terminal carboxylate, (2) effect of the proline on metal‐binding, and (3) the C‐terminal amino acid (N versus D) in the EF4 and EF4‐R sequences.

### Influence of the C‐Terminal Carboxylate

**Figure 3:**
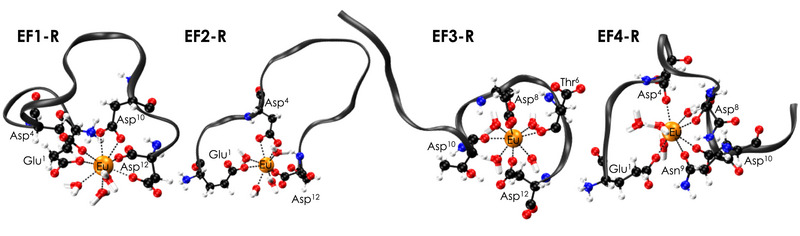
Representative ball‐and‐stick drawings of Eu(III)‐bound reversed peptides EF1‐R, EF2‐R, EF3‐R, and EF4‐R from MD trajectory. The grey ribbon depicts the peptide backbone; nitrogen atoms are shown in blue, carbon atoms in black, oxygen atoms in red, hydrogen atoms in white, and Eu(III) in orange.

MD simulations hinted that the C‐terminal carboxylate is likely one of the reasons for the different magnitude in affinity for some of the forward versus the reverse peptides. For EF1‐R and EF2‐R (Figures 3 and S79) the MD simulations suggested not only monodentate binding through the carboxylate sidechain of the C‐terminal Asp, but also the C‐terminal carboxylate itself. This is in contrast to the forward peptides in which no hint for the involvement of the C‐terminal carboxylate in addition to the Glu sidechain carboxylate was observed.^[^ ^17^ ^]^

To experimentally investigate this hypothesis, NMR titrations of EF1 and EF1‐R were performed with diamagnetic La(III) and Lu(III), as well as with paramagnetic Eu(III). For reference, i.e., in the absence of lanthanides, for both peptides, all ^1^H and ^13^C signals were assigned by using a combination of ^1^H NMR spectroscopy and 2D NMR experiments (TOCSY, HSQC, HMBC). The full assignment can be found in the Supporting Information (EF1: Figure S33 and Tables S10, S11, EF1‐R: Figure S35 and Tables S12, S13). Especially challenging was the unambiguous assignment of the four aspartates and the two lysines due to severe signal overlapping. Complete ^13^C signal assignment of the metal‐free peptides (EF1 and EF1‐R) was only feasible for peptide concentrations twice as high as those used for the titration samples. For better detection of signals (Hα) and correlations (Hα–C) in proximity of the H_2_O resonance, samples were lyophilised and redissolved in the same volume of D_2_O, maintaining sample composition (concentration of peptide and electrolyte) and pH range (buffered). The metal‐free peptide, unsurprisingly, exhibits chemical shifts resembling those reported for a random‐coil conformation.^[^ ^33^ ^]^ For both EF1 and EF1‐R, compared to the other amino acids in the respective peptide, we observe the most upfield carbonyl ^13^C chemical shift for the N‐terminal amino acid as well as the most upfield NH ^1^H chemical shift for the C‐terminal amino acid, rendering them a good starting point for sequential assignment.

**Figure 4:**
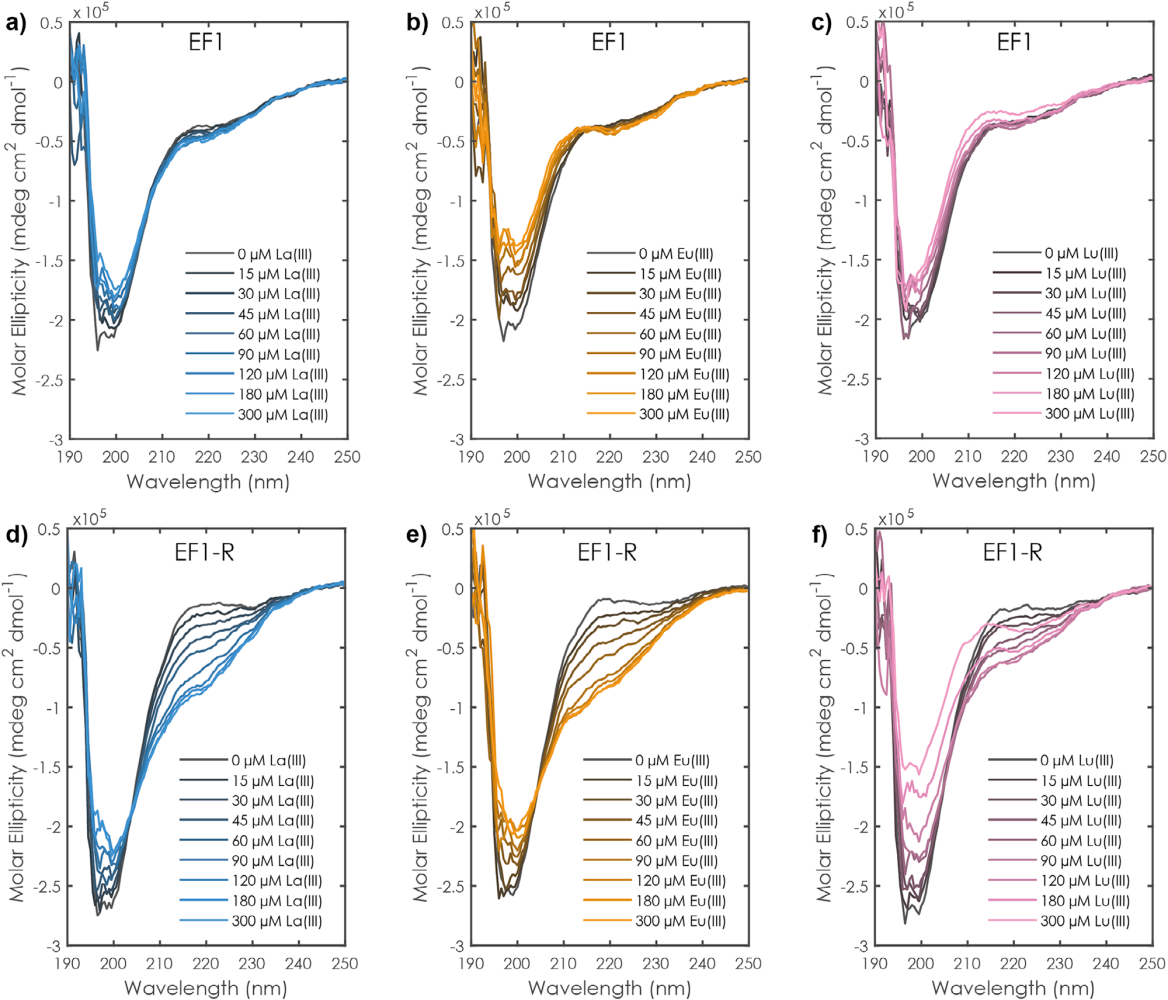
CD spectra of lanthanide chloride to peptide (60 µM) titration experiments at 25 °C and pH 6.6 (10 mM MOPSO, 100 mM KCl) a) La(III) to EF1, b) Eu(III) to EF1, and c) Lu(III) to EF1, d) La(III) to EF1‐R, e) Eu(III) to EF1‐R, and f) Lu(III) to EF1‐R.

Upon titrating La(III), Eu(III), or Lu(III) to the two examined peptides, especially the NH associated signals exhibit significant shifts and signal broadening particularly for the densely charged Lu(III) and paramagnetic Eu(III), thus indicating a structural change in the peptide backbone (Figures S36–S42). In this regard, one has to keep in mind two general things: the signals represent molar fraction‐weighted averages, and the (non‐paramagnetic) broadening is most pronounced for sample compositions where several species (metal‐free peptide, 1:1 and 1:2 complexes) coexist in dynamic equilibrium. The structural change upon Ln‐addition is supported by (circular dichroism) CD spectroscopy (Figures 4 and S30), indicating significantly more pronounced changes for EF1‐R than for EF1, mirroring the larger susceptibility of the reverse peptide to form a 1:2 EF:Ln(III) complex, especially with the small lanthanide Lu(III) (Figure 4f). Spectra obtained for the metal‐free reverse peptides fit the stereotypical random‐coil formation while the forward peptides already show a slight band at 222 nm indicating more secondary structure elements (Figure 4 and literature^[^ ^17^ ^]^). This may hint toward a generally better preorganisation in the forward peptide sequence.

**Figure 5:**
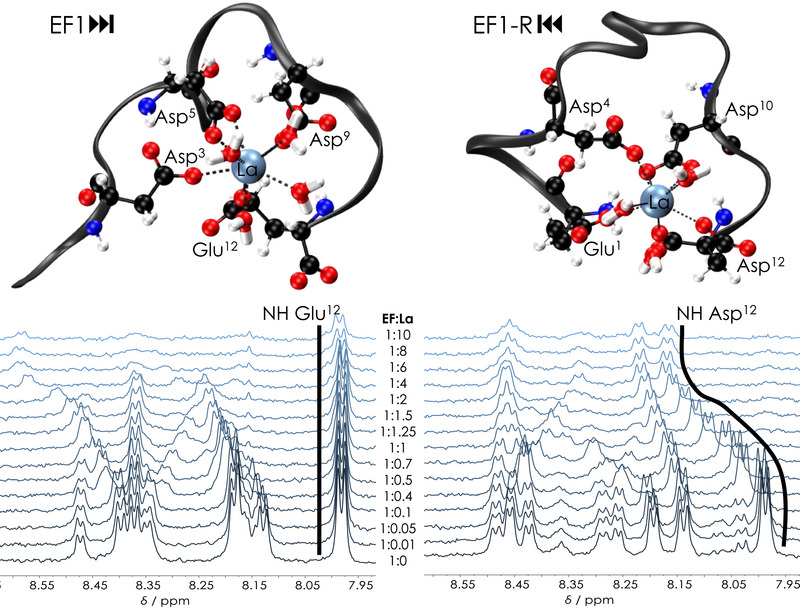
Top: Representative ball‐and‐stick drawings for La(III)‐bound EF1 and EF1‐R from MD trajectory, see Figure S79 for superimposed snapshots and Figure S80 for a comparison with the Lu(III) MD simulations. The grey ribbon depicts the peptide backbone; light blue: lanthanum, blue: nitrogen, black: carbon, red: oxygen, and white: hydrogen. Bottom: NH region of ^1^H NMR spectra of La(III) to peptide titrations for EF1 (left) and EF1‐R (right) titrations (600 MHz, H_2_O/D_2_O (9:1) + 0.003% TMSP‐*d_4_*, pH 6.6, 30 mM MES‐*d_13_*, 100 mM KCl).

To get further insights on the coordination by the C‐terminal carboxylate by MD simulations suggested, the NMR titrations of EF1 and EF1‐R were closely compared. Figure 5 depicts the NH NMR spectral region of both the EF1 and EF1R titrations series with LaCl_3_.

Upon La(III) addition, the C‐terminal amino acid's (Glu12) NH signal remains unaffected for EF1 while in the case of EF1‐R a significant downfield shift of the Asp12 NH can be observed, approaching asymptotic saturation at higher La(III) concentrations. These findings support the results of the MD simulations pinpointing that in the reverse peptides not only the C‐terminal aspartate's side chain carboxylate is involved in metal binding, but also the C‐terminal carboxylate itself (Table S15 and S16).

This hypothesis is further supported experimentally by the investigation of two modified peptides, termed EF1‐OMe and EF1‐R‐OMe, where the metal‐binding capability of the terminal carboxylate has been blocked by the introduction of a methyl ester group. For both peptides a decrease in overall structural change can be observed in a Eu(III) to peptide titration CD experiment (Figure S30E,F) when compared to the unmodified peptides (Figure 4b,e). However, the effect is significantly more pronounced for EF1‐R‐OMe. For free EF1 and EF1‐OMe very similar CD spectra are obtained with a weak band at 222 nm and the pronounced random‐coil feature at 200 nm. It is not surprising that EF1‐OMe behaves differently in comparison to EF1 as the incorporation of a methyl ester changes the overall charge state of the peptide and therefore presumably changes the secondary coordination sphere as well as intramolecular interactions such as hydrogen bonding. Both, ITC and TRLFS data sets of the modified peptides are best modelled with the sole formation of a 1:1 complex and both reveal a reduced affinity for Eu(III) (Tables S7, S8; Figures S15–S18, and S25–S26). The overall reduction of the affinity, not only for EF1‐R‐OMe, but also for EF1‐OMe toward Eu(III), might likely be caused by the change in the peptide's net charge, i.e., being less negative upon esterification of the C‐terminal carboxylate. For EF1‐R‐OMe, the Eu(III)‐ binding affinity determined by ITC is with 31.8 ± 2.1 µM for EF1‐R‐OMe versus 520 ± 30 nM for EF1‐R significantly more reduced than for EF1‐OMe (17 ± 1.2 µM versus EF1: 8.55 ± 0.17 µM^[^ ^17^ ^]^). TRLFS experiments depict the same trend (EF1‐OMe: 9.5 ± 0.5 µM versus EF1: 4.4 ± 0.3 µM, EF1‐R‐OMe: 8.6 ± 0.9 µM versus EF1‐R: 500 ± 110 µM) in the respective same binding range. From these results we concluded that indeed the involvement of the C‐terminal carboxylate is of special importance for EF1‐R and EF2‐R whereas for the forward peptides it does not. One key difference at the C‐terminus between the forward and the reverse peptides is an Asp instead of a Glu residue. This results in a binding motif in which two carboxylates are separated by a two‐carbon (ethylene) spacer being comparable to a C3‐substituted succinate. For Glu it would be a three‐carbon spacer, making the binding motif more flexible and comparable to a C4‐substituted glutarate. In both cases the Ln‐binding to two carboxylates simultaneously would be entropically very favourable. However, the higher flexibility of the Glu side chain seems to prevent the binding to both carboxylates, while the shorter less flexible side chain of Asp might just be the ideal fit to achieve a more stable Ln‐complexation. When comparing the Eu(III) affinity of succinic and glutaric acid one notices only a slightly higher affinity of succinic acid (log β (succinic acid) = 2.99 ± 0.01, log β(glutaric acid) = 2.66 ± 0.01).^[^ ^34^ ^]^ The succinate‐like binding motif bears resemblance to the binding‐motif obtained when the synthetic amino acid γ‐carboxyglutamic acid (Gla) is incorporated into peptides. Gla's side chain contains two carboxylates with a one‐carbon space (malonate‐like) and has been used in the design of coiled‐coils. While sequences with Gla residues decrease the stability in the absence of metal, the complex stability is significantly increased. This is explained by electrostatic repulsion due to the close proximity of the carboxylates.^[^ ^35^ ^]^

While the N‐terminal‐capping (via acetylation) and C‐terminal‐capping (via amidation) of peptides is often used in EF‐hand peptide studies to increase the peptide stability and to mimic the native protein without introducing unnatural terminal charges,^[^ ^23^ ^]^ our findings demonstrate that it might be worth considering the combination of a C‐terminal Asp and an uncapped C‐terminus as potent Ln‐binding motif which can significantly enhance the Ln‐affinity in short peptides.

### Influence of the Structure‐determining Amino Acid Proline

For LanM it had been hypothesised that the proline in the sequence is one reason for the protein's high selectivity toward Lns.^[^ ^13^ ^]^ Proline in position 2 in a EF‐hand binding loop is indeed very uncommon in the related calmodulin EF‐hand binding proteins in which in almost 30% of cases the second position is occupied by Lys, followed by Ala, Gln, and Thr. Proline residues are more frequently observed in other positions (e.g., position 11).^[^ ^36^, ^37^ ^]^ The so far obtained literature corpus on lanmodulins shows a prevalence for prolines in position 2 (lanmodulin‐homologue in *Beijerinckiaceae bacterium* RH AL1^[^ ^15^ ^]^: 3 out of 4 prolines in position 2; *Methylobacterium aquaticum* 22A^[^ ^38^ ^]^: 2 out of 4 prolines in position 2, *Hansschlegelia quercus* LanM^[^ ^14^ ^]^: 1 out of 4 prolines in position 2) followed by lysine and alanine (correlating with the sequences of calmodulins), supporting the claim for its potential role for Ln‐selectivity. Furthermore, it has already been shown that replacing proline with alanine in the protein sequence of lanmodulin (*M. extorquens* AM1) significantly reduces Ln‐selectivity over calcium while maintaining the observed Ln‐affinity.^[^ ^13^ ^]^ Since this cyclic amino acid is known to influence the shape of the backbone more than others, it is important to also closely examine the influence of proline in the three high‐affinity peptides (EF1‐R, EF2‐R, and EF3‐R) in comparison to the forward peptides. This is especially interesting as the reverse peptides strongly deviate from the typical EF‐hand amino acid sequence.^[^ ^36^, ^37^ ^]^

**Figure 6:**
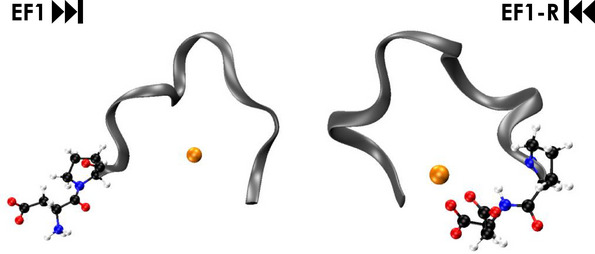
Representative snapshots from MD trajectory of EF1 (left) and EF1‐R (right) highlighting the role of proline in the DPD motif bending it toward (EF1‐R) or away (EF1) from the Eu(III). The grey ribbon depicts the peptide backbone, orange: europium, blue: nitrogen, black: carbon, red: oxygen, and white: hydrogen.

**Figure 7:**
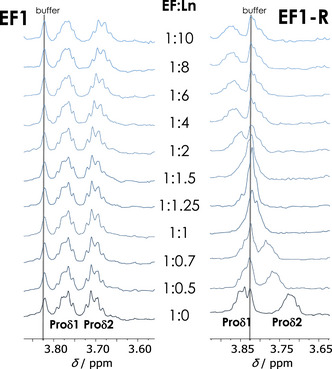
^1^H NMR Pro Hδ spectral regions showing the signals in the La(III) to peptide titration; EF1 (left) and EF1‐R (right), corresponding to the full data sets shown in Figures S36 and S40, respectively. The number of depicted titration steps was reduced for better signal traceability. The peptide to Ln(III) ratio of each step is shown in the middle of the figure.

Figure 6 shows representative MD results for EF1 and EF1‐R for Eu(III) which demonstrate that the C‐terminal DPD motif is bent toward the metal while in the forward peptides the N‐terminal DPD faces away from the metal. This is supported by the NMR titration experiments with EF1 and EF1‐R. In case of La(III) with EF1, the Hβ signals of Asp3, Asp5, and Asp9 (amino acids involved in La(III)‐coordination according to MD simulations, Table S16) shift whereas Hβ signals of the non‐coordinating Asp1 remain unaffected (Figure S35), underlining the results from MD simulations, that in the forward peptides the N‐terminal amino acid is not involved in Ln(III)‐binding. The proline Hδ signals proved to be excellent probes for the chemical environment around the DPD moiety by giving well observable, and separated signals around 3.7 ppm. For EF1, the proline Hδ signals remain virtually unaffected throughout the entire titration series (covering the formation of the 1:1 and 1:2 complexes) while in the case of EF1‐R significant alterations can be observed (Figure 7) indicating the bending of the DPD unit to and away from the metal, respectively.

These alterations of EF1‐R's Pro Hδ signals are qualitatively identical for both La(III) and Lu(III) (cf. Figures 7 (right) and S43 (middle)). Up to EF:Ln(III) ratios of ∼ 1:2, the two Pro Hδ signals shift toward each other, corresponding to a decreasing fraction of free peptide and increasing fraction of the 1:1 complex. While the free peptide's contribution to the mole fraction‐weighted signals decreases, the spectra increasingly reflect the 1:1 complex, with nearly isochronous Pro Hδ signals. However, upon further metal addition, the latter feature disappears and a new set of distinct Pro Hδ signals emerges due to displacement of the 1:1 by the 1:2 complex. This is much more explicit for the EF1‐R Lu(III) 1:2 complex as its Pro Hδ signals are significantly upfield shifted compared to those of the corresponding La(III) 1:2 complex showing a slight downfield shift. This is likely due to the different ionic radii of the respective Ln(III) and the concomitant impact on the peptide structure accommodating two metal ions in one complex. Taken together, the NMR results allow the following conclusions: (1) Regardless of the EF:Ln(III) ratio, EF1's N‐terminal Asp1 is not involved in coordination and, along with Pro2, facing away from the metal centre in any complex; (2) EF1‐R's C‐terminal Asp12 is indeed involved in coordination in both the 1:1 and the 1:2 complexes and, along with Pro11, inevitably facing toward the metal centre (see also Figures S79–S81). One should not neglect that in addition to the proline, the positive charge at the N‐terminus within the uncapped forward peptides might add to that effect by repelling the positively charged Lns. It has, for example, been shown that an acetylated N‐terminus is key for Ca(II) coordination in short EF‐hand peptides.^[^ ^39^ ^]^ After all, the involvement of the carboxylate sidechain of Asp1 in the metal coordination is usually the most conserved feature throughout Ca(II)‐binding proteins.^[^ ^37^ ^]^ And while in the so far reported lanmodulins an increasing amount of EF‐hand loops with Asn1 gets reported,^[^ ^14^, ^38^ ^]^ the DPD motif is fully involved in Ln‐binding in lanmodulin,^[^ ^14^, ^40^ ^]^ as the unit seems to be optimally positioned for Ln‐coordination, emphasising the importance of pre‐structuring and rigidity when it comes to higher binding affinity. In case of the investigated short peptides, the combination of a proline placed next a positively charged N‐terminus seems to decrease the involvement of the N‐terminal amino acid side chain altogether. Reversing the sequence and thus allocating proline close to the C‐terminus seems to be beneficial in order to increase the Ln‐affinity. As already mentioned above, in the calmodulin EF‐hand metal‐binding loops, of all possible positions it is in fact position 11 as the only one more frequently occupied by proline,^[^ ^37^ ^]^ just like in the investigated reverse peptides. Together with having acidic amino acids at the N‐ and C‐terminus and a Lys at position 2 (compare with literature^[^ ^37^ ^]^) those seem to be the only similarities between the sequences known for Ca‐binding EF‐hand loops and the reversed peptides.

### Asparagine versus Aspartate as C‐Terminal Amino Acid

**Figure 8:**
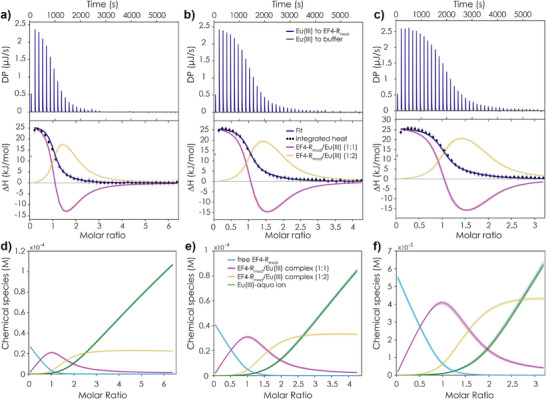
ITC data of EF4‐R_mod_ binding to Eu(III). Thermogram, integrated heat and used fit with a two‐component model obtained by titrating a 900 µM Eu(III) solution to a) 30 µM peptide b) 45 µM peptide and c) 60 µM peptide (conditions: pH 6.6, 25 °C, 10 mM MOPSO buffer, 100 mM KCl). For the three independently obtained data sets a global fit was used; the first titration point was excluded from the analysis. Chemical species distribution observed in the ITC titration experiments d) 900 µM Eu(III) to 30 µM peptide, e) 900 µM Eu(III) to 45 µM peptide and f) 60 µM peptide. Overlayed lines/ symbols represent 100 MC runs used for error estimation (see Supporting Information Section 3.2).

We observed similar affinities for EF4 and EF4‐R, both of which having the amino acid sequences with one less carboxylate residue (Asn instead of Asp) in comparison to the other peptides. We believe that the decreased number of carboxylates does not necessarily affect the overall metal‐binding affinity of EF4 because of the MD simulations showing that the N‐terminal amino acid is not part of the coordination of any forward peptide.^[^ ^17^ ^]^ That a higher content of acidic residues is not automatically associated with a higher Ln‐affinity, but can also lead to electrostatic repulsion has already been shown by systematic studies in the literature.^[^ ^1^, ^30^ ^]^ Also, the process of the development of potent Tb‐binding LBT by Imperiali and co‐workers showed that the ideal number of acidic amino acids within the sequence is 3–4.^[^ ^41^, ^42^ ^]^ In the reverse peptides, however, EF4‐R provides a C‐terminal DPN motif compared to the DPD motif of EF1‐R to EF3‐R, which played an important role in increasing the affinity of the latter peptides. MD simulations for EF4‐R incorporating Eu(III) (Figure S3, Table S15) showed that unlike the other reverse peptides, the C‐terminal amino acid is bent away from the metal centre and not involved in metal‐binding. EF4‐R is not only the one reverse peptide with an affinity similar to the forward peptides, but also showing a slightly different shape in the CD spectra, when compared to the other reverse peptides (Figure S30). To support the claim that a C‐terminal Asp is of importance, we modified EF4‐R and exchanged the N against a D in the sequence to yield the peptide EF4‐R_mod_, now carrying a DPD motif. This modified peptide exhibited the highest affinity of all investigated peptides so far – from worst to best with one modification. ITC and TRLFS both place the affinity toward Eu(III) for the 1:1 complex in the nM range (ITC: 150 ± 10 nM, TRLFS: 150 ± 50 nM, see Tables S7 and S8 for the values for the 1:2 complex and luminescence lifetimes). The corresponding ITC data for EF4‐R_mod_ is shown in Figure 8. This mutation places EF4‐R_mod_ within the range of some LBTs (15–20 amino acids long)^[^ ^43^ ^]^ which were stepwise optimised to obtain low nanomolar affinities.^[^ ^7^, ^42^, ^44^ ^]^ Significantly lower Ln‐affinities (i.e., low nanomolar to femtomolar) of short peptides have only be achieved by the introduction of synthetic unnatural aminoacids.^[^ ^1^, ^45^, ^46^, ^47^, ^48^ ^]^

### Investigation of the Lanthanide‐Selectivity

**Figure 9:**
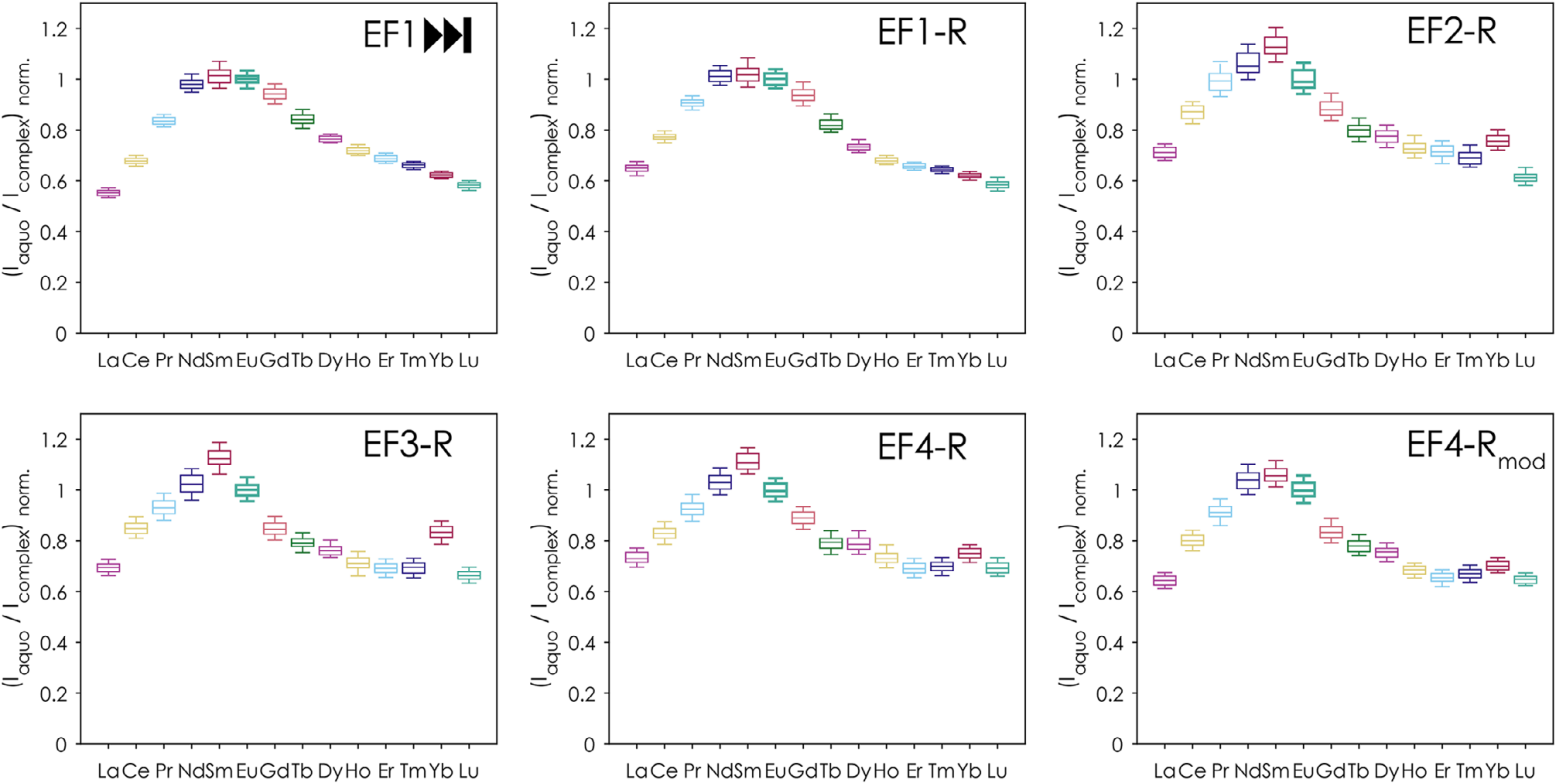
Relative affinities of the peptides to the different Ln(III) as ratio of the Eu(III)‐aquo ion and complexed Eu(III) obtained by a competition experiment with EuCl_3_ determined via TRLFS (pH 6.6, 10 mM MOPSO, 100 mM KCl). The relative affinities are shown as box plot (median and quartiles) and were obtained by PARAFAC in combination with a Monte Carlo approach as described in Section 3.3.2 in the Supporting Information.

In nature bacteria have shown to have a preference for the early elements in the lanthanide series.^[^ ^15^, ^49^, ^50^, ^51^, ^52^ ^]^ For LanM an especially high affinity for the middle of the lanthanide series (Nd, Sm, Eu) has been shown.^[^ ^53^ ^]^ To determine whether those short peptides have as well a preference for specific Lns, and if so, for which Lns the highest affinity can be observed, we employed a Ln‐competition experiment with TRLFS. We tested all Lns (except the radioactive promethium) of the series in comparison to Eu(III) by mixing each peptide with a 1:1 mixture of the respective LnCl_3_ and EuCl_3_. The samples were incubated overnight and then measured via TRLFS. To enable the data analysis of such single measurements without doing titrations for every Ln, the Eu(III) titration series described earlier were included in the data analysis as speciation basis. With this we were able to demonstrate that all peptides exhibit the highest affinity for Nd(III), Sm(III), and Eu(III) (see Figure 9), thus showing the same trends as observed for the native protein LanM measured under similar conditions.^[^ ^53^ ^]^

This trend is consistent for EF1, representing the forward peptides, and across the reverse peptides. Interestingly, all reverse peptides show a more or less pronounced dip at Yb(III), suggesting a higher affinity which breaks the trend of decreasing affinity from the middle of the series through Lu(III). To rule out concentration‐artefacts, all Ln‐competition samples were checked by inductively coupled plasma mass spectrometry (ICP‐MS) for their Ln‐content, confirming the intended Ln(III) concentration. Thus, a deviating Yb(III) concentration was not responsible for the observed dip which is still not yet fully understood. However, it is clear that from La(III) to Lu(III) no monotonous trend, simply following the lanthanide contraction and an increasing Lewis acidity – as seen for some other ligand systems^[^ ^54^, ^55^ ^]^ – can be observed.

## Conclusion

This study underscores the importance of serendipity in science and highlights the potential of unexpected discoveries in the field of peptide‐lanthanide interactions. By leveraging our initial peptide synthesis error, we gained valuable insights into key design principles for peptide‐lanthanide complexes, paving the way for the development of innovative de novo Ln‐binding materials and sustainable recycling strategies for these remarkable elements. We show that reversing the amino acid sequence of the metal‐binding loops of a native Ln‐binding protein significantly increases Ln‐affinity, while the selectivity trend across the lanthanide series remains largely consistent with that of the natural protein. Moreover, we identified key factors contributing to this affinity enhancement in short peptides: The possible involvement of the C‐terminal carboxylate in a succinate‐like chelation motif and the strategic placement of structure‐inducing amino acids such as proline. The integration of spectroscopic (CD, NMR, TRLFS), thermodynamic (ITC), and computational (MD) methods provided a coherent and multidimensional understanding of the peptide–lanthanide interactions, enabling the identification of affinity‐enhancing structural features.

Finally, we demonstrate that a single‐point amino‐acid mutation within a reversed sequence can result in a bio‐inspired peptide with a Ln(III)‐affinity of approximately 150 nM – an affinity range rarely achieved by uncapped bio‐inspired peptides of similar length. These findings expand the toolbox of sequences available for future bio‐inspired lanthanide‐binding applications.

## Author Contributions

**S.M.G.T**. and **L.J.D**. conceptualised the idea and wrote the initial draft of the manuscript. **S.M.G.T., B.D**., and **L.J.D**. acquired funding. **S.M.G.T**, **J.K**. and **B.D**. performed experiments and analysed data. **S.T**. performed molecular dynamic simulations and provided the interpretation. The necessary resources and infrastructure for this work was provided by **L.J.D**., **B.D**., and **R.S**. All authors were involved in reviewing and editing this manuscript.

## Supporting Information

The authors have cited additional references within the Supporting Information.^[^ ^56^, ^57^, ^58^, ^59^, ^60^, ^61^, ^62^, ^63^, ^64^ ^]^

## Conflict of Interests

The authors declare no conflict of interest.

## Supporting information



Supporting Information

## Data Availability

Raw data files from NMR (.fid), TRLFS (.sif), ITC (.itc), and CD (.jws) experiments, along with .xyz and .pdb files of the molecular dynamics simulations are available in the RODARE repository (DOI: 10.14278/rodare.3264).
