# Supplementary material for: Reversing Lanmodulin's Metal‐Binding Sequence in Short Peptides Surprisingly Increases the Lanthanide Affinity
Source: Angew Chem Int Ed Engl. 2025 Sep 23;64(46):e202510453. doi: 10.1002/anie.202510453 (PMC12603986; doi:10.1002/anie.202510453)
Supplement: Supplementary file 1 — Supporting Information [file ANIE-64-e202510453-s001.pdf]

Supporting Information

Reversing Lanmodulin's Metal-binding Sequence in Short Peptides Surprisingly Increases the Lanthanide Affinity

Sophie M. Gutenthaler-Tietze[a,c], Jerome Kretzschmar[b], Satoru Tsushima[b,d], Robin Steudtner[b], Björn Drobot[b], Lena J. Daumann*[c]*

[a] Department of Chemistry, Ludwig-Maximilians-Universität München, Butenandtstraße 5-13, 81377 München (Germany)
[b] Institute of Ressource Ecology, Helmholtz-Zentrum Dresden-Rossendorf e.V. Bautzner Landstraße 400, 01328 Dresden (Germany)
[c] Chair of Bioinorganic Chemistry, Heinrich-Heine-Universität Düsseldorf, Universitätsstraße 1, 40225 Düsseldorf (Germany)
[d] Institute of Integrated Research, Institute of Science Tokyo, 152-8550, Tokyo (Japan)

Table of Contents

1 Solvents and Chemical Reagents 3

1.1. Ordered Peptides 3

2 Peptide Synthesis, Purification, Purity, and Concentration Determination 4

2.1 Solid Phase Peptide Synthesis (SPPS) and Purification *via* preparative RP-HPLC 4

2.2 Purity and Concentration Determination 4

3 Lanthanide-binding Studies 5

3.1 Metal Stocks 5

3.2 Isothermal Titration Calorimetry (ITC) 5

3.3 Time-resolved Laser-induced Fluorescence Spectroscopy (TRLFS) 6

3.3.1 Peptide to Eu(III) and Eu(III) to Peptide Titration – Combined Titration Sets: 7

3.3.2 Ln-competition Experiment: 8

3.4 Circular dichroism (CD) Spectroscopy 9

3.5 Nuclear Magnetic Resonance (NMR) Spectroscopy 9

4 Molecular dynamics (MD) simulations 10

5 Supplementary Tables and Figures 11

5.1 Supplementary ITC Data 11

5.2 Supplementary TRLFS Data 19

5.3 Supplementary CD Data 24

5.4 Supplementary NMR Data 26

5.5 Supplementary MD Data 55

6 Data Availability 57

7 References 57

1 Solvents and Chemical Reagents

For all experiments ultrapure water (type 1, pH 5.6, 18.2 MΩ·cm at 25 °C) was used. Therefore, demineralised water was further purified using a Synergy® UV system from Merck Millipore®. All chemicals were used as supplied, unless stated otherwise, and handled according to potential hazards and toxicity.

1.1. Ordered Peptides

Table S1 Molecular weights and amino acid sequences of ordered lanmodulin-inspired peptides.

Peptide	Molecular Weight [g/mol]	Sequence
EF4-R	1316.29	H-ERADITGDNDPN-OH
EF1-OMe	1359.44	H-DPDKDGTIDLKE-OMe
EF1-R-OMe	1359.44	H-EKLDITGDKDPD-OMe
EF4-R_{mod}	1317.28	H-ERADITGDNDPD-OH

Peptides were ordered by *GenScript* and used as delivered by preparing 2 mg/mL gross weight stocks in ultrapure water. The target peptide concentration was calculated by taking the analytical results provided by *GenScript* (see Table S2) into account using equation (1).

$$\left(\frac{gross\ weight\ conc.\ \left[\frac{mg}{mL}\right]\cdot AA\ content\ [\%]\cdot LCMS\ purity[\%]}{molecular\ weight\ \left[\frac{mg}{mmol}\right]}\right)\cdot 10^6 = target\ peptide\ conc.\left[\frac{\mu mol}{L}\right] \quad (1)$$

Table S2 Purity and calculated target peptide concentration in µM of the peptides ordered from GenScript.

Peptide	AA content (%)	HPLC purity (%)	LCMS purity (%)	Target peptide conc (µM)
EF4-R	78.85	98.54	78.87	945
EF1-OMe	30.25	95.50	92.81	413
EF1-R-OMe	77.59	98.83	92.08	1051
EF4-R_{mod}	83.12	95.03	95.23	1202

2 Peptide Synthesis, Purification, Purity, and Concentration Determination

Table S3 Molecular weights and amino acid sequences of the synthesised peptides.

Peptide	Molecular Weight (g/mol)	Sequence
EF1-R	1345.43	H-EKLDITGDKDPD-OH
EF2-R	1303.35	H-EKADLTGDKDPD-OH
EF3-R	1346.37	H-EKKDLTGDNDPD-OH

2.1 Solid Phase Peptide Synthesis (SPPS) and Purification *via* preparative RP-HPLC

The peptides (see Table S3) were obtained by automated microwave-assisted solid phase peptide synthesis as described here.[1] The synthesised peptides were purified *via* RP-HPLC on an *Agilent* 1260 II system (G1364E 1260 FCPS, G7165A 1260 MWD, G7161A Prep Bin Pump) with a *Dr. Maisch* ReproSil-Pur 120 C18-AQ column (250 mm × 20 mm, 5 µm). Only HPLC grade solvents were used. The detection wavelength was 210 nm and the used methods for each peptide are shown below. The synthesis, purification, and concentration determination for EF1, the forward peptide included for comparison, was described elsewhere.[1]

Peptide EF1-R:
Eluent system: water/acetonitrile + 0.1% TFA; flow rate: 20 mL/min.
Method: 5 min isocratic at 5% acetonitrile, then 5% to 25% acetonitrile in 30 min; t_R = 27.4 min.

Peptide EF2-R:
Eluent system: water/acetonitrile + 0.01% TFA; flow rate: 20 mL/min.
Method: 5 min isocratic at 5% acetonitrile, then 5% to 9% acetonitrile in 18.4 min; t_R = 18.5 min

Peptide EF3-R:
Eluent system: water/acetonitrile + 0.05% TFA; flow rate: 20 mL/min.
Method: 5 min isocratic at 5% acetonitrile, then 5% to 11% acetonitrile in 31 min; t_R = 28.7 min.

2.2 Purity and Concentration Determination

The purity and concentration determination of the peptides EF1-R, EF2-R, and EF3-R was performed analogously as described earlier[1] with a combination of analytical RP-HPLC, HPLC-HRMS measurements and the Pierce quantitative fluorometric peptide assay. The analytical HPLC traces which were used for the HPLC purity determination given in Table S4 are shown in Figure S1. The determined net peptide concentration together with the calculated target peptide concentration is summarised in Table S5.

Table S4 Exact mass, HPLC retention time (UV trace), and purity determined by HPLC of the synthesised peptides.

Peptide	Retention time (min)	HPLC Purity (%)	*m/z* [M+H]$^+$ calc.	*m/z* [M+H]$^+$ obs.
EF1-R	18.0	97.1	1345.6482	1345.6476 (err. −0.45 ppm)
EF2-R	12.9	96.5	1303.6012	1303.5990 (err. −1.69 ppm)
EF3-R	13.7	100	1346.6070	1346.6040 (err. −2.23 ppm)

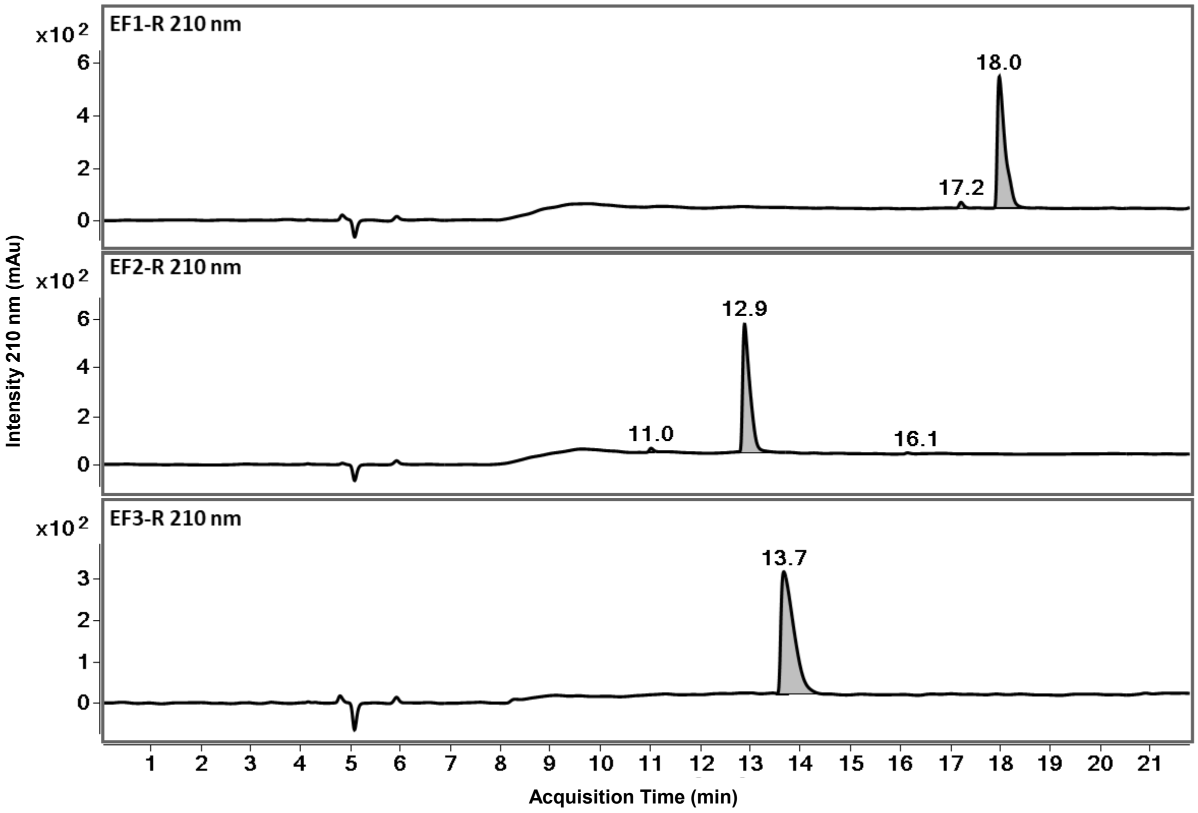

Figure S1 Integrated HPLC chromatograms of EF1-R, EF2-R, and EF3-R. HPLC method for EF1-R and EF2-R: 5 min isocratic at 95/5% H_2O/MeCN + 0.1% FA, then in 18 min from 5 to 20% MeCN followed by a 1 min long isocratic step at 20% acetonitrile. HPLC method for EF3-R: 2 min isocratic at 95/5% H_2O/MeCN + 0.1% FA, then in 18 min from 5% to 10% MeCN.

Table S5 Mean net peptide concentration with standard deviation in µg/mL and target peptide concentration with standard deviation derived from the mean net peptide concentration in µM.

Peptide	Mean Net Peptide Concentration (µg/mL)	Target Peptide Concentration (µM)
EF1-R	869 ± 39	844 ± 28
EF2-R	1023 ± 35	988 ± 26
EF3-R	890 ± 29	890 ± 21

3 Lanthanide-binding Studies

3.1 Metal Stocks

All metal-binding experiments were performed with $LnCl_3$. For this $LnCl_3 \cdot n\,H_2O$ solutions were prepared as 10 mM stock solutions in ultrapure water (type 1, pH 5.6, 18.2 MΩ·cm at 25 °C) if not stated otherwise and stored at −20 °C until usage.

3.2 Isothermal Titration Calorimetry (ITC)

For ITC titration experiments, a *Malvern Panalytical* MicroCal PEAQ-ITC instrument was used. The reference cell was filled with water, the stirring speed set to 750 rpm and the initial delay to 60 s. Titrations were performed over 39 steps (first injection: 0.4 µL, residual injections: 1 µL) in time intervals of 150 s. For all peptides, three measurements were conducted in which the peptide solution

was in the cell using the same settings, but changing the concentration in the cell (30 µM, 45 µM, 60 µM) while keeping the concentration of a $EuCl_3$ solution in the syringe constant (900 µM). The buffer and salt concentration (pH 6.6, 10 mM MOPSO, 100 mM KCl) were kept constant. The cell was equilibrated with the buffer before the titration and the syringe with the used $EuCl_3$ solution in the same buffer. Analogously performed titrations using the same settings, but with buffer in the cell were performed and used as background measurement. The obtained data was analysed with MATLAB using a workflow in which the raw ITC data were pre-treated to reduce noise using singular value decomposition (SVD) as shown in Figure S2 and described elsewhere[1]. To increase the robustness of the fit, the replicates were evaluated globally[2] using a Monte Carlo (MC) approach.[3] ITC and TRLFS data were analysed assuming the same model which was developed taking into account the results of both methods. The Gibbs free energy (ΔG) and entropy contribution (TΔS) were calculated from the obtained enthalpy (ΔH) and affinity (K_D) values (Table S6).

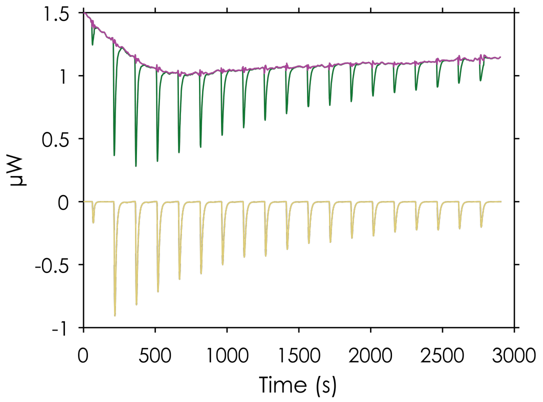

Figure S2 Exemplary representation of the baseline removal. The baseline with noise (magenta) is subtracted from the raw data (green) to obtain the adjusted data (yellow) for the integration of the individual titration steps.

Ca(II) titration experiments were performed analogously using the same buffered system and setup as single measurements with 30 µM peptide in the cell and 9 mM $CaCl_2$ in the syringe. A control measurement was performed as 9 mM $CaCl_2$ to buffer titration. The data was baseline corrected using the *Malvern* MicroCAl PEAQ-ITC Analysis Software.

3.3 Time-resolved Laser-induced Fluorescence Spectroscopy (TRLFS)

All titration experiments were performed in MOPSO buffer (10 mM, 100 mM KCl, pH 6.6) at 25 °C in stirred (1200 rpm) 2 mL 10 mm pathlength *Hellma Analytics* quartz cells equipped with a screw cap and septum. The cuvette was placed in a cuvette holder which was connected *via* a light guide to a spectrograph (*Andor*, SR-303i-A). For recording the spectra an intensified CCD (Andor iStar, DH320T-18U-63) was used. For Eu(III) the excitation wavelength (*Ekspla*, NT230, ~5 ns pulse) was set to 394 nm (grating: 300 l/mm). The experimental parameters were as follows: linear increasing step size: 3+3·x µs; initial delay: 12 µs (to prevent $^5D_1 \rightarrow {^7F_j}$ transitions), gate width: 100 µs; slit width: 200 µm; accumulations: 200; kinetic series length: 21; gain: 4095, or varied during the experiment. All data sets were analysed using MATLAB. For this the raw data were loaded using the *Andor* sif-reader.[4] Detailed data analysis was performed with parallel factor analysis (PARAFAC) using the N-way toolbox for MATLAB[5] with modification described elsewhere.[6,7] For the speciation analysis an adopted version of a MATLAB script from Smith[8] was used. Error estimations were performed by using a Monte Carlo (MC) approach.[3] More details on the specific analysis work-flow for the different experimental setups can be found below.

3.3.1 Peptide to Eu(III) and Eu(III) to Peptide Titration – Combined Titration Sets:

Titrations consisted of 37 steps, in the first 20 steps a titrant solution containing the respective peptide (250 µM peptide, containing 5 µM Eu(III)) was added stepwise to a cuvette containing Eu(III) (5 µM Eu(III), 1 mL) varying from 0 µM peptide to 64.4 µM peptide, the gain was constant at 4095. Then a second titrant solution containing Eu(III) (2500 µM Eu(III), 64.4 µM peptide) was added to this mixture over 17 steps to increase the [Eu(III)] from 5 µM to 756.2 µM while keeping the peptide concentration constant; the gain was lowered as needed and logged to be included in the analysis; the buffer and salt concentration were held constant during the whole series. The setup and a representative titration scheme are shown in Figure S3, insights into the data analysis are shown in Figure S4.

Cuvette	$EuCl_3$	5 µM	initial V	1 mL		V to prepare:	1 mL
Titrant 1	EF	250 µM	including	5 µM EuCl3		V to prepare:	370 µL
Titrant 2	$EuCl_3$	2500 µM	including	64.4 µM EF		V to prepare:	600 µL

Buffer =	10 mM MOPSO
I =	100 mM KCl
pH =	6.6 (25°C)

Sample	V cuvette (µL)	V $EuCl_3$ total (µL)	V $EuCl_3$ add (µL)	V EF total (µL)	V EF add (µL)	c EF (µM)	c $EuCl_3$ (µM)	Laser (mJ)
01	1000.0	0	0	0.0	0.0	0.0	5.0	
02	1001.0	0	0	1.0	1.0	0.3	5.0	
03	1002.5	0	0	2.5	1.5	0.6	5.0	
04	1005.5	0	0	5.5	3.0	1.4	5.0	
05	1010.1	0	0	10.1	4.6	2.5	5.0	
06	1016.3	0	0	16.3	6.2	4.0	5.0	
07	1024.1	0	0	24.1	7.8	5.9	5.0	
08	1033.6	0	0	33.6	9.5	8.1	5.0	
09	1044.9	0	0	44.9	11.3	10.8	5.0	
10	1058.2	0	0	58.2	13.3	13.8	5.0	
11	1073.5	0	0	73.5	15.3	17.1	5.0	
12	1091.1	0	0	91.1	17.6	20.9	5.0	
13	1111.1	0	0	111.1	20.0	25.0	5.0	
14	1133.8	0	0	133.8	22.7	29.5	5.0	
15	1159.4	0	0	159.4	25.6	34.4	5.0	
16	1188.4	0	0	188.4	28.9	39.6	5.0	
17	1221.0	0	0	221.0	32.6	45.3	5.0	
18	1257.9	0	0	257.9	36.9	51.3	5.0	
19	1299.5	0	0	299.5	41.7	57.6	5.0	
20	1346.8	0	0	346.8	47.3	64.4	5.0	
21	1348.5	1.7	1.7	346.8	0.0	64.4	8.2	
22	1351.5	4.7	3.0	346.8	0.0	64.4	13.7	
23	1357.5	10.7	6.0	346.8	0.0	64.4	24.7	
24	1366.6	19.8	9.1	346.8	0.0	64.4	41.2	
25	1379.0	32.2	12.3	346.8	0.0	64.4	63.2	
26	1394.7	47.9	15.7	346.8	0.0	64.4	90.7	
27	1414.1	67.3	19.4	346.8	0.0	64.4	123.7	
28	1437.4	90.6	23.3	346.8	0.0	64.4	162.2	
29	1464.9	118.1	27.6	346.8	0.0	64.4	206.2	
30	1497.3	150.5	32.3	346.8	0.0	64.4	255.7	
31	1534.9	188.1	37.6	346.8	0.0	64.4	310.7	
32	1578.5	231.7	43.6	346.8	0.0	64.4	371.2	
33	1629.0	282.2	50.5	346.8	0.0	64.4	437.2	
34	1687.5	340.7	58.5	346.8	0.0	64.4	508.7	
35	1755.4	408.6	67.9	346.8	0.0	64.4	585.7	
36	1834.4	487.6	79.1	346.8	0.0	64.4	668.2	
37	1927.0	580.2	92.6	346.8	0.0	64.4	756.2	

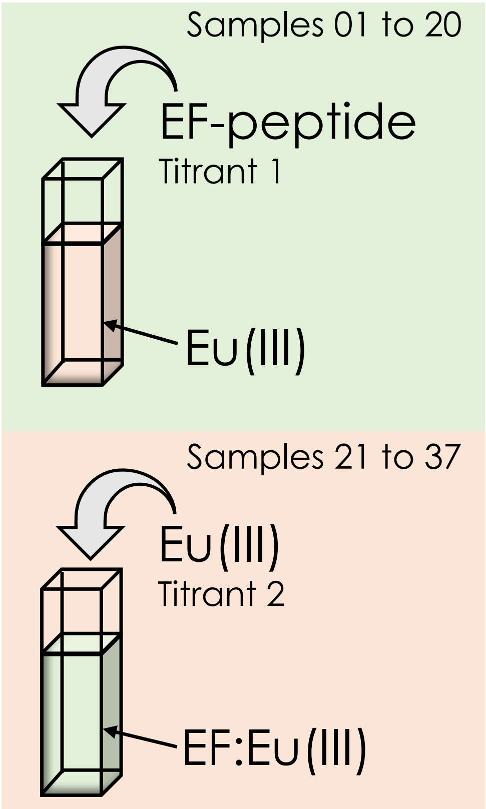

Figure S3 Titration scheme (left) and visual representation (right) of the combined titration setup in which the respective peptide (in titrant 1) is added to a buffered Eu(III) solution in increasing amounts (Samples 0-20). Concentration of buffer, salt, and Eu(III) are kept constant by using the same concentration in both the cuvette and titrant 1. After an excess of peptide was added, the Eu(III) concentration gets increased (Samples 21-37) by adding titrant 2. Then, instead of the Eu(III) concentration the peptide concentration (of Sample 20) was kept constant by using the same concentration in titrant 2.

For data analysis it was important to take into account, that as [Eu(III)] increases in the second subset, the intensity in this section also increases significantly (see Figure S4E). For PARAFAC, this means a higher weighting of the second part. To balance both subsets, we applied a weighting factor to the second part for intensity equalisation (see Figure S4F) and then conducted a global analysis of the complete data set using PARAFAC including a speciation restriction, taking the weighting factor into account.

This approach yielded affinity parameters and emission spectra for the entire data set as well as species distributions for each subset.

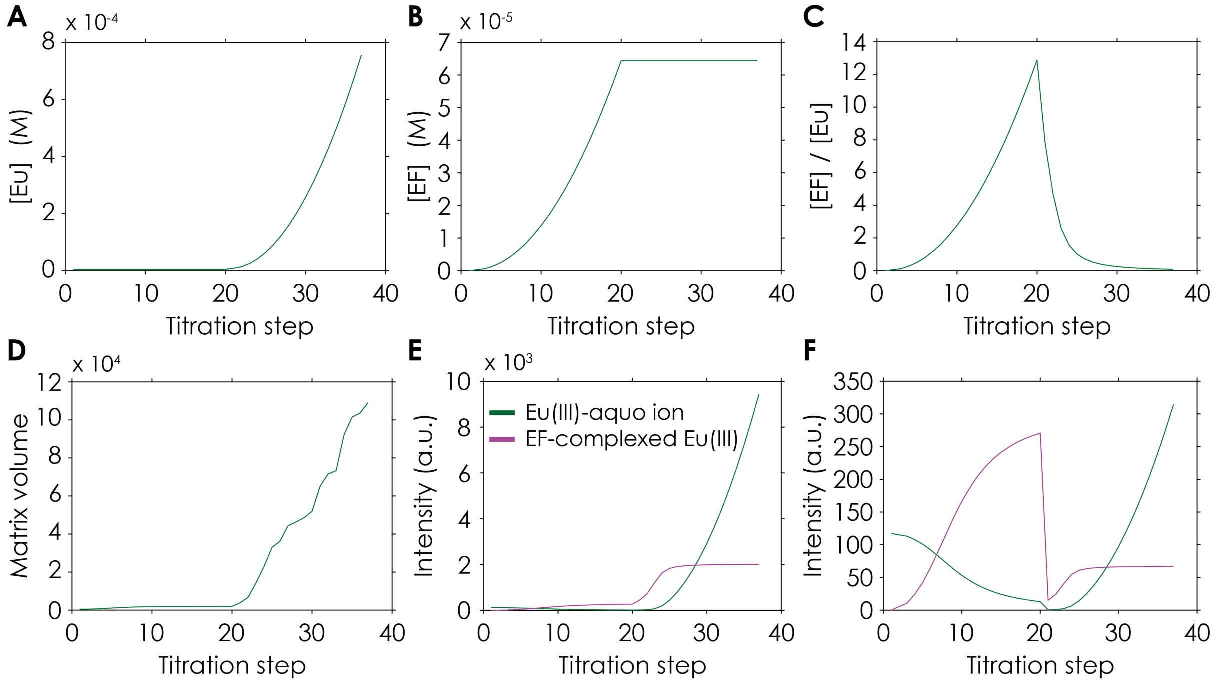

Figure S4 Schematic representation of the combined titration of peptide to Eu(III) (steps 1 to 20) with subsequent titration of Eu(III) (steps 21 to 37). The figures in the top row illustrate **(A)** the development of the metal concentration, **(B)** the titration-dependent peptide concentration and **(C)** the resulting peptide to Eu(III) ratio. **(D)** shows the resulting evolution of the total luminescence intensity after gain correction which leads to **(E)** a distribution of complexed and uncomplexed europium. **(F)** Since such a distribution would give the second part of the titration (increase in the europium concentration) a disproportionately higher weighting in a factor analysis compared to the first part, the intensity of the second part was reduced before the factor analysis. This was taken into account in the speciation evaluation.

3.3.2 Ln(III) *vs*. Eu(III) Competition Experiment:

Individual samples of the respective peptides with the different Lns(III) (La-Lu, except Pm) were mixed in 1.5 mL centrifuge tubes (40 µM Eu(III), 40 µM Ln(III), 10 µM peptide, 10 mM MOPSO, 100 mM KCl, pH 6.6), incubated at 6 °C overnight, transferred into cuvettes and measured with the parameters described above.

To take full advantage of the benefits of PARAFAC, the species must differ from each other in all dimensions (spectra, luminescence decay, and distribution). Since the ratio of species (Eu(III) aquo ion and Eu(III) complexed) is very similar for the different Lns in the distribution dimension, this requirement is not met. Therefore, we combined the competition series with the previously described series for determining the K_D values of Eu(III) described in Section 3.3.1 to ensure sufficient differences between the species in the distribution dimension as well (Figure S5A). Under the given concentration ratios of metal and peptide, different Eu(III)-peptide complexes are formed, varying in stoichiometry and metal composition. To reduce complexity, the data analysis focused solely on the Eu(III) aquo ion and peptide-complexed Eu(III). In the second species, the various Eu(III) complexes were clustered. Under these conditions, PARAFAC was performed in combination with a Monte Carlo (MC) approach (Figure S5B). Thereby we obtained a relative affinity of the peptides for the various lanthanides which is defined as the intensity ratio of the Eu(III)-aquo ion and the peptide-complexed Eu(III). For comparability between the peptides, the values were normalised to the mean of the Eu(III) sample for

each peptide (Figure S5C). The intensity distributions for the different peptides are shown in Figure S26 to S28 and the normalised relative affinities in Figure 7, S27 and S28.

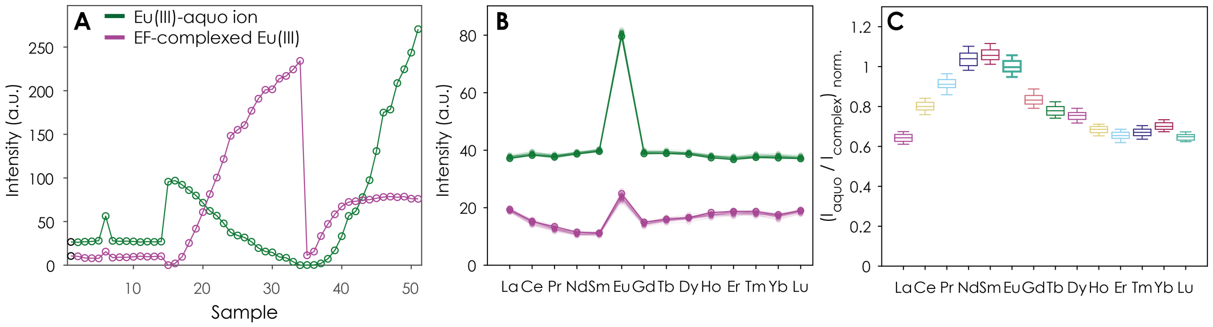

Figure S5 Schematic representation of the competition analysis. To ensure sufficient variance in the distribution and the same Eu(III) species, the Ln samples were combined with the samples of the 37-step titration sets described in Section 3.3.1. **(A)** Distribution of the Eu(III)-aquo ion and complexed Eu(III) in the competition experiment (Samples 1 to 14) as well as the combined titration series (Samples 15 to 51). **(B)** Distribution of the Ln competition subset after MC PARAFAC. **(C)** Final plot showing the relative affinity as the intensity ratio of the Eu(III)-aquo ion and the complexed Eu(III).

3.4 Circular dichroism (CD) Spectroscopy

CD spectra were recorded in a *Hellma Analytics*® quartz cell (110-QS, 1 mm pathlength) on a *JASCO* 1500 CD spectropolarimeter in MOPSO buffer (10 mM, 100 mM KCl, pH 6.6) at 25 °C between 190 nm and 250 nm. The measuring parameters were the following: scanning speed: 50 nm/min, data integration time: 4 s, data pitch: 0.5 nm, bandwidth: 1 nm and number of accumulations: 3. A recorded spectrum of the buffer was subtracted from all obtained spectra using the *Spectragryph* software[9]. Lanthanide titration experiments were performed by adding a 1 mM lanthanide chloride solution in increasing amounts (9 steps) to a solution containing 60 µM of the respective peptide until a 5-fold excess was reached. For binding experiments with Ca(II) a 1 M calcium chloride solution was added in increasing amounts (4 steps) to a solution containing 60 µM of the respective peptide to reach a 1000-fold excess. To avoid dilution effects, the buffer and peptide concentration were kept constant in all experiments. The measured ellipticity in mdeg was converted to molar ellipticity (θ) as described previously.[1] Data was plotted with MATLAB.

3.5 Nuclear Magnetic Resonance (NMR) Spectroscopy

NMR spectra were recorded on an *Agilent* DD2-600 14.1 T system corresponding to 599.8 and 150.8 MHz ^{1}H and ^{13}C resonance frequencies, respectively, using a 5 mm oneNMR™ probe. D_2O containing 0.03% TMSP-d_4 (99.95%) was purchased from *Deutero* and MES-d_{13} from *Cambridge Isotope Laboratories*. If not stated otherwise, the measurements were performed at 25 ± 1 °C in 30 mM MES-d_{13} puffer with 100 mM KCl in water with 10% D_2O and 0.003% TMSP-d_4 at pH = 6.6 (pH carefully adjusted using NaOD (99% D, *Deutero*)) applying water signal suppression (see below). All stock solutions of $LnCl_3$ and peptides were directly prepared in the described buffer. Spectra were, if not stated otherwise, referenced internally relative to the $Si(Me)_3$ signal of TMSP-d_4 with $\delta = 0$ ppm. The setup for ^{1}H NMR Ln-titration experiments was as followed: The respective $LnCl_3$ was added stepwise over 14 additions (0–2 mM) to a solution containing 200 µM peptide; the buffer, salt and peptide concentration were held constant.

For the determination of the chemical shifts of the respective peptide without added lanthanide twice as high concentrated samples were used and ^{1}H NMR and 2D (^{1}H,^{1}H-TOCSY, ^{1}H,^{13}C-HMBC, ^{1}H,^{13}C-HSQC) NMR experiments were performed. After signal assignment in H_2O/ D_2O (9:1) the samples were redissolved in pure D_2O and measured again in order to increase the resolution. The combination of the H_2O/ D_2O and D_2O spectral data enabled the full assignment of all shifts of the analysed peptides. Besides the collapse of NH-related couplings upon NH–ND exchange affecting splitting patterns, signal positions are virtually identical in both aqueous solvents, as repeatedly verified by superimposing corresponding spectra. ^{1}H NMR spectra were recorded using solvent suppression techniques; that is, choosing from "presat" (pre-saturation upon narrow band excitation on the water resonance for 2 s (1D) or 1 s (2D), or "WET" (water suppression enhanced through T_1 effects) occasionally combined with CPMG echo-train introducing T_2 relaxation filter. The most suitable method was individually figured out for each sample, and then applied to both the 1D-^{1}H as well as the corresponding 2D correlation experiments. 1D-^{1}H spectra were obtained upon applying a 2.8 μs (π/6) pulse followed by 3 s acquisition time and 3 s relaxation delay, accumulating at least 128 scans. ^{1}H–^{13}C heteronuclear single-quantum coherence (HSQC) and ^{1}H–^{13}C heteronuclear multiple-bond correlation (HMBC) sequences applied gradient-selection and adiabatic pulses, acquiring 2048 × 512 and 2048 × 1024 complex points in F_2 and F_1. Per F_1 increment, between 80 and 176 (HSQC) and 160 and 240 (HMBC) individual spectra were accumulated with a relaxation delay of 1 s. For polarization transfer, $(2\times J)^{-1}$ delays of 3.85 and 62.5 ms were opted, corresponding to 130 Hz ^{1}J in HSQC and 8 Hz ^{n}J in HMBC, respectively. Zero-quantum-filtered total correlation (zTOCSY) and rotating-frame Overhauser-enhancement spectroscopy (ROESY, using adiabatic pulses) experiments were performed upon acquisition of 2048 × 256 complex points in F_2 and F_1, accumulating at least 64 individual spectra per F_1 increment, applying 80 and 300 ms mixing time, respectively, and a relaxation delay of 1 s. All NMR spectra were processed using MestReNova (12.0.2).

4 Molecular dynamics (MD) simulations

MD simulations and data analyses were performed using AMBER 15 program package[10] with ff99SB force field applied on the peptide. For Ln(III) (Ln = La, Eu, Lu), 12-6-4 Lennard-Jones-type parameters developed by Merz *et al.* were employed.[11] Initial structures of reverse peptides were constructed as pure "string" without introducing any secondary structure element and without including any Ln(III) ion, but including sodium(I) as counterions and were equilibrated for 200 to 300 ns. After equilibration, one or two Ln(III) ions were placed with the "folded" peptide and again the system was freely equilibrated.

The protonation state of the peptides was adjusted to model physiological pH. Then, TIP3P waters were added with a minimum water layer thickness of 10 Å. 500 steps of steepest descent and 500 steps of conjugate gradient – with 500 kcal mol^{-1} $Å^{-1}$ harmonic restraint on the peptides – was initially conducted, after which 1000 steps of steepest descent and 1500 steps of conjugate gradient were performed without constraints. 40 ps of heating of the system from 0 to 300 K with 10 kcal mol^{-1} $Å^{-1}$ harmonic restraint on the peptides, after which another 1 ns preconditioning run was performed at 300 K without restraint on the solutes. Finally, a 200 to 350 ns MD simulation run was performed in a periodic boundary condition in an NPT ensemble from which the last 100 ns trajectory were taken. Simulations were terminated and restarted every 5 ns. The SHAKE algorithm, a 2 fs time integration step, 12 Å cut-off for non-bonded interactions, and the particle mesh Ewald (PME) method were used. MD trajectory was recorded at every 50 ps. When counting coordination numbers, oxygen atoms within 2.7 Å distance from metal centres were defined as coordinated atoms.

5 Supplementary Tables and Figures

5.1 Supplementary ITC Data

Table S6 Supplementary values to Table 1 of the main manuscript; n (used as concentration correction factor of the cell concentration during the analysis), ΔH values of the global analysis of three independent Eu(III) to peptide ITC titration experiments with different peptide concentrations in the cell. ΔG and TΔS were calculated using the ΔH and the obtained K_D value; the given uncertainties are the result of the used Monte Carlo approach.

		**EF-Eu(III) 1:1 complex**			**EF-Eu(III) 1:2 complex**		
	n	ΔH (kJ/mol)	ΔG (kJ/mol)	TΔS (kJ/mol)	ΔH (kJ/mol)	ΔG (kJ/mol)	TΔS (kJ/mol)
**EF1-R**	1.01±0.02	23.1±0.2	−35.9±0.2	58.9±0.2	29.0±0.7	−28.3±0.1	57.3±0.7
**EF2-R**	1.04±0.01	24.0±0.2	−35.3±0.1	59.3±0.1	29.3±0.5	−27.8±0.1	57.0±0.5
**EF3-R**	1.18±0.01	25.4±0.1	−37.2±0.1	62.6±0.1	28.1±0.3	−28.7±0.1	56.8±0.3
**EF4-R**	1.10±0.03	25.0±0.2	−28.9±0.2	53.9±0.2	29.9±1.0	−23.2±0.4	53.1±1.4

Table S7 K_D values, n (used as concentration correction factor of the cell concentration during the analysis), and ΔH values of the global analysis of three independent Eu(III) to peptide ITC titration experiments with different peptide concentrations in the cell. ΔG and TΔS were calculated using the ΔH and the obtained K_D value; The given uncertainties are the result of the used Monte Carlo approach.

		**EF-Eu(III) 1:1 complex**				**EF-Eu(III) 1:2 complex**			
	n	K_D (µM)	ΔH (kJ/mol)	ΔG (kJ/mol)	TΔS (kJ/mol)	K_D (µM)	ΔH (kJ/mol)	ΔG (kJ/mol)	TΔS (kJ/mol)
**EF1-OMe**	0.85±0.01	17.0±1.2	21.1±0.5	−27.2±0.2	48.3±0.3	-	-	-	-
**EF1-R-OMe**	1.09±0.04	31.8±2.1	20.0±0.9	−25.7±0.2	45.6±0.7	-	-	-	-
**EF4-R$_{mod}$**	0.95±0.01	0.15±0.01	25.6±0.1	−39.0±0.1	64.6±0.2	6.0±0.3	29.3±0.3	−29.8±0.1	59.2±0.3

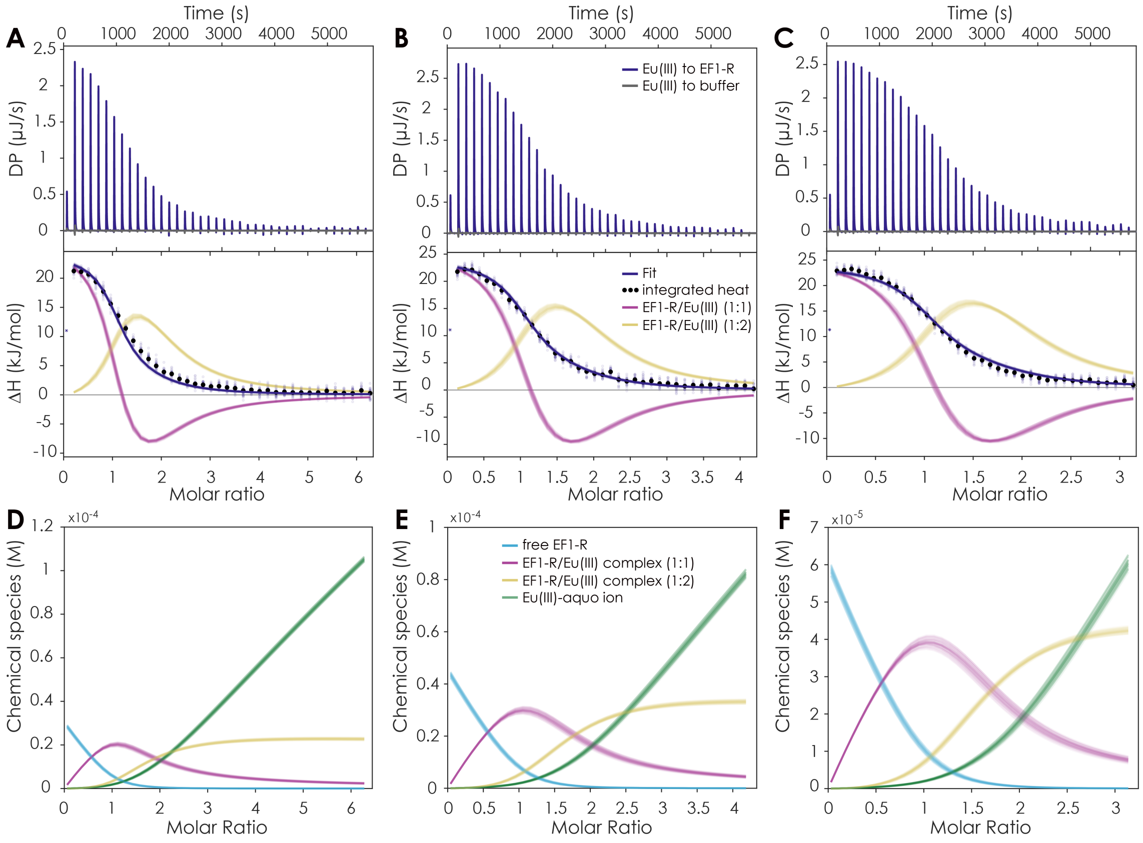

Figure S6 ITC data of EF1-R binding to Eu(III). Thermogram, integrated heat, and used fit with a two-component model obtained by titrating a 900 µM Eu(III) solution to **(A)** 30 µM peptide **(B)** 45 µM peptide and **(C)** 60 µM peptide. For the three independently obtained data sets a global fit was used; the first titration point was excluded from the analysis. Chemical species distribution observed in the ITC titration experiments **(D)** 900 µM Eu(III) to 30 µM peptide, **(E)** 900 µM Eu(III) to 45 µM peptide and **(F)** 60 µM peptide. Experimental conditions: pH 6.6, 25 °C, 10 mM MOPSO buffer, 100 mM KCl. Overlayed lines/ symbols represent 100 MC runs which were used for error estimation (see Section 3.2).

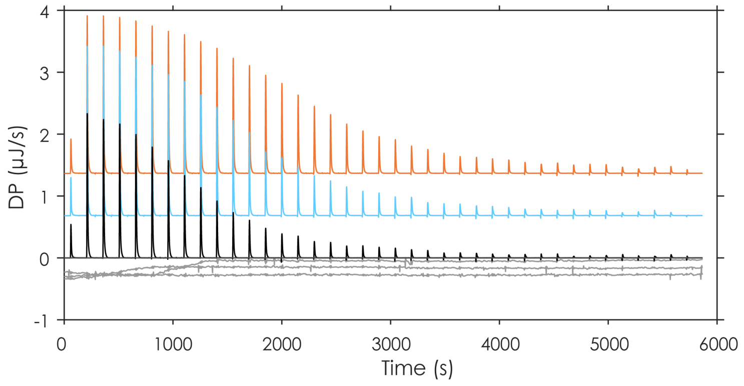

Figure S7: SVD-corrected thermograms of the Eu(III) to EF1-R titrations (orange: 60 µM set, blue: 45 µM set, black: 30 µM set) shown in Figure S6; the residuals for all three titrations are shown in grey. For better visualisation, the data was stacked with an offset to the x-axis.

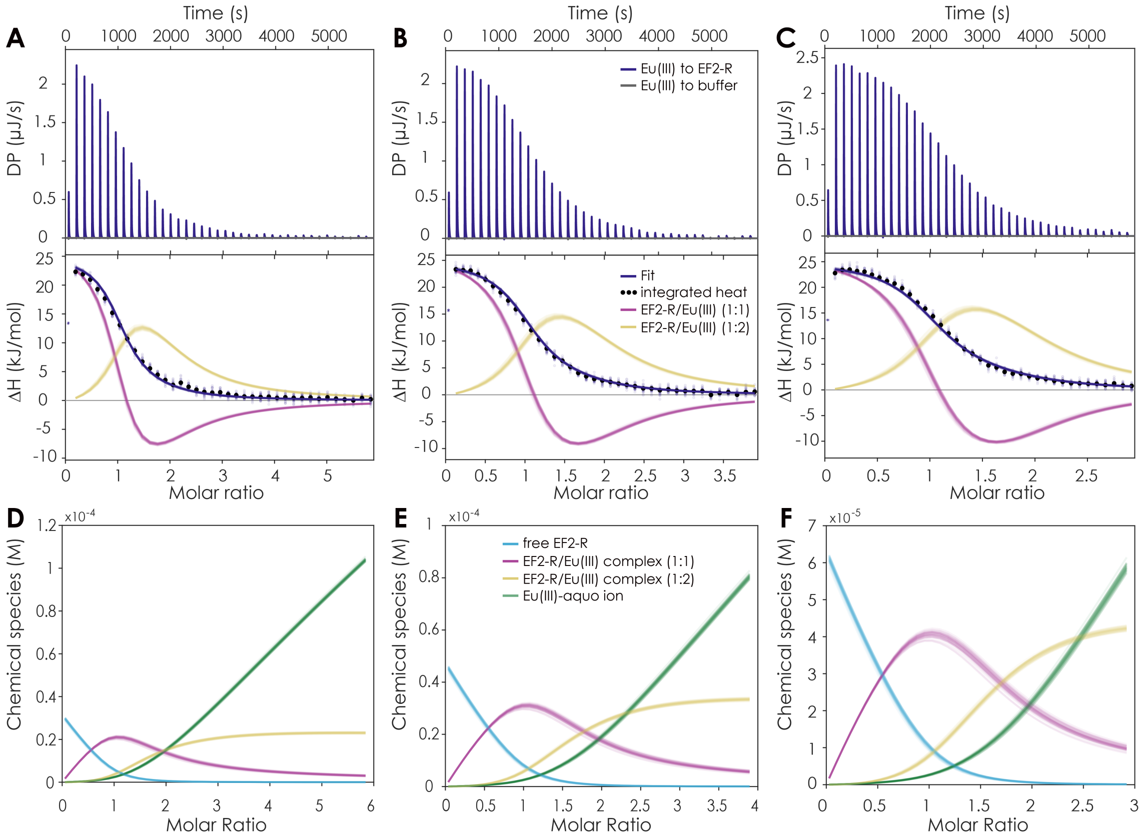

Figure S8 ITC data of EF2-R binding to Eu(III). Thermogram, integrated heat, and used fit with a two-component model obtained by titrating a 900 µM Eu(III) solution to **(A)** 30 µM peptide **(B)** 45 µM peptide and **(C)** 60 µM peptide. For the three independently obtained data sets a global fit was used; the first titration point was excluded from the analysis. Chemical species distribution observed in the ITC titration experiments **(D)** 900 µM Eu(III) to 30 µM peptide, **(E)** 900 µM Eu(III) to 45 µM peptide and **(F)** 60 µM peptide. Experimental conditions: pH 6.6, 25 °C, 10 mM MOPSO buffer, 100 mM KCl. Overlayed lines/ symbols represent 100 MC runs which were used for error estimation (see Section 3.2).

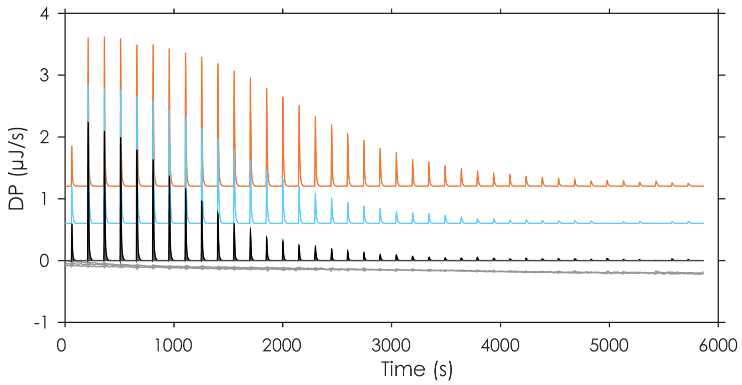

Figure S9: SVD-corrected thermograms of the Eu(III) to EF2-R titrations (orange: 60 µM set, blue: 45 µM set, black: 30 µM set) shown in Figure S8; the residuals for all three titrations are shown in grey. For better visualisation, the data was stacked with an offset to the x-axis.

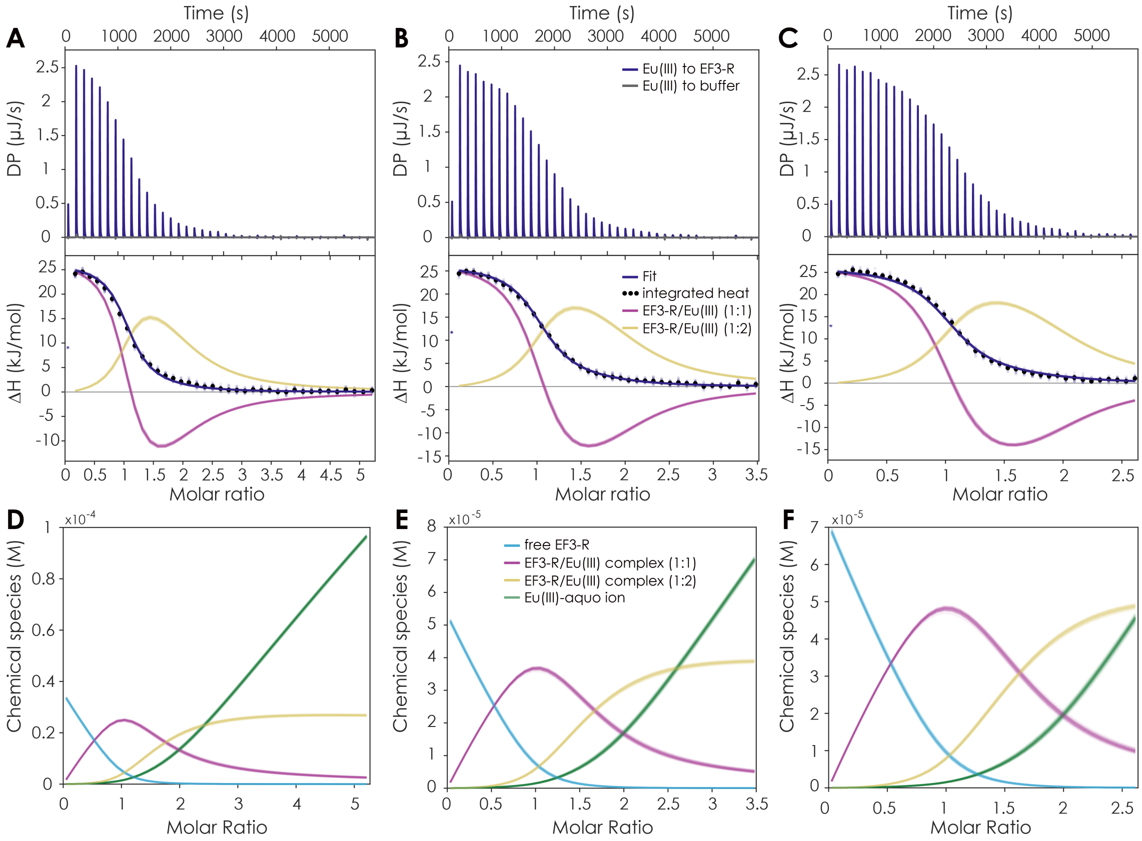

Figure S10 ITC data of EF3-R binding to Eu(III). Thermogram, integrated heat, and used fit with a two-component model obtained by titrating a 900 µM Eu(III) solution to **(A)** 30 µM peptide **(B)** 45 µM peptide and **(C)** 60 µM peptide. For the three independently obtained data sets a global fit was used; the first titration point was excluded from the analysis. Chemical species distribution observed in the ITC titration experiments **(D)** 900 µM Eu(III) to 30 µM peptide, **(E)** 900 µM Eu(III) to 45 µM peptide and **(F)** 60 µM peptide. Experimental conditions: pH 6.6, 25 °C, 10 mM MOPSO buffer, 100 mM KCl. Overlayed lines/ symbols represent 100 MC runs which were used for error estimation (see Section 3.2).

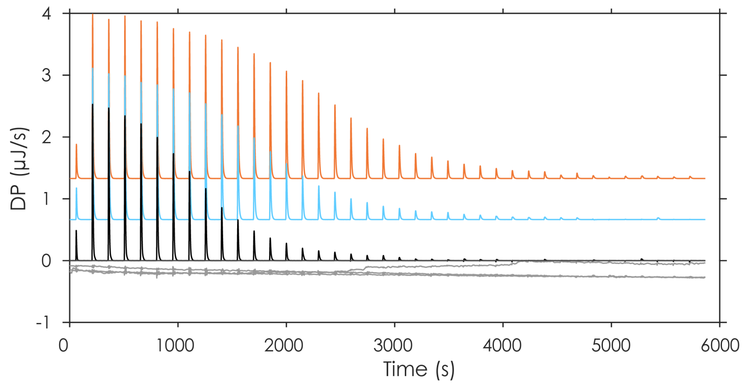

Figure S11 SVD-corrected thermograms of the Eu(III) to EF3-R titrations (orange: 60 µM set, blue: 45 µM set, black: 30 µM set) shown in Figure S10; the residuals for all three titrations are shown in grey. For better visualisation, the data was stacked with an offset to the x-axis.

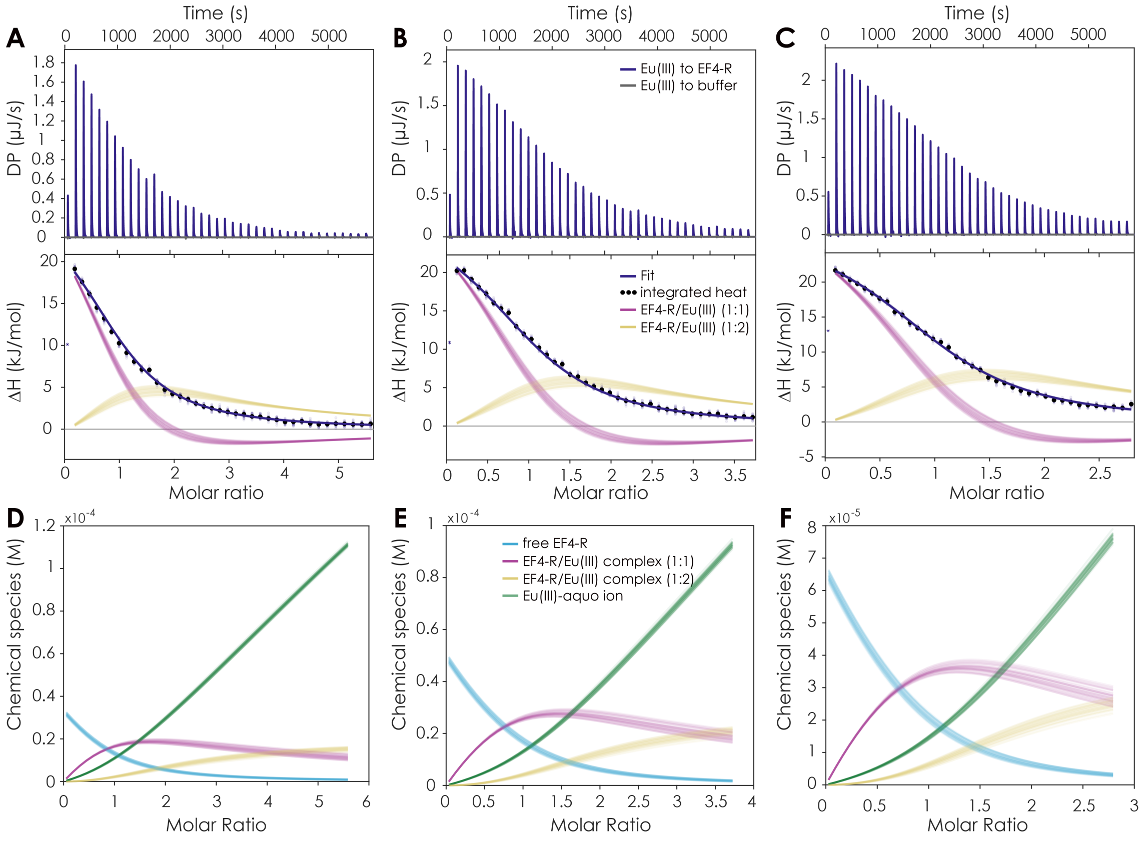

Figure S12 ITC data of EF4-R binding to Eu(III). Thermogram, integrated heat, and used fit with a two-component model obtained by titrating a 900 µM Eu(III) solution to **(A)** 30 µM peptide **(B)** 45 µM peptide and **(C)** 60 µM peptide. For the three independently obtained data sets a global fit was used; the first titration point was excluded from the analysis. Chemical species distribution observed in the ITC titration experiments **(D)** 900 µM Eu(III) to 30 µM peptide, **(E)** 900 µM Eu(III) to 45 µM peptide and **(F)** 60 µM peptide. Experimental conditions: pH 6.6, 25 °C, 10 mM MOPSO buffer, 100 mM KCl. Overlayed lines/ symbols represent 100 MC runs which were used for error estimation (see Section 3.2).

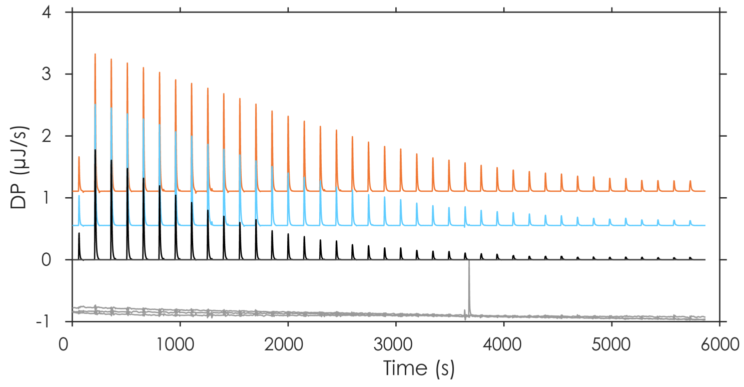

Figure S13 SVD-corrected thermograms of the Eu(III) to EF4-R titrations (orange: 60 µM set, blue: 45 µM set, black: 30 µM set) shown in Figure S12; the residuals for all three titrations are shown in grey. For better visualisation, the data was stacked with an offset to the x-axis.

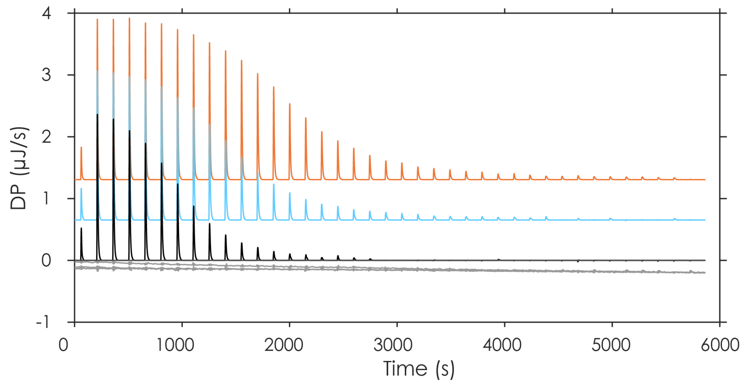

Figure S14 SVD-corrected thermograms of the Eu(III) to EF4-R_{mod} titrations (orange: 60 µM set, blue: 45 µM set, black: 30 µM set) shown in Figure 6; the residuals for all three titrations are shown in grey. For better visualisation, the data was stacked with an offset to the x-axis.

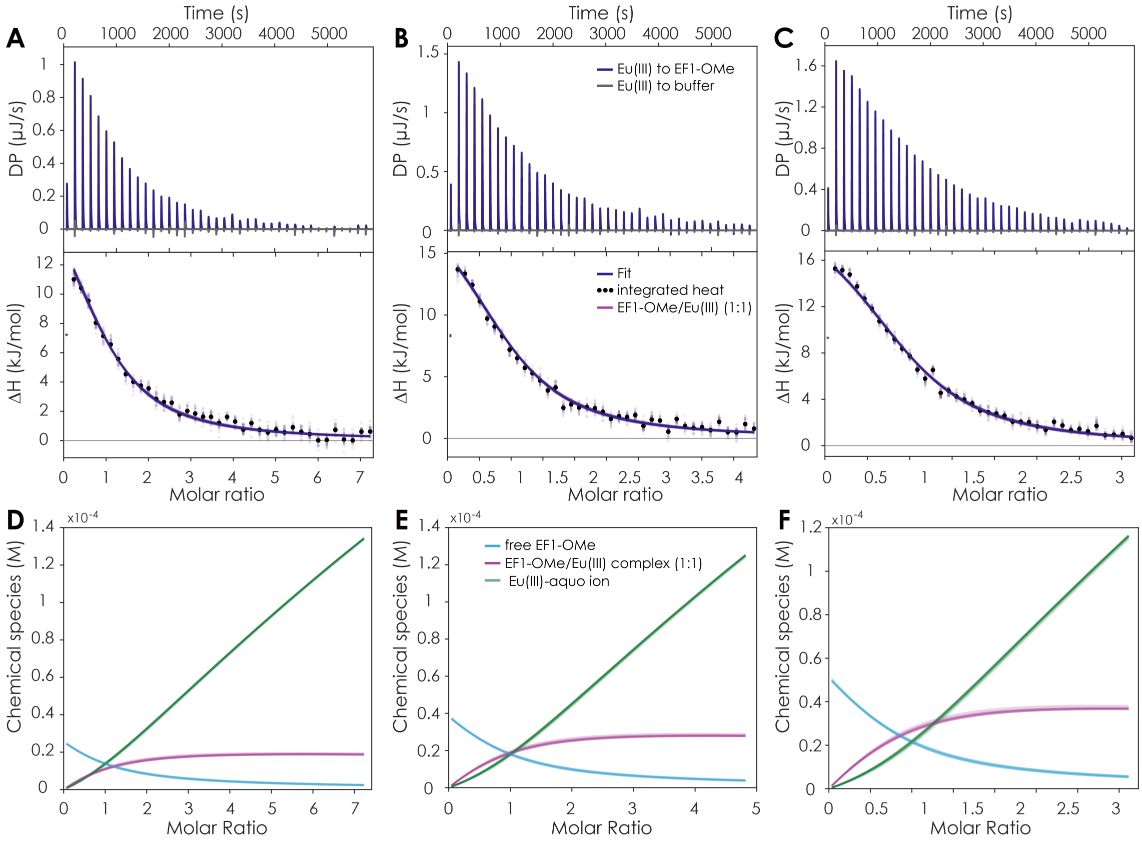

Figure S15 ITC data of EF1-OMe binding to Eu(III). Thermogram, integrated heat, and used fit with a two-component model obtained by titrating a 900 µM Eu(III) solution to **(A)** 30 µM peptide **(B)** 45 µM peptide and **(C)** 60 µM peptide. For the three independently obtained data sets a global fit was used; the first titration point was excluded from the analysis. Chemical species distribution observed in the ITC titration experiments **(D)** 900 µM Eu(III) to 30 µM peptide, **(E)** 900 µM Eu(III) to 45 µM peptide and **(F)** 60 µM peptide. Experimental conditions: pH 6.6, 25 °C, 10 mM MOPSO buffer, 100 mM KCl. Overlayed lines/ symbols represent 100 MC runs which were used for error estimation (see Section 3.2).

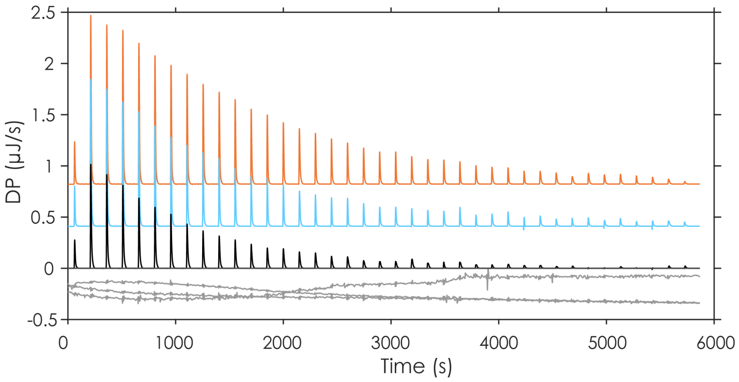

Figure S16 SVD-corrected thermograms of the Eu(III) to EF1-OMe titrations (orange: 60 µM set, blue: 45 µM set, black: 30 µM set) shown in Figure S15; the residuals for all three titrations are shown in grey. For better visualisation, the data was stacked with an offset to the x-axis.

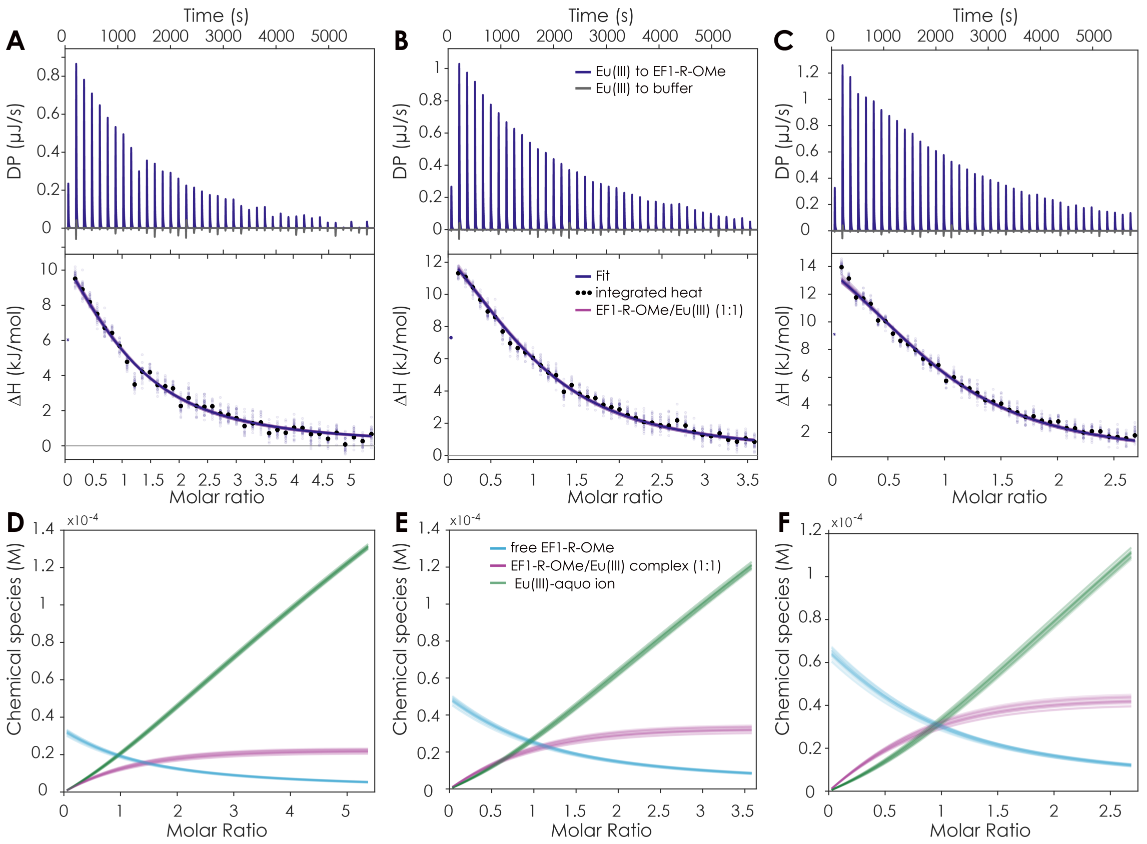

Figure S17 ITC data of EF1-R-OMe binding to Eu(III). Thermogram, integrated heat, and used fit with a two-component model obtained by titrating a 900 µM Eu(III) solution to **(A)** 30 µM peptide **(B)** 45 µM peptide and **(C)** 60 µM peptide. For the three independently obtained data sets a global fit was used; the first titration point was excluded from the analysis. Chemical species distribution observed in the ITC titration experiments **(D)** 900 µM Eu(III) to 30 µM peptide, **(E)** 900 µM Eu(III) to 45 µM peptide and **(F)** 60 µM peptide. Experimental conditions: pH 6.6, 25 °C, 10 mM MOPSO buffer, 100 mM KCl. Overlayed lines/ symbols represent 100 MC runs which were used for error estimation (see Section 3.2).

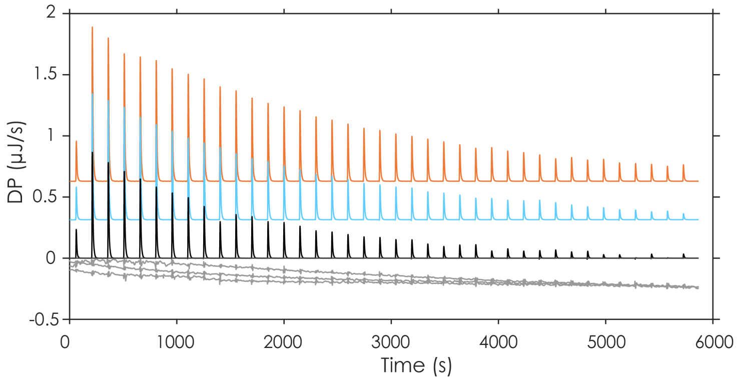

Figure S18 SVD-corrected thermograms of the Eu(III) to EF1-R-OMe titrations (orange: 60 µM set, blue: 45 µM set, black: 30 µM set) shown in Figure S17; the residuals for all three titrations are shown in grey. For better visualisation, the data was stacked with an offset to the x-axis.

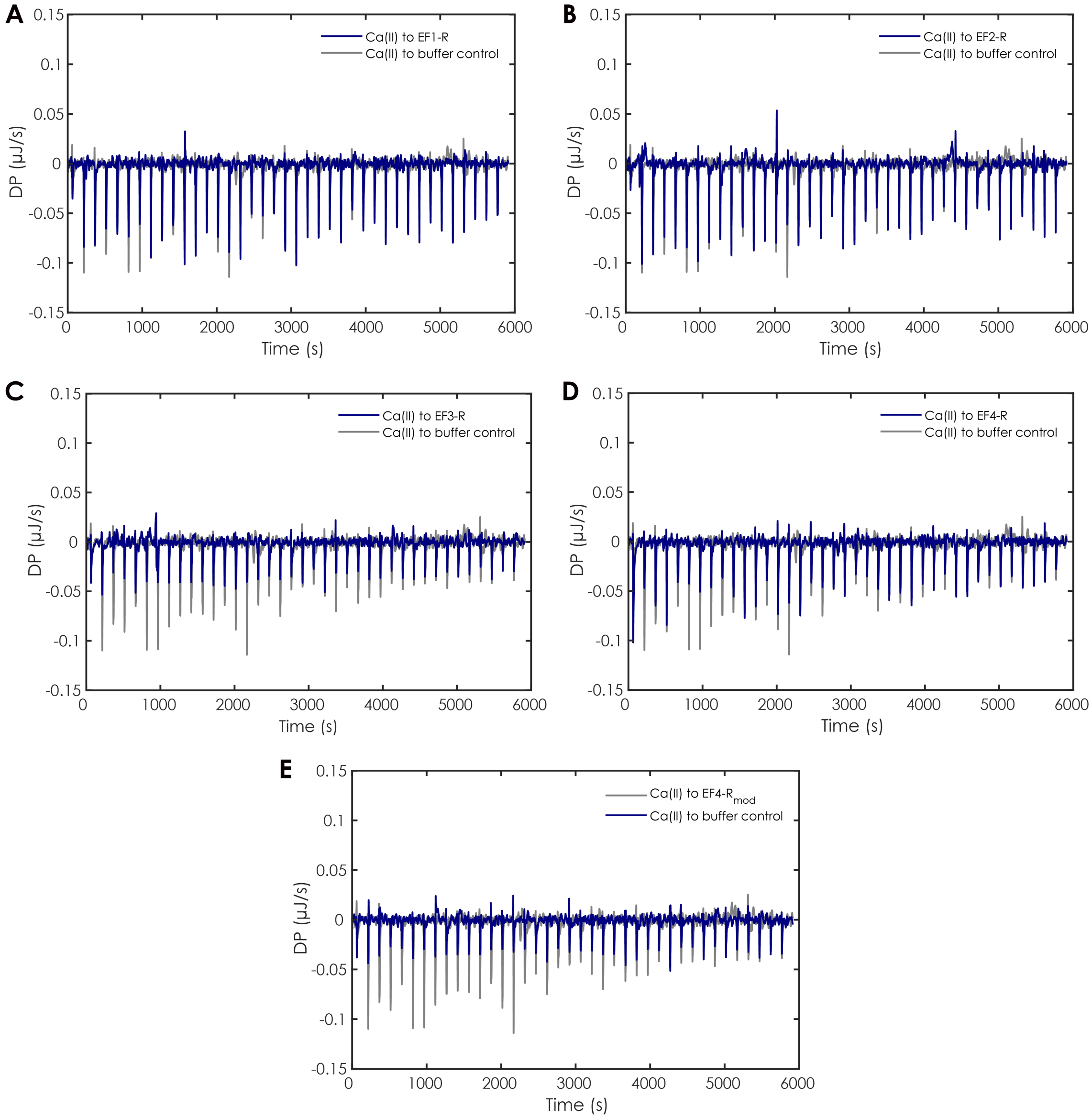

Figure S19 Baseline-corrected thermograms of a 9 mM Ca(II) to 30 µM **(A)** EF1-R and **(B)** EF2-R **(C)** EF3-R **(D)** EF4-R **(E)** EF4-R$_{mod}$ titration at pH 6.6 (10 mM MOPSO, 100 mM KCl); the corresponding background measurement ($CaCl_2$ to buffer) is shown in grey.

5.2 Supplementary TRLFS Data

Table S8 K_D values and luminescence lifetimes (**τ**) obtained from the combined-set EF to Eu(III)/ Eu(III) to EF TRLFS titration experiments.

	EF-Eu(III) 1:1 complex		EF-Eu(III) 1:2 complex		Eu(III)-aquo ion
	K_D (µM)	**τ** (µs)	K_D (µM)	**τ** (µs)	**τ** (µs)
**EF1**	4.4±0.3	189±7	49±6	135±4	111±0.9
**EF1-OMe**	9.5±0.5	185±4	-	-	110±0.4
**EF1-R-OMe**	8.6±0.9	151±1	-	-	110±0.4
**EF4-R_mod**	0.15±0.05	210±2	0.76±0.24	139±2	111±0.9

Table S9 Supplementary information to Table 1 in the main manuscript.

	Eu(III)-aquo ion **τ** (µs)
**EF1-R**	111±0.9
**EF2-R**	111±1.0
**EF3-R**	111±0.9
**EF4-R**	111±0.9

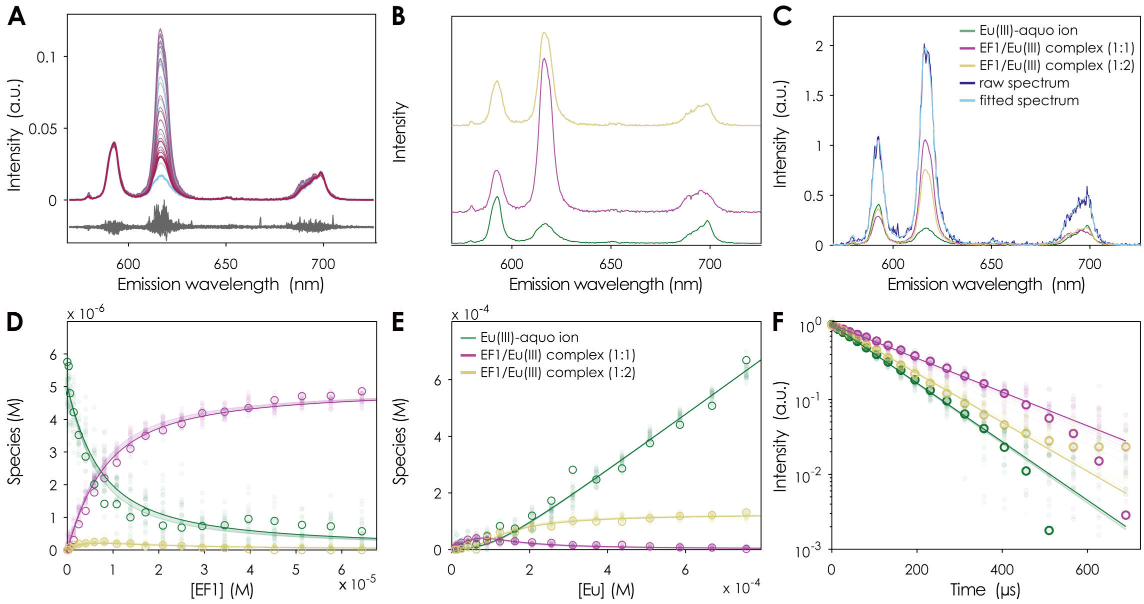

Figure S20 Combined EF1 to Eu(III) and Eu(III) to EF1 TRLFS titration experiment (10 mM MOPSO pH 6.6, 100 mM KCl), λ_{ex} (Eu) = 394 nm. **(A)** t_0 spectra of the PARAFAC model (blue to red with increasing peptide/Eu(III) complex concentration) normalised to the $^5D_0 \rightarrow {}^7F_1$ transition; residues in grey. **(B)** Stacked spectra of the three different species: Eu(III)-aquo ion, the 1:1 EF1 to Eu(III) complex and the 1:2 EF1 to Eu(III) complex. **(C)** Deconvoluted spectra of a representative step showing the spectrum of the Eu(III)-aquo ion, the 1:1 EF1 to Eu(III) complex and the 1:2 EF1 to Eu(III) complex. **(D)** Speciation of the first part of the titration experiment with increasing peptide concentration. **(E)** Speciation of the second part of the titration experiment with increasing Eu(III) concentration. **(F)** Lifetimes of the observed species. The colour code shown in E applies for B, D, and F as well. Shaded lines/ symbols represent MC runs used for error estimation (see Section 3.3).

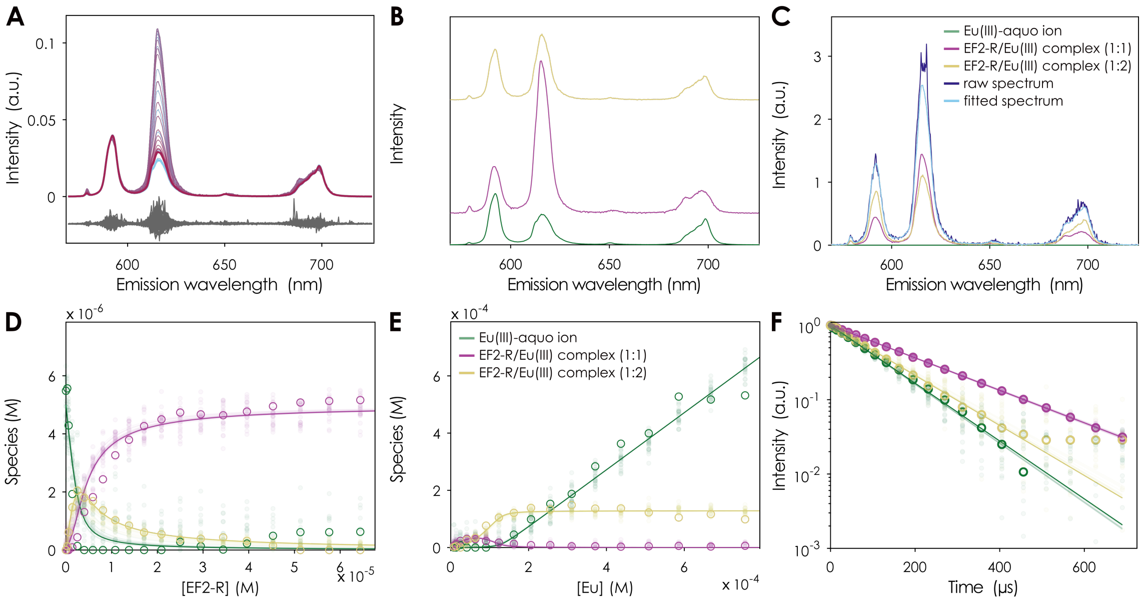

Figure S21 Combined EF2-R to Eu(III) and Eu(III) to EF2-R TRLFS titration experiment (10 mM MOPSO pH 6.6, 100 mM KCl), λ_{ex} (Eu) = 394 nm. **(A)** t_0 spectra of the PARAFAC model (blue to red with increasing peptide/Eu(III) complex concentration) normalised to the $^5D_0 \rightarrow {}^7F_1$ transition; removed noise shown in grey. **(B)** Stacked spectra of the three different species: Eu(III)-aquo ion, the 1:1 EF2-R to Eu(III) complex and the 1:2 EF2-R to Eu(III) complex. **(C)** Deconvoluted spectra of a representative step showing the spectrum of the Eu(III)-aquo ion, the 1:1 EF2-R to Eu(III) complex and the 1:2 EF2-R to Eu(III) complex. **(D)** Speciation of the first part of the titration experiment with increasing peptide concentration. **(E)** Speciation of the second part of the titration experiment with increasing Eu(III) concentration. **(F)** Lifetimes of the observed species. The colour code shown in E applies for B, D, and F; shaded lines/ symbols represent MC runs used for error estimation (see Section 3.3).

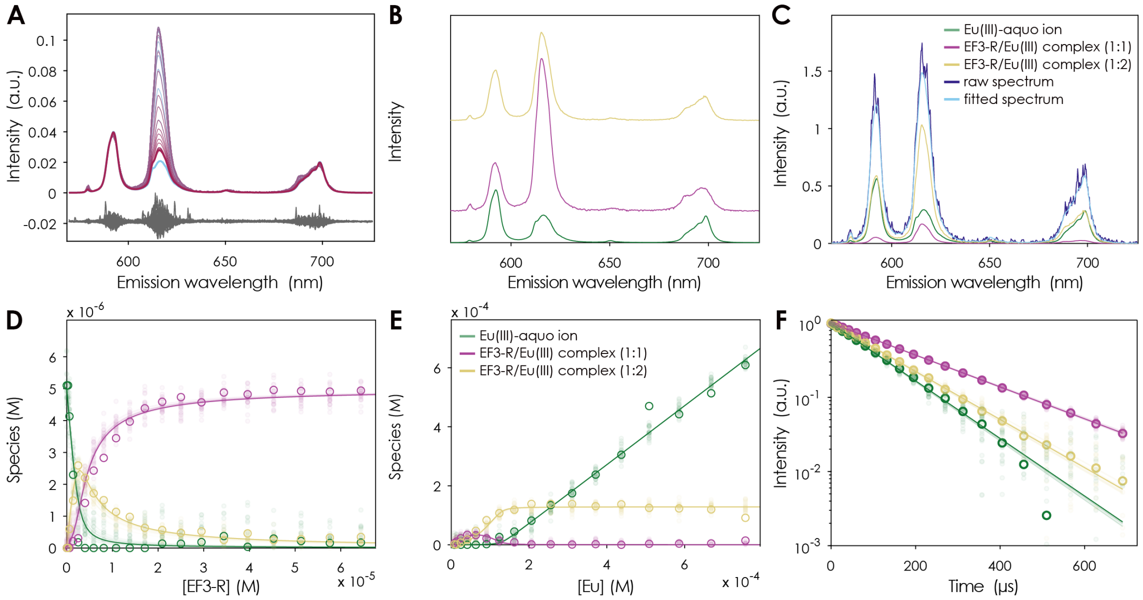

Figure S22 Combined EF3-R to Eu(III) and Eu(III) to EF3-R TRLFS titration experiment (10 mM MOPSO pH 6.6, 100 mM KCl), λ_{ex} (Eu) = 394 nm. **(A)** t_0 spectra (blue to red with increasing peptide/Eu(III) complex concentration) normalised to the $^5D_0 \rightarrow {}^7F_1$ transition; residues shown in grey. **(B)** Stacked spectra of the three different species: Eu(III)-aquo ion, the 1:1 EF3-R to Eu(III) complex and the 1:2 EF3-R to Eu(III) complex. **(C)** Deconvoluted spectra of a representative step showing the spectrum of the Eu(III)-aquo ion, the 1:1 EF3-R to Eu(III) complex and the 1:2 EF3-R to Eu(III) complex. **(D)** Speciation of the first part of the titration experiment with increasing peptide concentration. **(E)** Speciation of the second part of the titration experiment with increasing Eu(III) concentration. **(F)** Lifetimes of the observed species. The colour code shown in E applies for B, D, and F; shaded lines/ symbols represent MC runs used for error estimation (see Section 3.3).

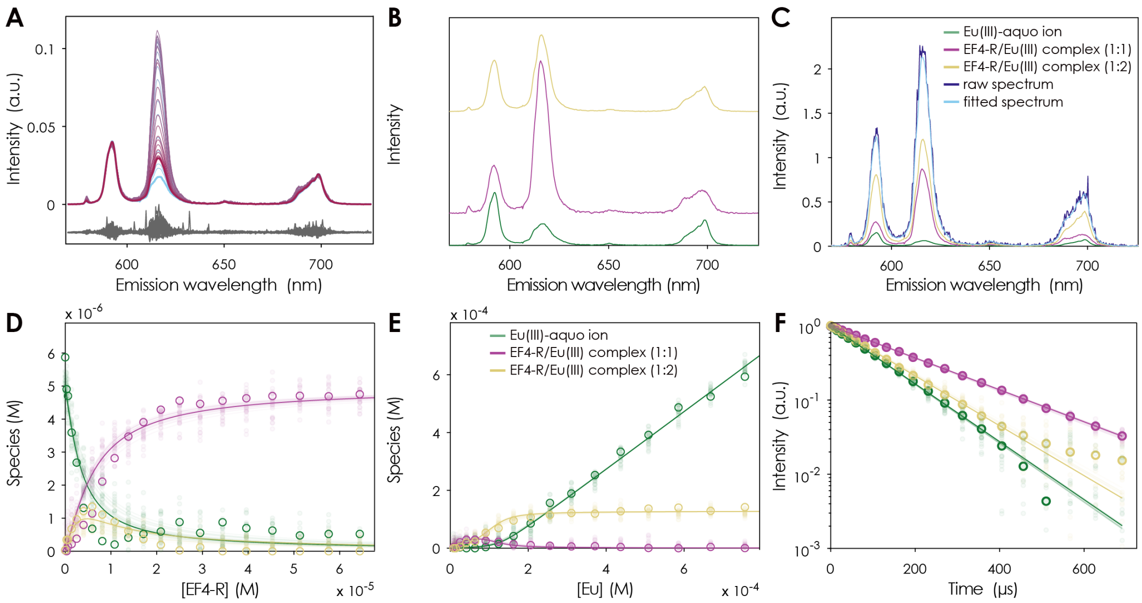

Figure S23 Combined EF4-R to Eu(III) and Eu(III) to EF4-R TRLFS titration experiment (10 mM MOPSO pH 6.6, 100 mM KCl), λ_{ex} (Eu) = 394 nm. **(A)** t_0 spectra of the PARAFAC model (blue to red with increasing peptide/Eu(III) complex concentration) normalised to the $^5D_0 \rightarrow {}^7F_1$ transition; residues shown in grey. **(B)** Stacked spectra of the three different species: Eu(III)-aquo ion, the 1:1 EF4-R to Eu(III) complex and the 1:2 EF4-R to Eu(III) complex. **(C)** Deconvoluted spectra of a representative step showing the spectrum of the Eu(III)-aquo ion, the 1:1 EF4-R to Eu(III) complex and the 1:2 EF4-R to Eu(III) complex. **(D)** Speciation of the first part of the titration experiment with increasing peptide concentration. **(E)** Speciation of the second part of the titration experiment with increasing Eu(III) concentration. **(F)** Lifetimes of the observed species. The colour code shown in E applies for B, D, and F; shaded lines/ symbols represent MC runs used for error estimation (see Section 3.3).

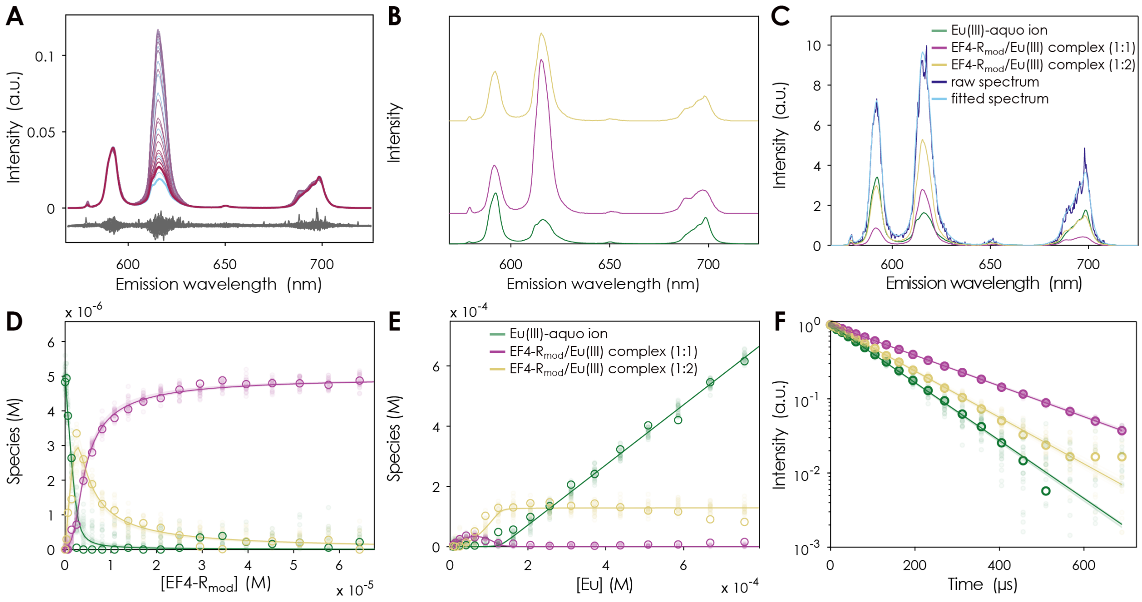

Figure S24 Combined EF4-R_{mod} to Eu(III) and Eu(III) to EF4-R_{mod} TRLFS titration experiment (10 mM MOPSO pH 6.6, 100 mM KCl), λ_{ex} (Eu) = 394 nm. **(A)** Modelled t_0 spectra (blue to red with increasing peptide/Eu(III) complex concentration) normalised to the $^5D_0 \rightarrow {}^7F_1$ transition; residues shown in grey. **(B)** Stacked spectra of the three different species: Eu(III)-aquo ion, the 1:1 EF4-R_{mod} to Eu(III) complex and the 1:2 EF4-R_{mod} to Eu(III) complex. **(C)** Deconvoluted spectra of a representative step showing the spectrum of the Eu(III)-aquo ion, the 1:1 EF4-R_{mod} to Eu(III) complex and the 1:2 EF4-R_{mod} to Eu(III) complex. **(D)** Speciation of the first part of the titration experiment with increasing peptide concentration. **(E)** Speciation of the second part of the titration experiment with increasing Eu(III) concentration. **(F)** Lifetimes of the observed species. The colour code shown in E applies for B, D, and F; shaded lines/ dots represent MC runs used for error estimation (see Section 3.3).

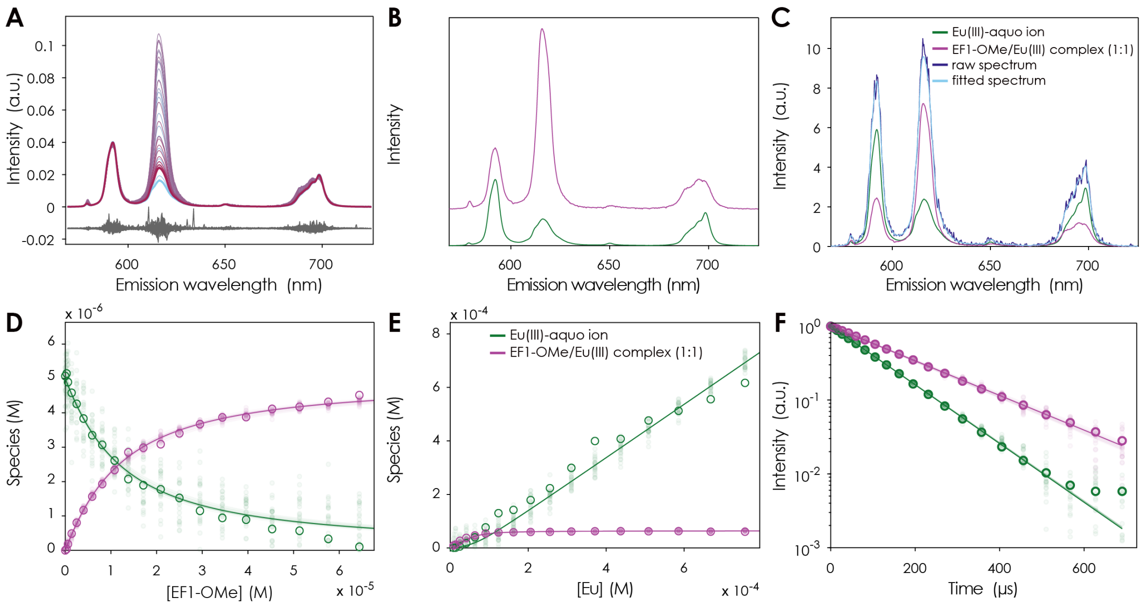

Figure S25 Combined EF1-OMe to Eu(III) and Eu(III) to EF1-OMe TRLFS titration experiment (10 mM MOPSO pH 6.6, 100 mM KCl), λ_{ex} (Eu) = 394 nm. **(A)** Modelled t_0 spectra (blue to red with increasing peptide/Eu(III) complex concentration) normalised to the $^5D_0 \rightarrow {}^7F_1$ transition; residues shown in grey. **(B)** Stacked spectra of the two different species: Eu(III)-aquo ion and the 1:1 EF1-OMe to Eu(III) complex. **(C)** Deconvoluted spectra of a representative step showing the spectrum of the Eu(III)-aquo ion and the 1:1 EF1-OMe to Eu(III) complex. **(D)** Speciation of the first part of the titration experiment with increasing peptide concentration. **(E)** Speciation of the second part of the titration experiment with increasing Eu(III) concentration. **(F)** Lifetimes of the observed species. The colour code shown in E applies for B, D, and F; shaded lines/ symbols represent MC runs used for error estimation (see Section 3.3).

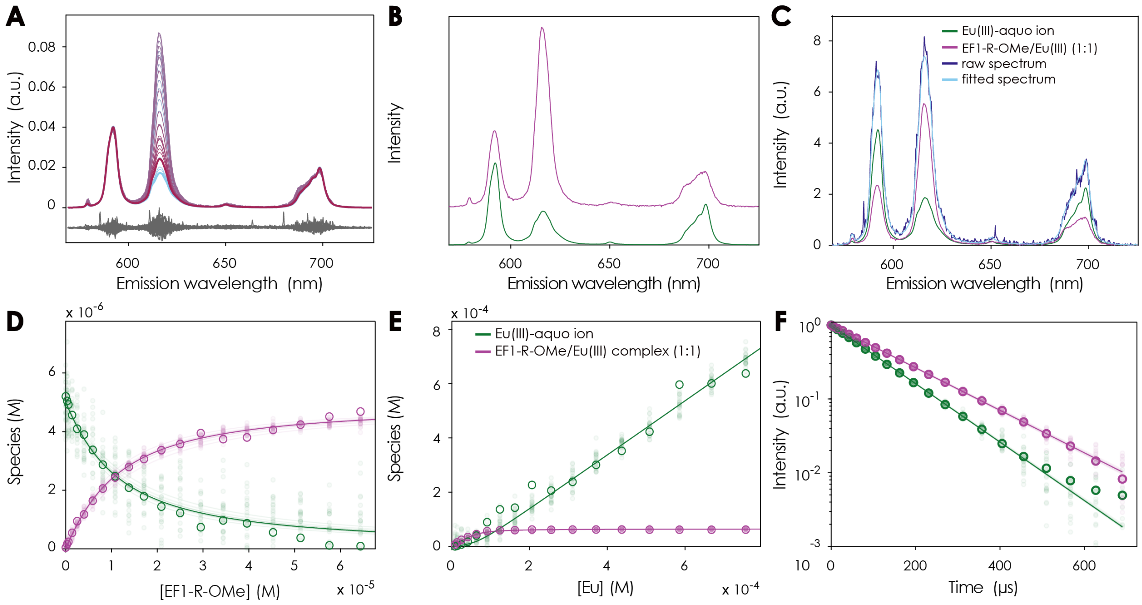

Figures S26 Combined EF1-R-OMe to Eu(III) and Eu(III) to EF1-R-OMe TRLFS titration experiment (10 mM MOPSO pH 6.6, 100 mM KCl), λ_{ex} (Eu) = 394 nm. **(A)** Modelled t_0 spectra (blue to red with increasing peptide/Eu(III) complex concentration) normalised to the $^5D_0 \rightarrow {}^7F_1$ transition; residues shown in grey. **(B)** Stacked spectra of the two different species: Eu(III)-aquo ion and the 1:1 EF1-R-OMe to Eu(III) complex. **(C)** Deconvoluted spectra of a representative step showing the spectrum of the Eu(III)-aquo ion and the 1:1 EF1-R-OMe to Eu(III) complex. **(D)** Speciation of the first part of the titration experiment with increasing peptide concentration. **(E)** Speciation of the second part of the titration experiment with increasing Eu(III) concentration. **(F)** Lifetimes of the observed species. The colour code in E applies for B, D, and F; shaded lines/ dots represent MC runs used for error estimation (see Section 3.3).

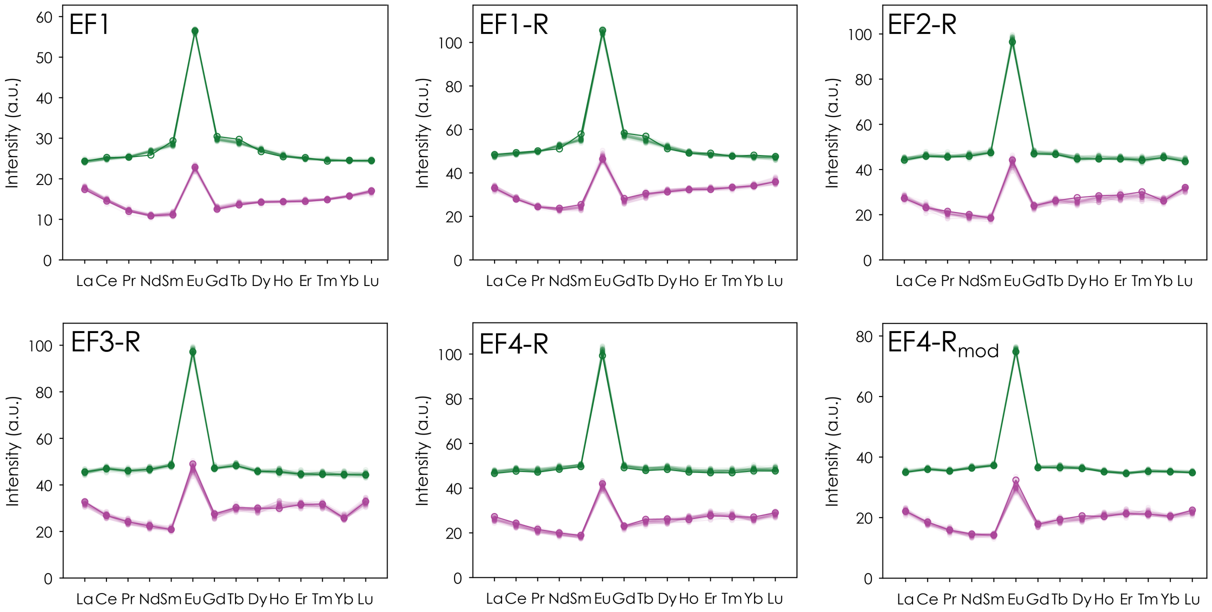

Figure S27 PARAFAC (solid line) and MC (transparent lines) distribution of the Ln-series for the peptides EF1, EF1-R, EF2-R, EF3-R, EF4-R, EF4-R$_{mod}$ showing the Eu-aquo ion and complexed Eu(III) (see Section 3.3.2). The ratio of these data is used to assess the overall affinity of the Ln as compared to Eu(III) (Figure 7). The colour code shown on the top right applies to all graphs.

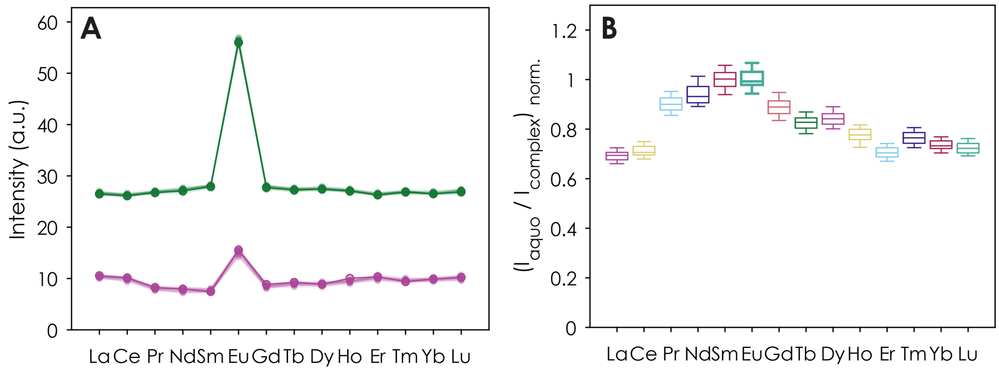

Figure S28 Lanthanide competition experiment between La(III)-Lu(III) (except Pm) *vs.* Eu(III) with EF1-OMe. **(A)** PARAFAC (solid line) and MC (transparent lines) distribution of the Ln-series for the peptide-complexed Eu(III) (magenta) and the free Eu(III)-aquo ion (green). **(B)** Relative affinities shown as box plot (median and quartiles).

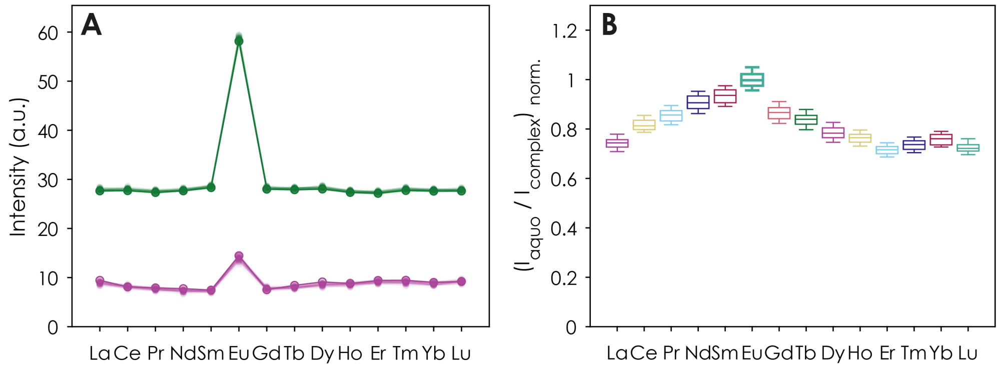

Figure S29 Lanthanide competition experiment between La(III)-Lu(III) (except Pm) *vs.* Eu(III) with EF1-R-OMe. **(A)** PARAFAC (solid line) and MC (transparent lines) distribution of the Ln-series for the peptide-complexed Eu(III) (magenta) and the free Eu(III)-aquo ion (green). **(B)** Relative affinities shown as box plot (median and quartiles).

5.3 Supplementary CD Data

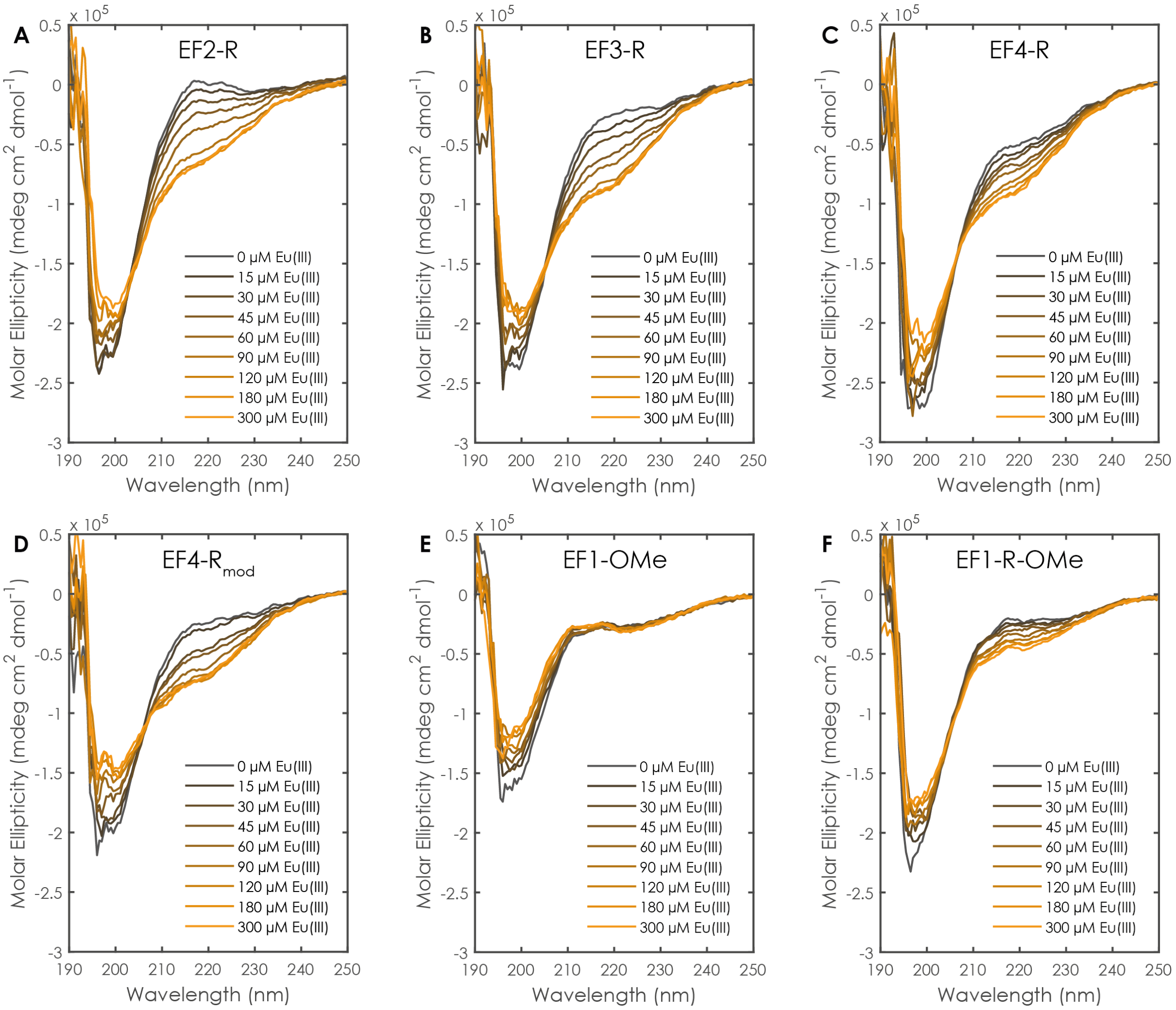

Figure S30 CD spectra of Eu(III) to peptide (60 µM) titration experiments at 25 °C and pH 6.6 (10 mM MOPSO, 100 mM KCl) **(A)** Eu(III) to EF2-R, **(B)** Eu(III) to EF3-R, **(C)** Eu(III) to EF4-R, **(D)** Eu(III) to EF4-R$_{mod}$, **(E)** Eu(III) to EF1-OMe and **(F)** Eu(III) to EF1-R-OMe.

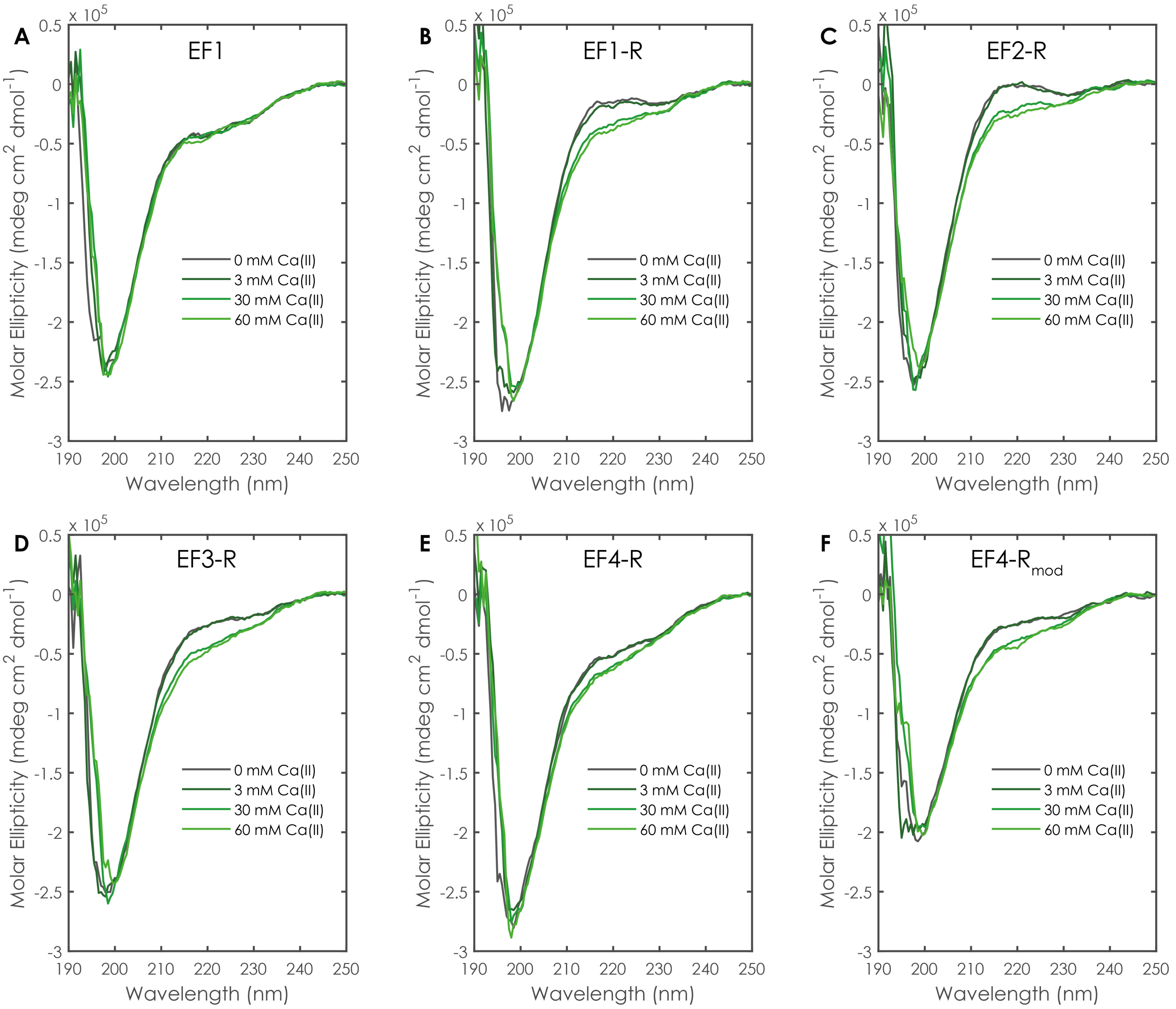

Figure S31 CD spectra of Ca(II) to peptide (60 µM) titration experiments at 25 °C and pH 6.6 (10 mM MOPSO, 100 mM KCl) **(A)** Ca(II) to EF1, **(B)** Ca(II) to EF1-R, **(C)** Ca(II) to EF2-R, **(D)** Ca(II) to EF3-R, **(E)** Ca(II) to EF4-R and **(F)** Ca(II) to EF4-R_{mod}.

5.4 Supplementary NMR Data

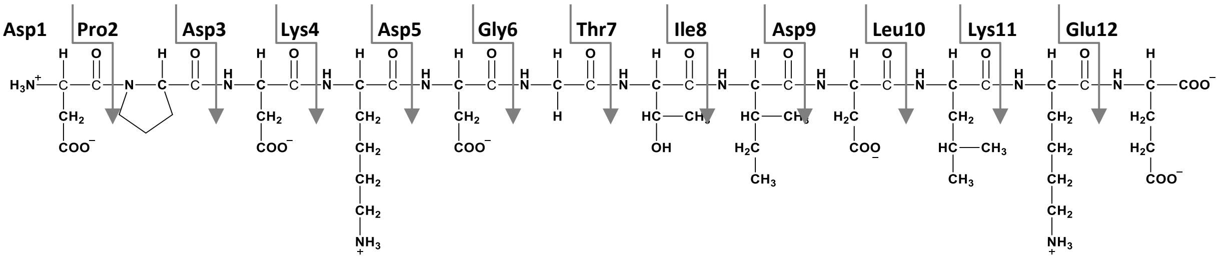

Figure S32 Structural view of the peptide EF1, separated by spin system.

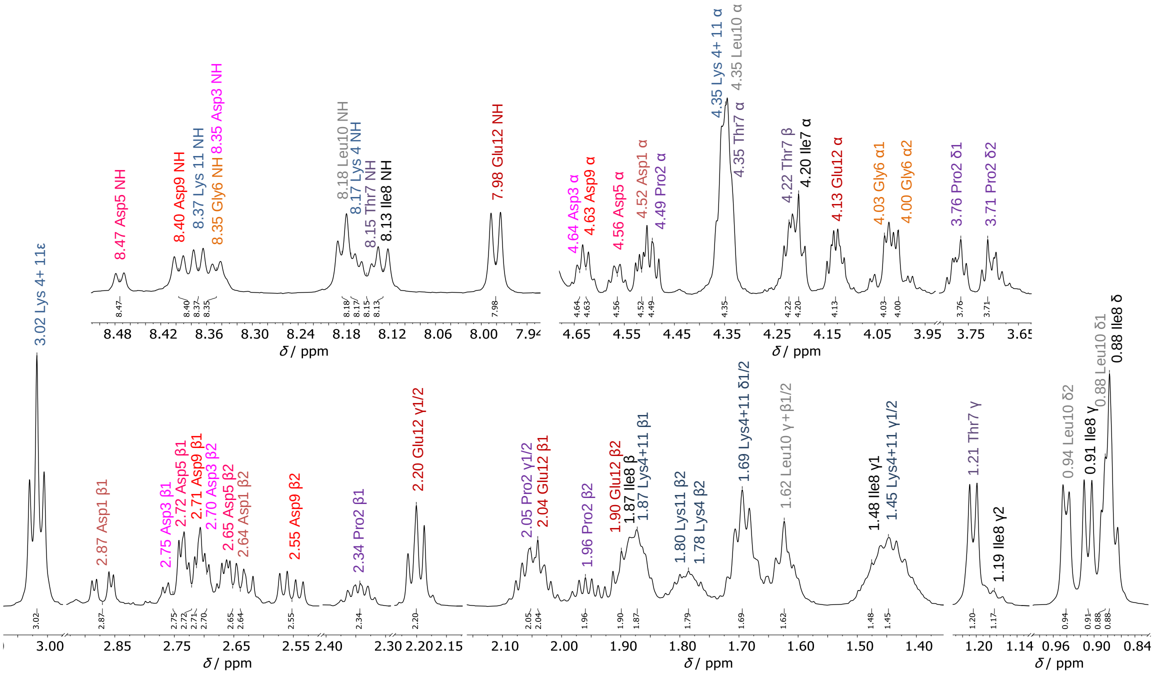

Figure S33 Overview and assignment of the ^{1}H NMR signals of EF1, colour-coded by amino acid (600 MHz, H_2O/D_2O (9:1) + 0.003% TMSP-d_4, 30 mM deuterated MES-d_{13}, 100 mM KCl).

Table S10 ^{1}H NMR chemical shifts of EF1 (600 MHz, H_2O/D_2O (9:1) + 0.003% TMSP-d_4, 30 mM MES-d_{13}, 100 mM KCl).

	NH	Hα	Hβ	Hγ	Hδ	Hε
**Asp1**	-	4.52	2.87,2.64	-	-	-
**Pro2**	-	4.49	2.34, 1.96	2.05	3.76, 3.71	-
**Asp3**	8.35	4.64	2.74, 2.70	-	-	-
**Lys4**	8.37	4.35	1.87, 1.78	1.45	1.69	3.02
**Asp5**	8.47	4.56	2.72, 2.67	-	-	-
**Gly6**	8.35	4.03, 4.00	-	-	-	-
**Thr7**	8.15	4.35	4.22	1.21	-	-
**Ile8**	8.13	4.20	1.87	0.91; 1.48, 1.19	0.88	-
**Asp9**	8.40	4.63	2.71, 2.55	-	-	-
**Leu10**	8.18	4.35	1.62	1.62	0.88, 0.94	-
**Lys11**	8.17	4.35	1.87, 1.80	1.45	1.69	3.02
**Glu12**	7.98	4.13	2.04, 1.90	2.20	-	-

Table S11 ^{13}C NMR chemical shifts of EF1 obtained from HSQC and HMBC 2D experiments (600 MHz, H_2O/D_2O (9:1) + 0.003% TMSP-d_4, 30 mM MES-d_{13}, 100 mM KCl).

	C=O	Cα	Cβ	Cγ	Cδ	Cε	COOH
**Asp1**	171.60	52.82	39.25	-	-	-	178.63
**Pro2**	176.66	63.78	32.23	27.48	50.87	-	-
**Asp3**	176.63	54.93	41.41	-	-	-	180.23
**Lys4**	176.21	56.32/ 56.63§	33.01/ 33.36§	24.67§	29.08§	42.27§	-
**Asp5**	176.98	54.79	41.24	-	-	-	180.32
**Gly6**	174.74	45.72	-	-	-	-	-
**Thr7**	174.81	62.42	70.04	21.75	-	-	-
**Ile8**	175.78	61.30	39.15	17.55; 27.38	13.28	-	-
**Asp9**	175.89	54.30	41.37	-	-	-	180.20
**Leu10**	177.25	55.26	42.56	27.04	23.69; 25.06	-	-
**Lys11**	175.74	56.32/56.63§	33.01/33.36§	24.67§	29.08§	42.27§	-
**Glu12**	-	58.17	31.35	36.81	-	-	181.20term;184.83

§ It was not possible to distinguish between Lys4 and Lys11.

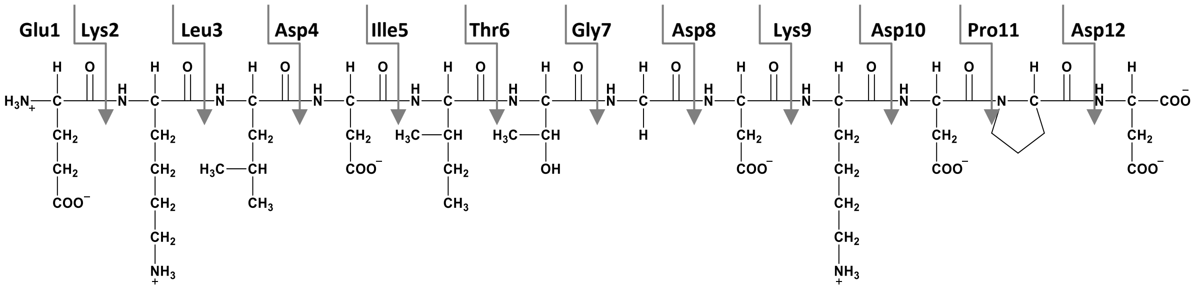

Figure S34 Structural view of EF1-R, separated by spin system.

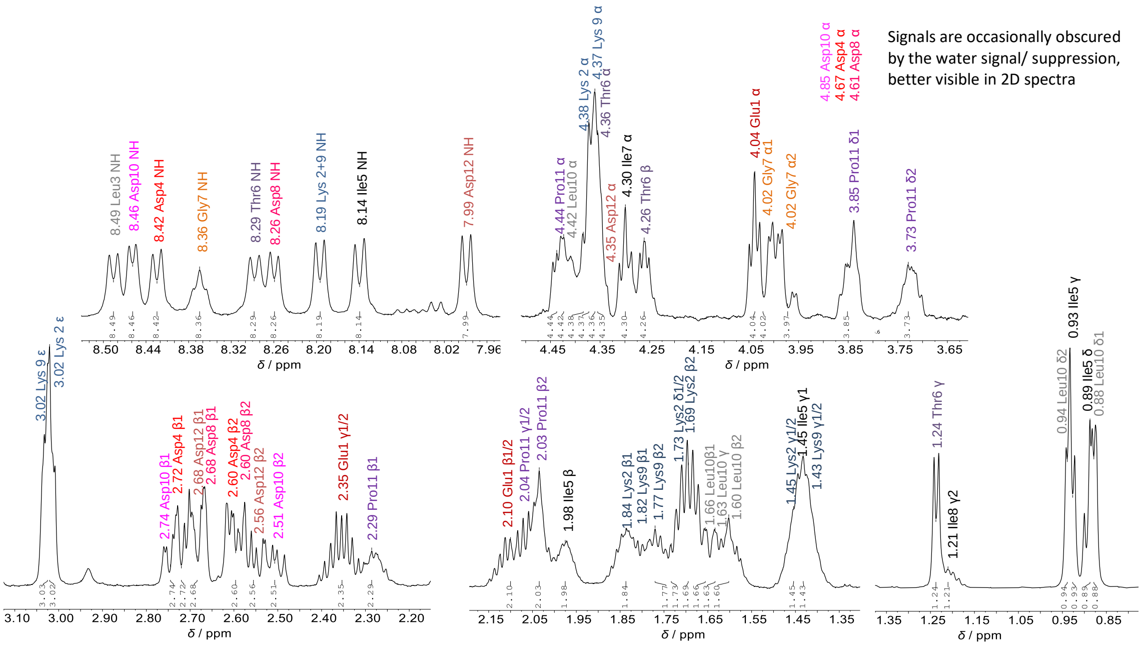

Figure S35 Overview and assignment of the ^{1}H NMR signals of EF1-R, colour-coded by amino acid (600 MHz, H_2O/D_2O (9:1) + 0.003% TMSP-d_4, 30 mM MES-d_{13}, 100 mM KCl).

Table S12 ^{1}H NMR chemical shifts of EF1-R (600 MHz, H_2O/D_2O (9:1) + 0.003% TMSP-d_4, 30 mM MES-d_{13}, 100 mM KCl).

	NH	Hα	Hβ	Hγ	Hδ	Hε
**Glu1**	-	4.04	2.10	2.35	-	-
**Lys2**	8.19	4.38	1.84, 1.69	1.45	1.73	3.02
**Leu3**	8.49	4.42	1.66, 1.60	1.63	0.88, 0.94	-
**Asp 4**	8.42	4.67	2.721, 2.598	-	-	-
**Ile5**	8.14	4.30	1.98	1.45, 1.21, 0.93	0.89	-
**Thr6**	8.29	4.36	4.26	1.24	-	-
**Gly7**	8.36	4.02, 3.97	-	-	-	-
**Asp8**	8.26	4.61	2.682, 2.593	-	-	-
**Lys9**	8.20	4.37	1.82, 1.77	1.43	1.69	3.03
**Asp 10**	8.46	4.85	2.74, 2.51			
**Pro11**	-	4.44	2.29, 2.03	2.04	3.85, 3.73	
**Asp12**	7.99	4.35	2.679, 2.556	-	-	-

Table S13 ^{13}C NMR chemical shifts of EF1-R obtained from HSQC and HMBC 2D experiments (600 MHz, H_2O/D_2O (9:1) + 0.003% TMSP-d_4, 30 mM MES-d_{13}, 100 mM KCl).

	C=O	Cα	Cβ	Cγ	Cδ	Cε	COOH
**Glu1**	172.87	55.65	30.57	35.89	-	-	183.32
**Lys2**	176.07	56.58	33.16	24.72	29.26	41.96	-
**Leu3**	176.99	55.09	42.80	27.13	23.59, 24.89	-	-
**Asp 4**	176.38	54.23	41.23	-	-	-	180.37
**Ile5**	176.84	61.32	38.91	27.21, 17.85	13.25	-	-
**Thr6**	175.36	62.29	69.85	21.61	-	-	-
**Gly7**	174.00	45.51	-	-	-	-	-
**Asp8**	176.15	54.40	41.41	-	-	-	180.37
**Lys9**	176.27	55.79	33.55	24.26	29.03	42.10	-
**Asp 10**	174.54	53.10	40.55				180.10;
**Pro11**	176.16	63.55	32.2	27.4	50.7	-	-
**Asp12**	-	56.16	42.24	-	-	-	$181.18^{term,}$ 181.64

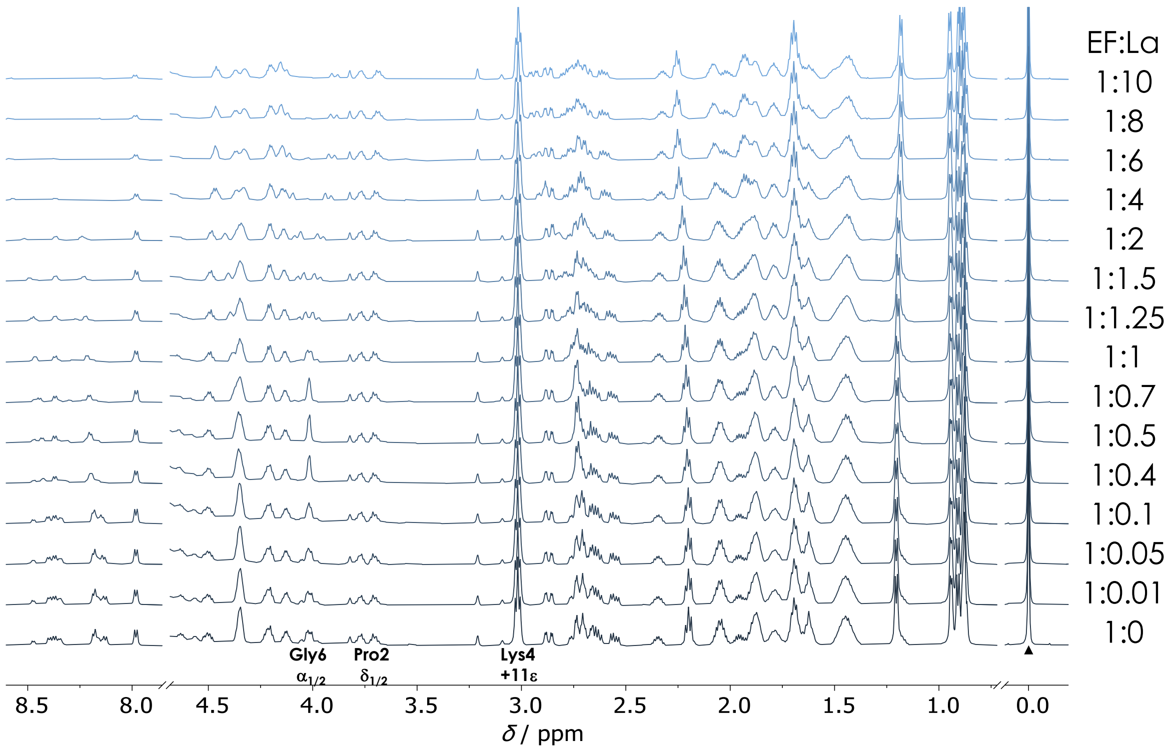

Figure S36 Titration of $LaCl_3$ to EF1 in increasing amounts (0 µM to 2 mM) while the peptide and buffer concentration was kept constant (600 MHz, H_2O/D_2O (9:1) + 0.003% TMSP-d_4, 30 mM MES-d_{13}, 100 mM KCl). The peptide to metal ratio for each step is shown next to the spectra. All spectra were referenced internally to the TMSP signal (black triangle). For most amino acid residues, the observed changes are rather small throughout the titration series. However, some key amino acids reveal well observable signatures: upon La(III)-addition all NH-associated signals exhibit displacements except the Glu12 NH signal, which remains unaltered throughout the series, indicating that the C-terminal carboxyl group is not involved in La(III)-binding. It is noteworthy that, among the Asp Hβ signals, only those of Asp3, Asp5, and Asp9 show significant shifts while those of Asp1 remain unaffected, supporting the MD calculations shown in Figure 4 of the main manuscript and in Table S16 showing no involvement of Asp1 in La(III)-binding. The AB spin system of the two Gly6 Hα features a distinctive shift pattern: up to a peptide to metal ratio of 1:0.7 the signals shift towards one another and show some broadening, but then, upon further increasing the La(III) concentration, separate with one shifting upfield and the other downfield, respectively indicating the regimes of predominating 1:1 and 1:2 complexes. Independent indications of an EF:La(III) species other than the 1:1 complex arise from TRLFS, ITC, and CD data.

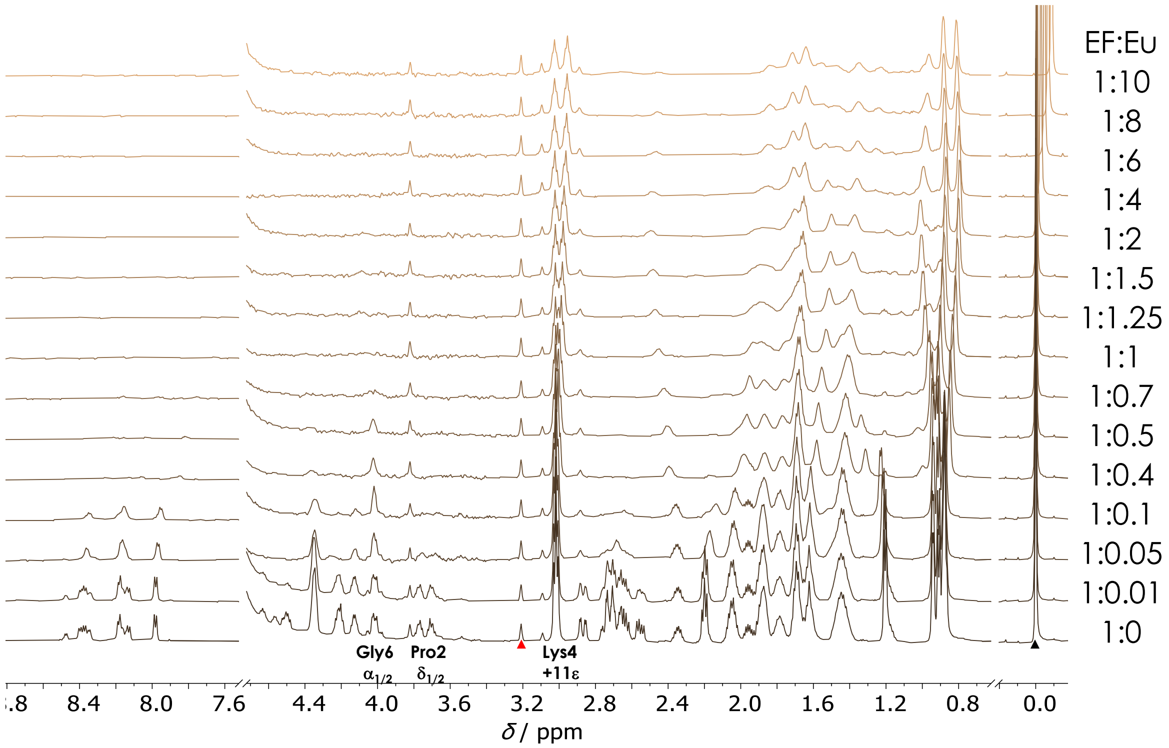

Figure S37 Titration of $EuCl_3$ to EF1 in increasing amounts (0 µM to 2 mM) while the peptide and buffer concentration was kept constant (600 MHz, H_2O/D_2O (9:1) + 0.003% TMSP-d_4, 30 mM MES-d_{13}, 100 mM KCl). The peptide to metal ratio for each step is shown next to the spectra. All spectra were referenced internally to the buffer signal at 3.21 ppm (red triangle) as the TMSP signal (black triangle) started to shift once the peptide was saturated with the paramagnetic metal. See Figure S39 for a control titration without peptide. In the Eu(III) to EF1 titration, within the first titration steps immediate peak broadening and, eventually, signal vanishing can be observed for all NH and Hα protons. The only Hα signals which can be well observed until a 1:0.5 peptide to metal ratio are those of Gly, showing analogous behaviour as observed for La(III), *i.e.* decreasing signal separation over the first titration steps and then again separating at higher metal concentration, even though the drift apart is poorly observable due to extreme signal broadening. Apart from the respective Hα signals, the other signals of the spin systems of Lys4 and Lys11, Ile8, and Leu10 remain well observable throughout the series, indicating again different shift trends for lower and higher metal to peptide ratios, pointing towards the transition between a 1:1 and a 1:2 complex. For Pro2, the Hβ and γ signals remain observable and the same phenomena of opposite shift trends within the series can be observed. Starting from a 1:4 peptide to metal ratio, the system seems to be saturated by Eu(III), as the TMSP signal starts to be affected by paramagnetic effects. In general, the severe signal broadening and vanishing is ascribed to the paramagnetism of Eu(III). The reason why only the Lys, Ile, and Leu side chain protons are much less affected – especially those most distant from the backbone – might be that they point away from the paramagnetic centre towards the surrounding medium as indicated by MD simulations in this work.

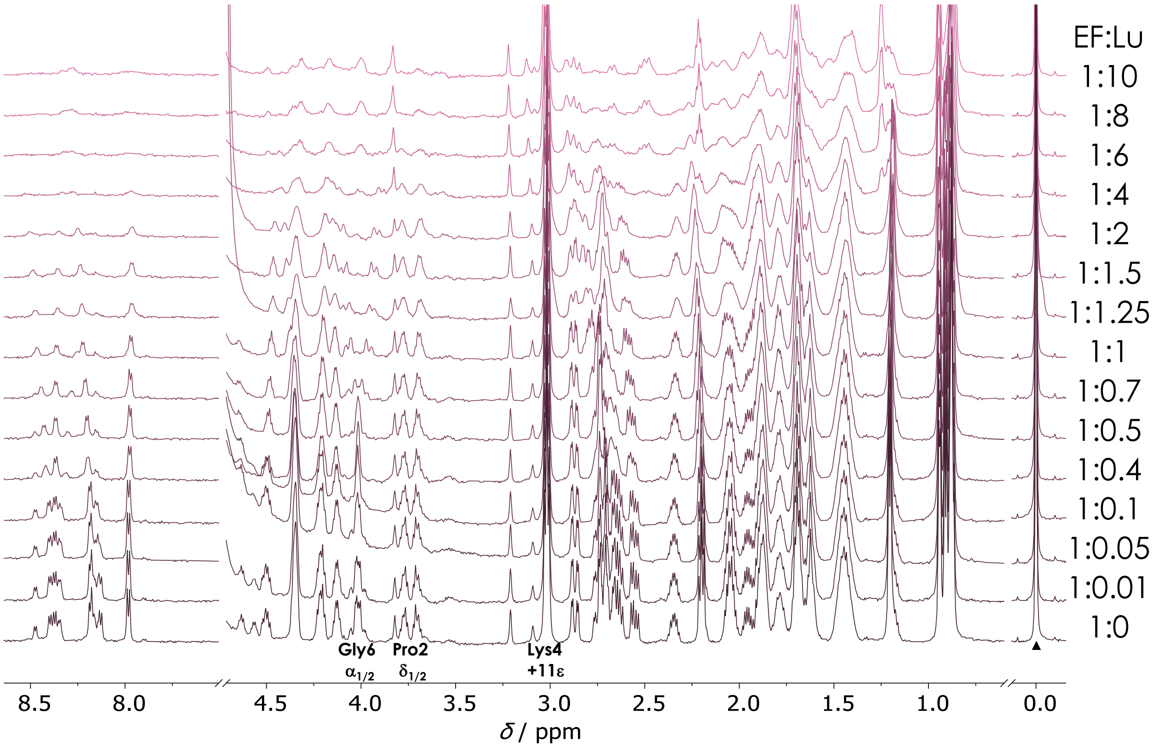

Figure S38 Titration of $LuCl_3$ to EF1 in increasing amounts (0 µM to 2 mM) while the peptide and buffer concentration was kept constant (600 MHz, H_2O/D_2O (9:1) + 0.003% TMSP-d_4, 30 mM MES-d_{13}, 100 mM KCl). The peptide to metal ratio for each step is shown next to the spectra. All spectra were referenced internally to the TMSP signal (black triangle). In the Lu(III) to EF1 titration set a clear difference in spectra of low and high metal to peptide ratios can be observed, again indicating the formation of not only a 1:1 but also a 1:2 complex (especially well observable for Thr7 γ, Ile8 γ2, Pro2 δ). As for the corresponding La(III) system, up to approximately equimolar EF1:Lu(III) ratio, from the invariance of Glu12's NH signal we infer that the C-terminal carboxyl group does not coordinate. As of metal excess, the significant broadening can possibly be attributed to a change in the speciation, *i.e.* the predominance of the 1:2 EF1:Lu(III) complex, mirroring some strain in the backbone chain upon complexation of a second metal. Especially, interesting shifts can be observed for Gly Hα, showing a behaviour similar to that of the La(III) and Eu(III) titration sets, *i.e.* bimodal, first showing the merging of the AB spin system, followed by successive and remarkable signal separation. This effect seems to happen at a lower metal to peptide ratio as for the La(III) series, potentially indicating either the stronger tendency of the smaller Lu(III) to form the 1:2 complex as compared to the larger La(III) or the lower stability of the Lu(III) 1:1 complex compared to that of La(III), hence being earlier replaced by the 1:2 species. Moreover, the much larger spectral effects observed for the formation of the presumed 1:2 complex between EF1 and Lu(III) compared to La(III) is ascribed to extensive structural changes necessary to accommodate the two smaller metal ions. Interestingly, as for La(III), Asp1 Hβ seems rather stable throughout the series indicating that, as suggested by MD simulations (Figure S80), in the 1:1 complex Asp1 is bent away from the metal. This seems to also apply for the 1:2 complex, suggesting some structural conservation between the 1:1 and 1:2 complex, even though the signals appear to be broader at high Lu(III) concentrations likely caused by association and dissociation dynamics among metal ion exchange reactions.

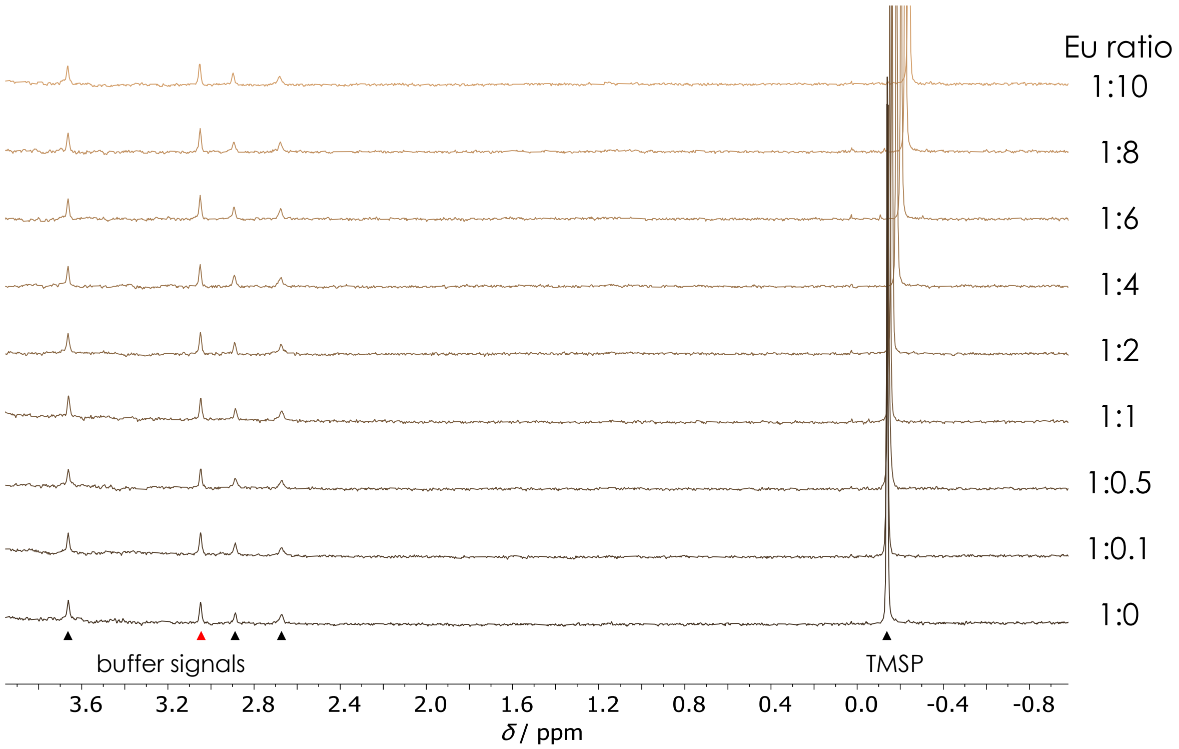

Figure S39 Unreferenced spectra of a $EuCl_3$ to buffer titration (600 MHz, H_2O/D_2O (9:1) + 0.003% TMSP-d_4, 30 mM MES-d_{13}, 100 mM KCl). The ratios stated with the spectra indicate the titration step in accordance with the $EuCl_3$ to peptide titrations. The interaction of the internal standard TMSP with the Eu(III) can clearly be seen, while the buffer's signals in the region 2.6 ppm to 3.8 ppm remain unshifted in the presence of the paramagnetic metal. Hence the $EuCl_3$ titration series were referenced to the buffer signal marked with a red triangle and not the internal standard TMSP.

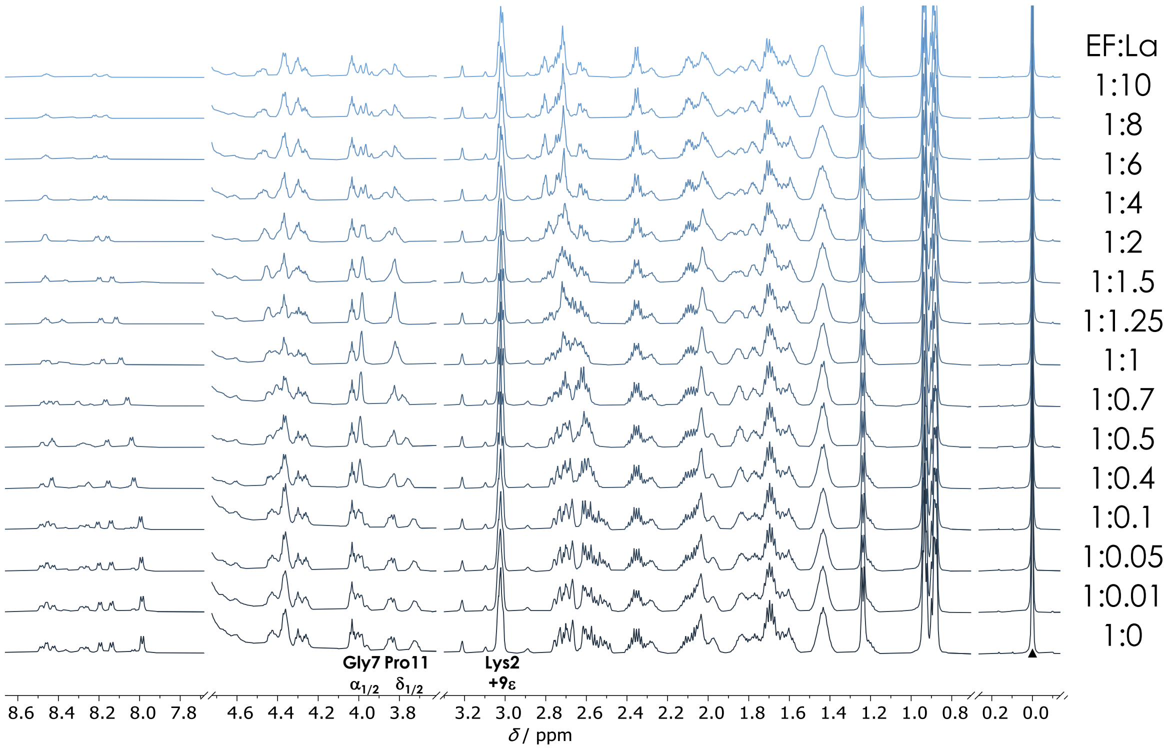

Figure S40 Titration of $LaCl_3$ to EF1-R in increasing amounts (0 µM to 2 mM) while the peptide and buffer concentration was kept constant (600 MHz, H_2O/D_2O (9:1) + 0.003% TMSP-d_4, 30 mM MES-d_{13}, 100 mM KCl). The peptide to metal ratio for each step is shown next to the spectra. All spectra were referenced internally to the TMSP signal (black triangle). Besides the peculiar behaviour of Asp12's NH as well as Pro11's Hδ signals, as described in the main document, further spectral changes along the titration series are worth mentioning. For instance, the NH signals associated with Lys2 and Lys9 exhibit only minor displacements during the first three metal addition steps, *i.e.* for predominance of the unbound peptide. As of significant 1:1 complex concentration (~1:0.4 EF:La(III) ratio) these signals suddenly shift significantly downfield, indicating substantial structural alterations arising from the transition from non-structured random-coil peptide to the 1:1 metal complex. In addition to backbone conformational changes, likely also some considerable alterations in the hydrogen bond network occur. Furthermore, the Gly Hα signals again display the same bimodal behaviour, as discussed earlier, but with the second "mode", *i.e.* the signal separation associated with formation of the 1:2 complex, occurring in later titration steps (higher La(III) molar excess) as compared to EF1. We attribute this observation to the one order of magnitude higher stability of EF1-R's 1:1 complex, causing the replacement of the former, *i*.e. the subsequent formation of the corresponding 1:2 complex, to require larger metal excess.

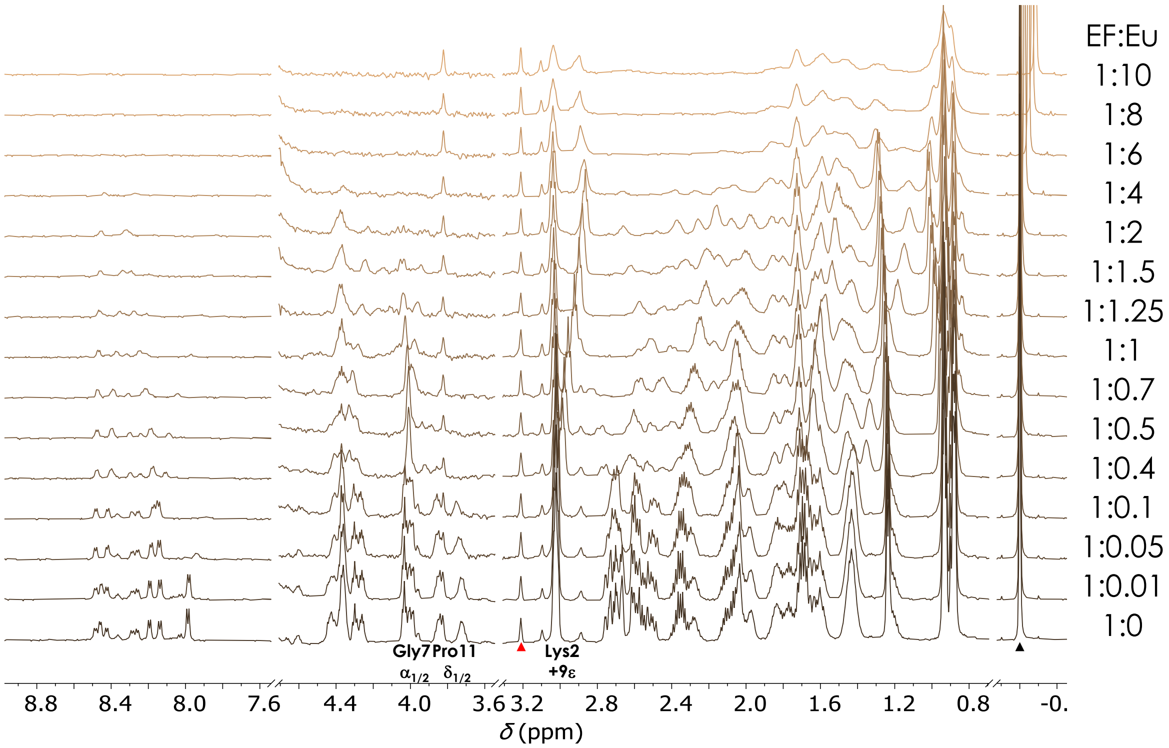

Figure S41 Titration of $EuCl_3$ to EF1-R in increasing amounts (0 µM to 2 mM) while the peptide and buffer concentration was kept constant (600 MHz, H_2O/D_2O (9:1) + 0.003% TMSP-d_4, 30 mM MES-d_{13}, 100 mM KCl). The peptide to metal ratio for each step is shown next to the spectra. All spectra were referenced internally to the buffer signal at 3.21 ppm (red triangle) as the TMSP signal (black triangle) started to shift once the peptide was saturated with the paramagnetic metal. See Figure S39 for a control titration without peptide. Upon comparison of the overall appearance of the two spectral sets obtained from the Eu(III) titration series with EF1 and EF1-R, apart from the few signals being observable throughout the series, obviously, the signals in general disappear much earlier for EF1 than for EF1-R. That is, in case of EF1 all NH and all Hα signals (except those of Gly6) as well as most of the Hβ signals disappear as of exceeding EF1:Eu(III) ratio of 1:0.1. In case of EF1-R, however, a much larger set of signals remains observable up to EF1-R:Eu(III) ratio of 1:2 while a subset of signals has disappeared already at EF1:Eu(III) ratio of 1:0.4. We attribute this distinguishing behaviour to two basic phenomena: vanishing of signals in consequence of extreme line broadening caused by (association and dissociation) dynamics as well as paramagnetic enhanced relaxation. The Eu(III) 1:1 complex of EF1 was shown by ITC and TRLFS to be thermodynamically less stable than that of EF1-R. Since practically all signals (except some due to peripheral protons such as Lys Hε, Ile and Leu methyl groups) disappear simultaneously as soon as the Eu(III) complex is present in substantial amounts, this complex appears to be also less stable in terms of kinetics, with NMR spectra suffering from broadening due to both above described reasons. On the contrary, those EF1-R signals disappearing early refer to sites close to the Eu(III) in the 1:1 complex sensing strong paramagnetic effects. Asp12's NH signal vanishes almost immediately (within the first two steps), as this residue is prone to interact strongly with Eu(III) as both the C-terminal and the sidechain carboxyl groups are involved as suggested by MD simulations (Figure 3). Interestingly, a Hε signal from one of the two lysines seems to indicate the transition from the 1:1 to the 1:2 complex very well, although being remote from the Eu(III). It starts to shift strongly upfield at the same titration point where the signals of Gly7 Hα start to separate again. At a 1:4 peptide to metal ratio, the system seems to be saturated as the excess Eu(III) starts to interact with the present TMSP.

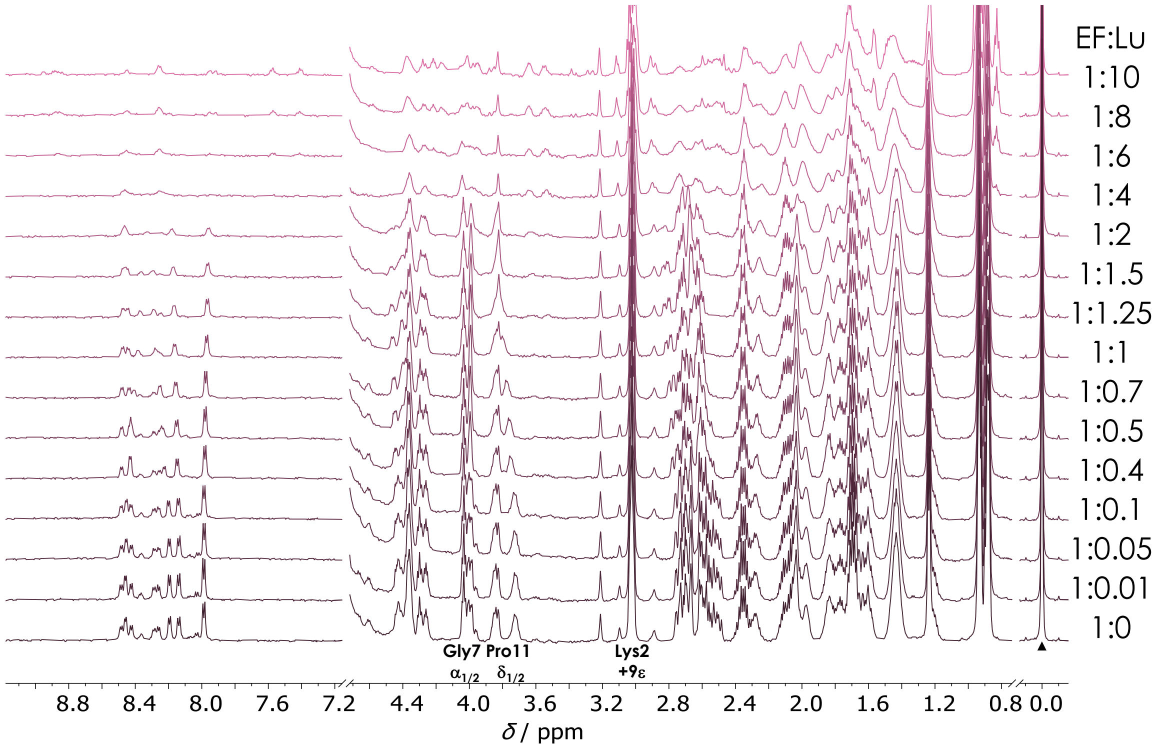

Figure S42 Titration of $LuCl_3$ to EF1-R in increasing amounts (0 µM to 2 mM) while the peptide and buffer concentration was kept constant (600 MHz, H_2O/D_2O (9:1) + 0.003% TMSP-d_4, 30 mM MES-d_{13}, 100 mM KCl). The peptide to metal ratio for each step is shown next to the spectra. All spectra were referenced internally to the TMSP signal (black triangle). Up to a EF:Lu(III) ratio of 1:2, the spectra reveal successive alterations as the fraction of the free peptide decreases and the fraction of EF1-R peptide bound as 1:1 Lu(III) complex increases. Note that selected spectral regions of that titration series are magnified in Figure S43.

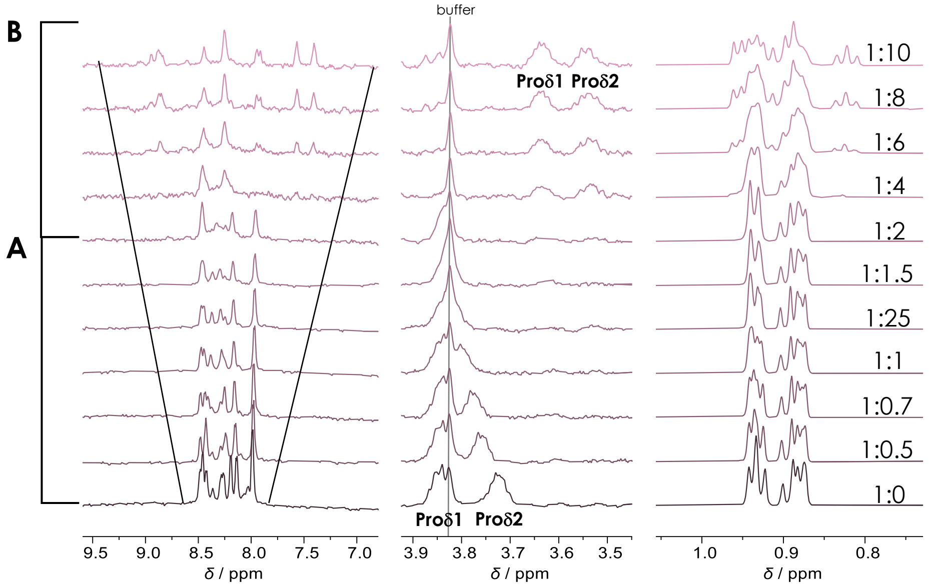

Figure S43 ^{1}H NMR spectral regions showing the NH signals (left) as well as signals of Pro11δ (middle) and of the methyl groups of Ile5 and Leu10 (right) for the Lu(III) to EF1-R titration series shown in Figure S42. The number of depicted titration steps was reduced to increase the signal traceability. Part **A** refers to the formation of the 1:1 complex related to comparably small spectral changes. Part **B** displays the transition from the 1:1 to the 1:2 complex as inferred from the newly emerging set of signals at a 1:4 ratio. Especially the signals due to Pro Hδ, Ile Hδ, and the Asps' NH signals. Of the latter, particularly those of Asp4 and Asp12 exhibit notable signal displacements downfield (8.95 ppm) and upfield (7.40 ppm), respectively, reflecting the substantial structural changes caused by the second Lu(III) ion. ^{1}H NMR signal assignment of the 1:2 complex is listed in Table S14**.**

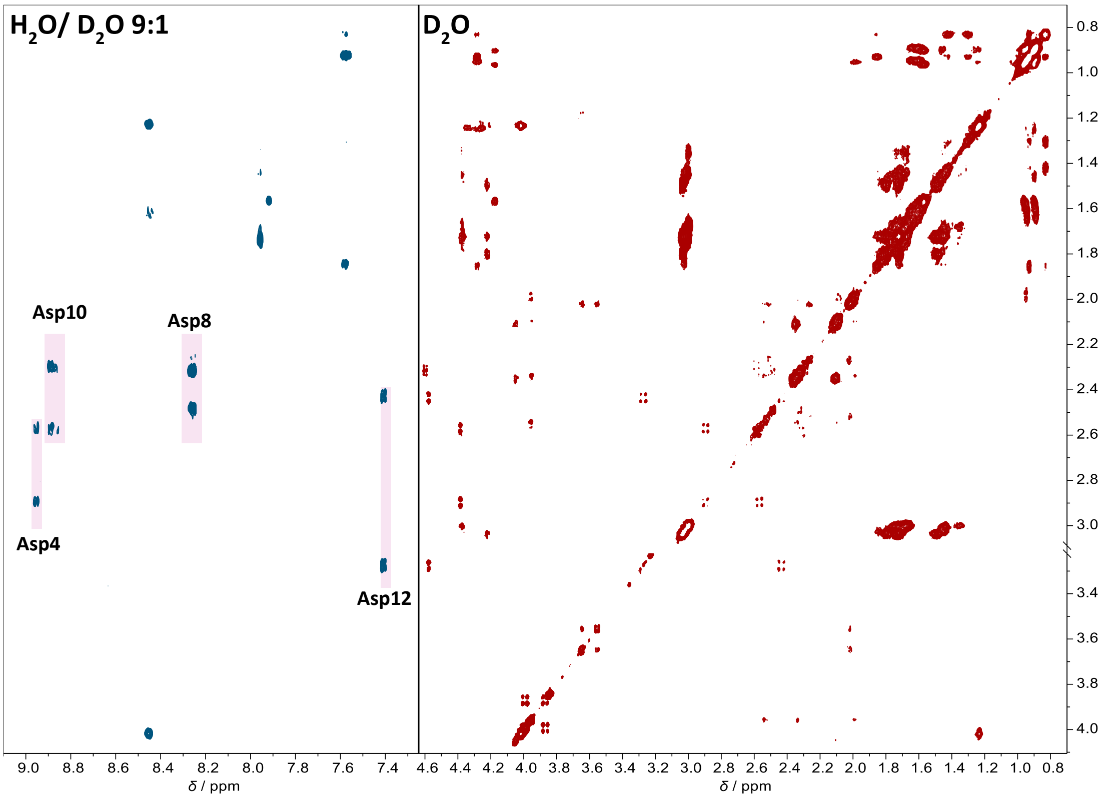

Figure S44 TOCSY spectra of the $LuCl_3$ to EF1-R titration series endpoint sample (EF:Lu(III) ratio = 1:10). The depicted NH region (left, blue signals) corresponds to the sample measured in D_2O/H_2O (9:1, + 0.003% TMSP-*d*$_4$, 30 mM MES-*d*$_{13}$, 100 mM KCl, pH 6.6) and the TOCSY showing the CH region (right, red signals) was obtained after lyophilisation and redissolving the sample in D_2O maintaining the same volume. The spectrum measured in H_2O/D_2O nicely correlates with the spectrum in D_2O, showing that the sample composition was maintained and a potential pH drift due to isotopic effects in D_2O (pH *vs.* pD) has no effect on the signal position because of the buffer capacity as wells as the fact that even for pH changes as high as ±0.5 units the peptide's protonation state remains unaltered. This combination of complementary spectra was used for the signal assignment of the 1:2 complex. The assigned chemical shifts are shown in Table S14. The signals of the Asps' spin systems are highlighted in the NH region. An excerpt of the corresponding TOCSY, proving the assignment of Asps' Hα along with Pro11α is shown in Figure S45.

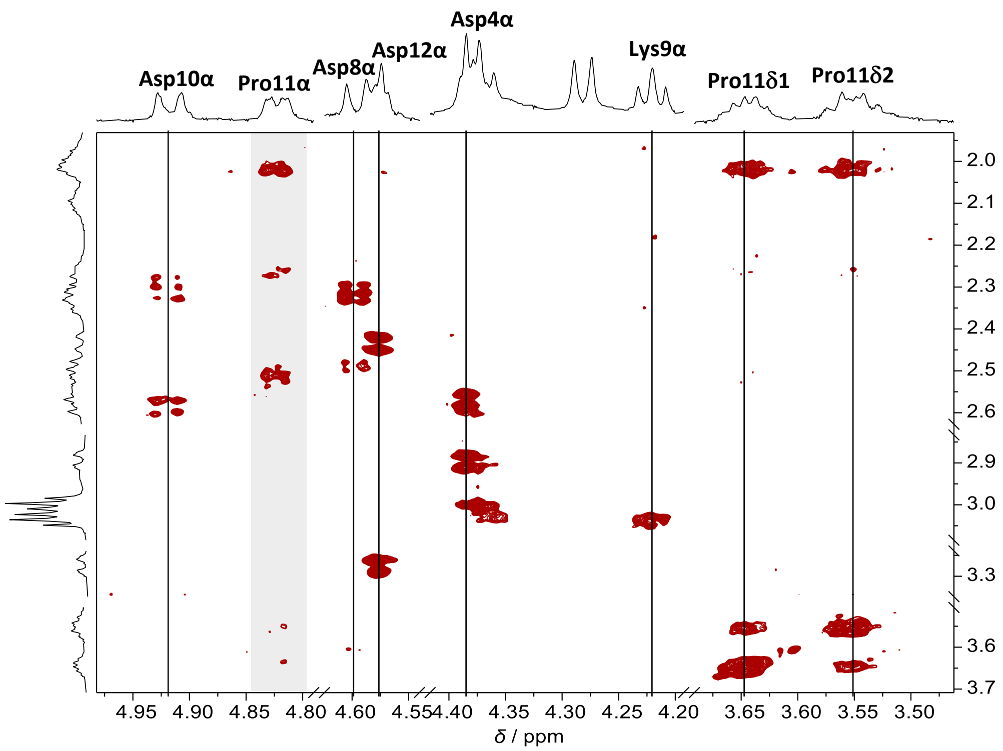

Figure S45 TOCSY spectrum of the $LuCl_3$ to EF1-R titration series endpoint sample (EF:Lu(III) ratio = 1:10) in D_2O (600 MHz, 0.003% TMSP-d_4, 30 mM MES-d_{13}, 100 mM KCl), showing selected regions for proving the correct assignment of the Asp and Pro Hα (see also Table S14). The correlation pattern characteristic for the proline spin system unambiguously assigns the signal at 4.83 ppm to the Pro11 Hα. Correspondingly, the other correlation signatures associated with Hα signals with chemical shifts between 4.92 and 4.39 ppm belong to the four aspartates, recognisable by their Hα–Hβ patterns. Evaluation of spectra associated with the free peptide and the Lu(III) to EF1-R titration endpoint spectra reveals a number of significant shifts for some amino acid signals which are listed in Table S14. These chemical shift differences relative to the metal-free peptide highlight the remarkable structural changes induced upon complexation of presumably 2 Lu(III) metal ions.

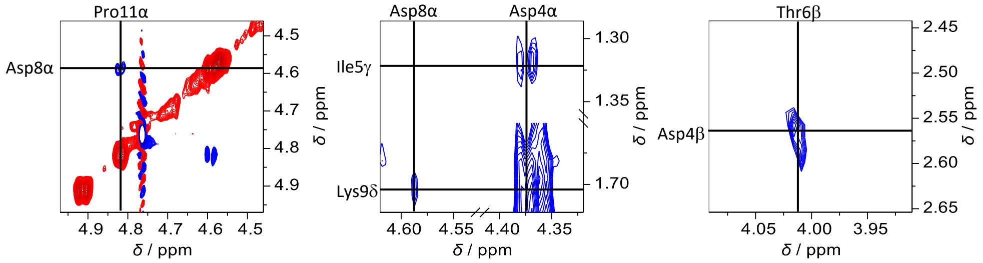

Figure S46 Selected regions of a ROESY spectrum of the $LuCl_3$ to EF1-R titration series (Figure S42) endpoint sample (EF:Lu(III) ratio = 1:10) after lyophilisation and redissolving in D_2O (600 MHz, 0.003% TMSP-d_4, 30 mM MES-d_{13}, 100 mM KCl). The ROE contacts shown in this figure allowed the correct assignment of the Asp residues (see also Figure S45 and Table S14). Unambiguous assignments: of Asp4 Hα from ROE contact to Ile5 Hγ (middle), of Asp8 Hα from ROE contacts to Lys9 Hδ (middle) and Pro11 Hα (left), and of Asp4 Hβ from ROE contact to Thr6 Hβ.

Table S14 ^{1}H NMR chemical shifts of the presumed EF1-R:Lu(III) 1:2 complex as observed in the last titration step (EF:Lu(III), ratio = 1:10) obtained from ^{1}H NMR and TOCSY experiments (600 MHz, H_2O/D_2O (9:1) or D_2O + 0.003% TMSP-d_4, 30 mM MES-d_{13}, 100 mM KCl). Observed ROE contacts between protons are indicated by the same superscripted symbols (*e.g.* *, ♣,♠ or §). For key or well observable relations, signal displacements in parts per billion (ppb) relative to the metal-free peptide are given in grey and in parentheses.

	NH	Hα	Hβ	Hγ	Hδ	Hε
**Glu1**	-	3.95	2.09, 2.02	2.52, 2.26	-	-
**Lys2**	7.96 (−230 ppb)	4.36	1.74	1.45	1.68	3.00
**Leu3**	7.92 (−570 ppb)	4.17		1.57	0.90, 0.96	-
**Asp 4**	8.95 (+530 ppb)	4.39* (−278 ppb)	2.90, 2.57§ (+179/−28 ppb)	-	-	-
**Ile5**	7.58 (−560 ppb)	4.28	1.85	1.42, 1.32*, 0.93	0.83	-
**Thr6**	8.45 (+160 ppb)	4.22	4.02§	1.23	-	-
**Gly7**	*not observed*	3.99, 3.87	-	-	-	-
**Asp8**	8.26 (±0 ppb)	4.60♣♠ (−10 ppb)	2.49, 2.31 (−283/−192)	-	-	-
**Lys9**	7.97 (−230 ppb)	4.22	1.80	1.49	1.72♣	3.04
**Asp 10**	8.88 (+420 ppb)	4.92 (+74 ppb)	2.59, 2.28 (−226/−156)	-	-	-
**Pro11**	-	4.82♠ (+382 ppb)	2.52, 2.26	2.02	3.65, 3.55 (−197 ppb/−177 ppb)	-
**Asp12**	7.40 (−590 ppb)	4.57 (+220 ppb)	3.27, 2.43 (+591/−130)	-	-	-

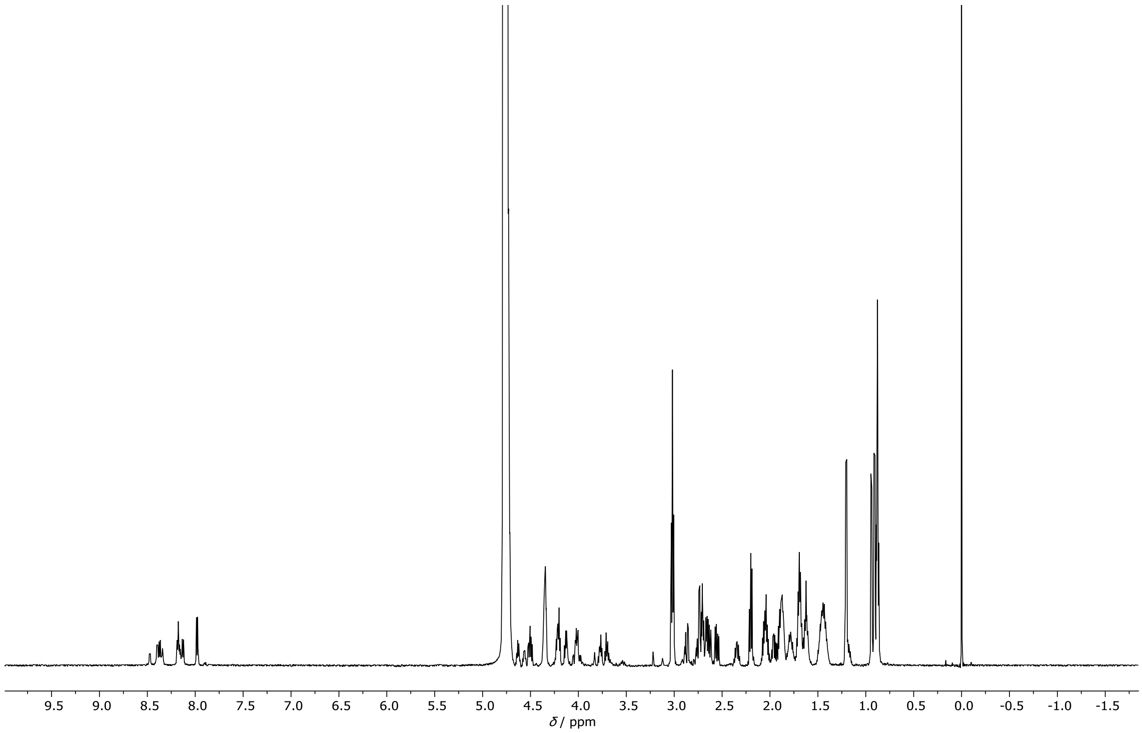

Figure S47 ^{1}H NMR spectrum of metal-free EF1 (600 MHz, H_2O/D_2O (9:1) + 0.003% TMSP-d_4, 30 mM MES-d_{13}, 100 mM KCl).

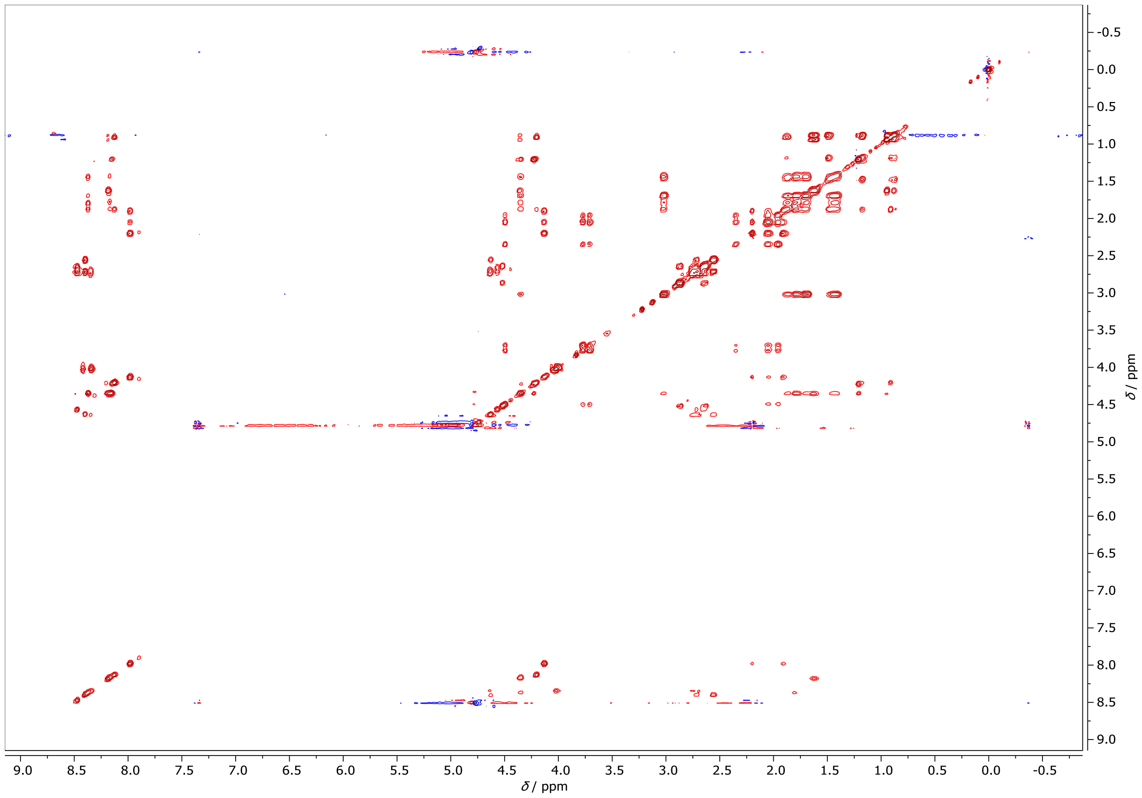

Figure S48 TOCSY spectrum of metal-free EF1 (600 MHz, H_2O/D_2O (9:1) + 0.003% TMSP-d_4, 30 mM MES-d_{13}, 100 mM KCl).

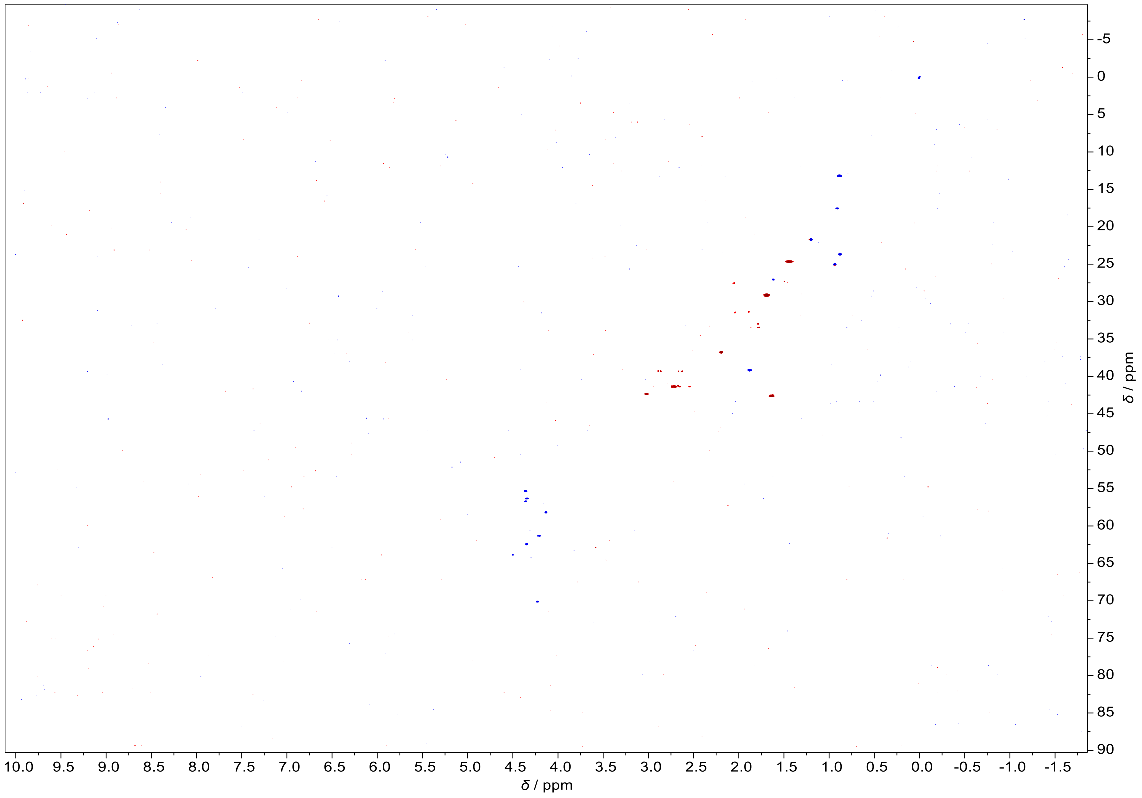

Figure S49 HSQC spectrum of metal-free EF1 (600 MHz, H_2O/D_2O (9:1) + 0.003% TMSP-d_4, 30 mM MES-d_{13}, 100 mM KCl).

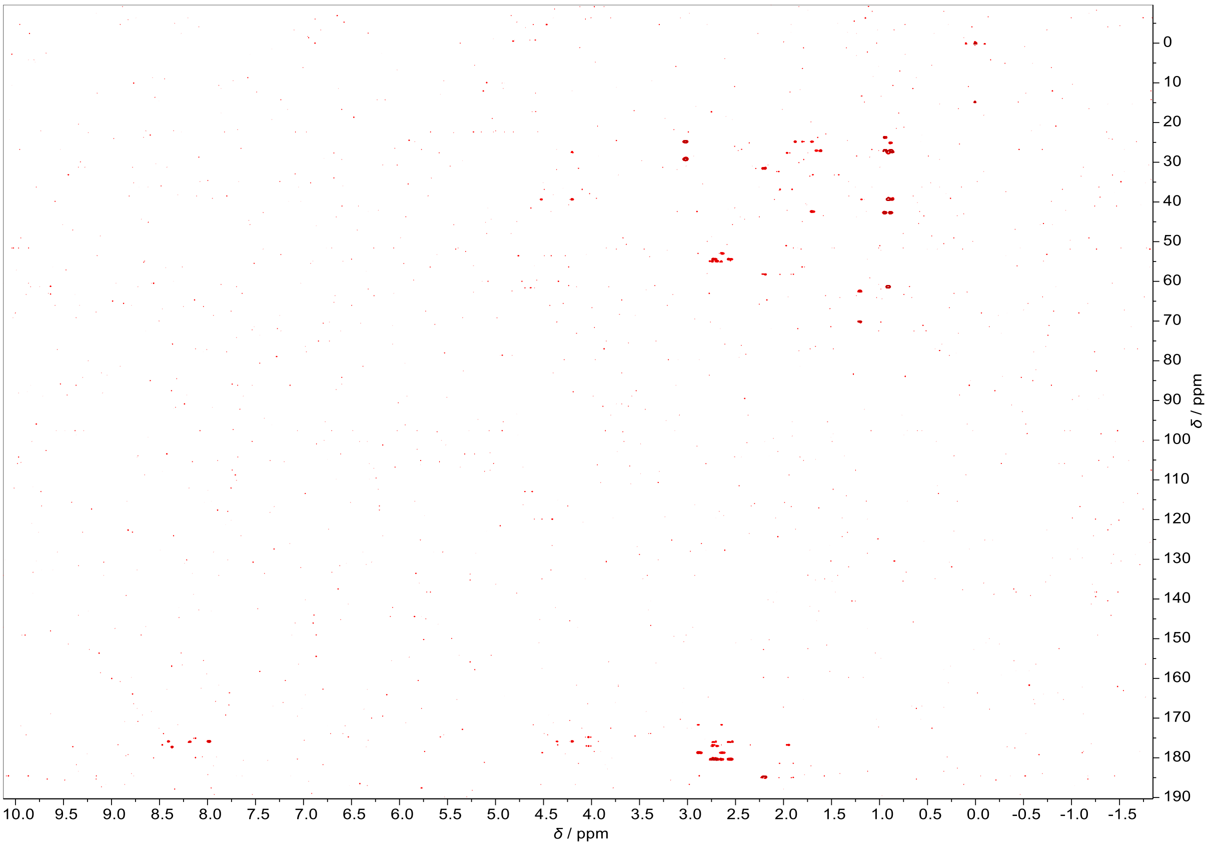

Figure S50 HMBC spectrum of metal-free EF1 (600 MHz, H_2O/D_2O (9:1) + 0.003% TMSP-d_4, 30 mM MES-d_{13}, 100 mM KCl).

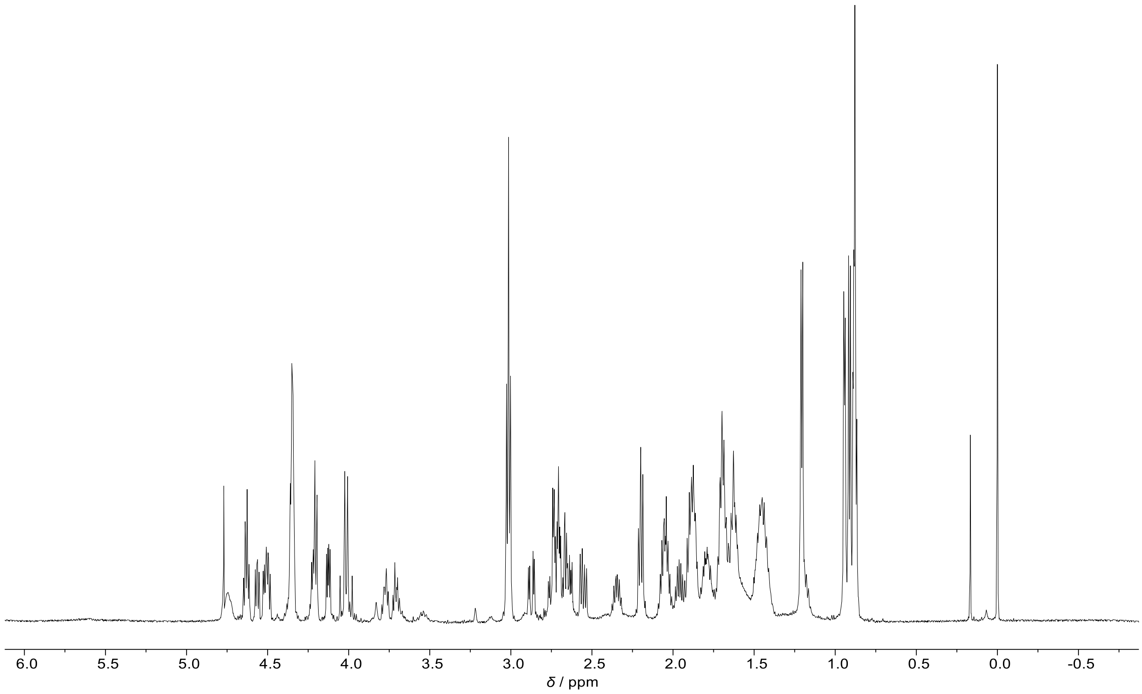

Figure S51 ^{1}H NMR spectrum of metal-free EF1 redissolved in D_2O after lyophilisation (600 MHz, D_2O + 0.003% TMSP-d_4, 30 mM MES-d_{13}, 100 mM KCl).

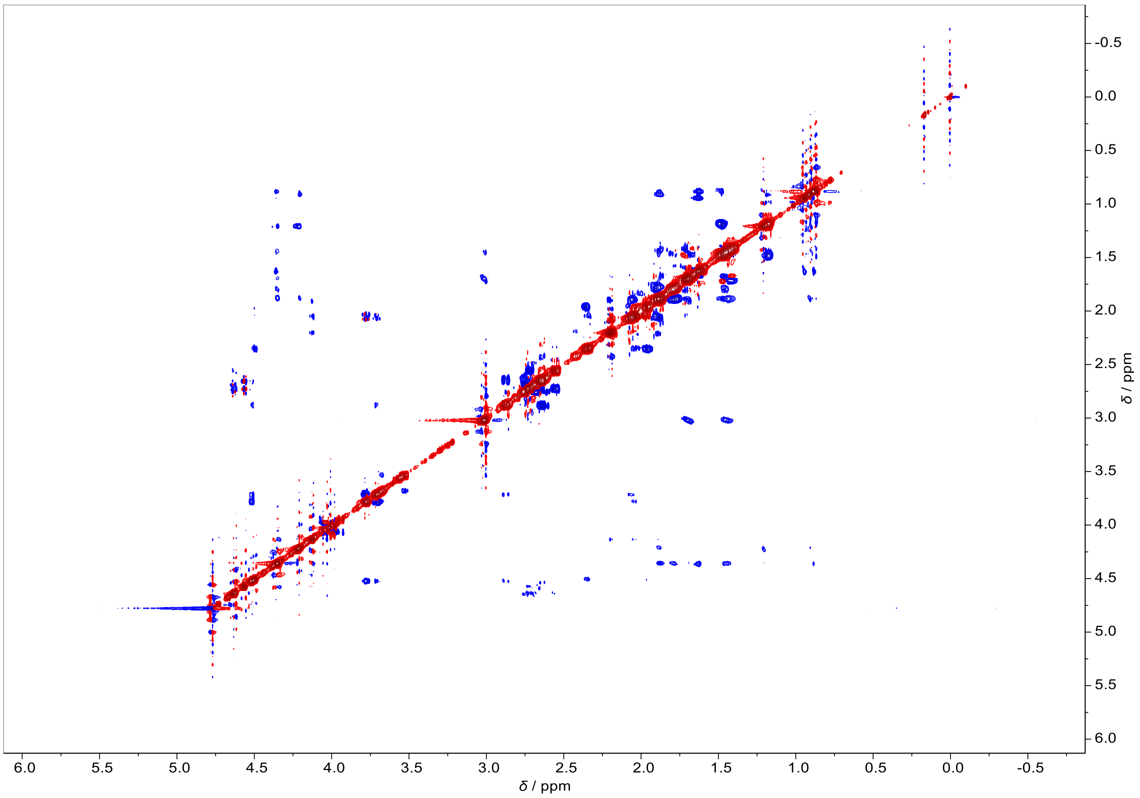

Figure S52 ROESY spectrum of metal-free EF1 redissolved in D_2O after lyophilisation (600 MHz, D_2O + 0.003% TMSP-d_4, 30 mM MES-d_{13}, 100 mM KCl).

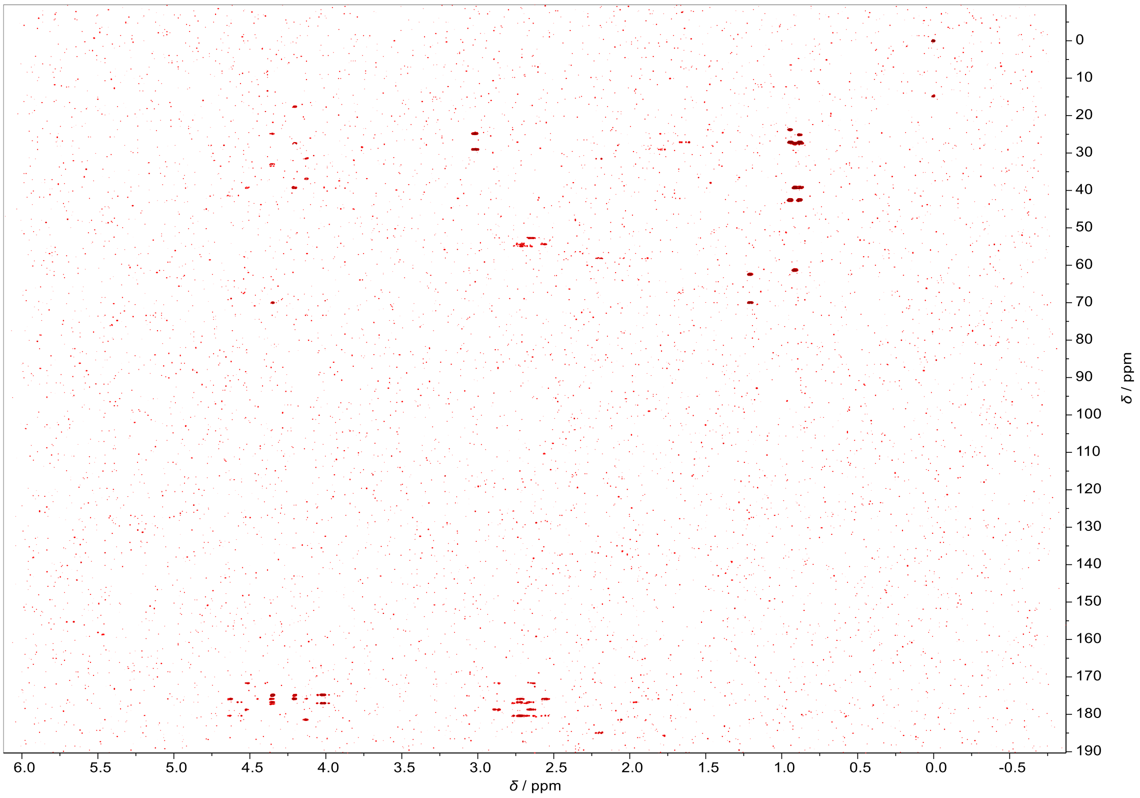

Figure S53 HMBC spectrum of metal-free EF1 redissolved in D_2O after lyophilisation (600 MHz, D_2O + 0.003% TMSP-d_4, 30 mM MES-d_{13}, 100 mM KCl).

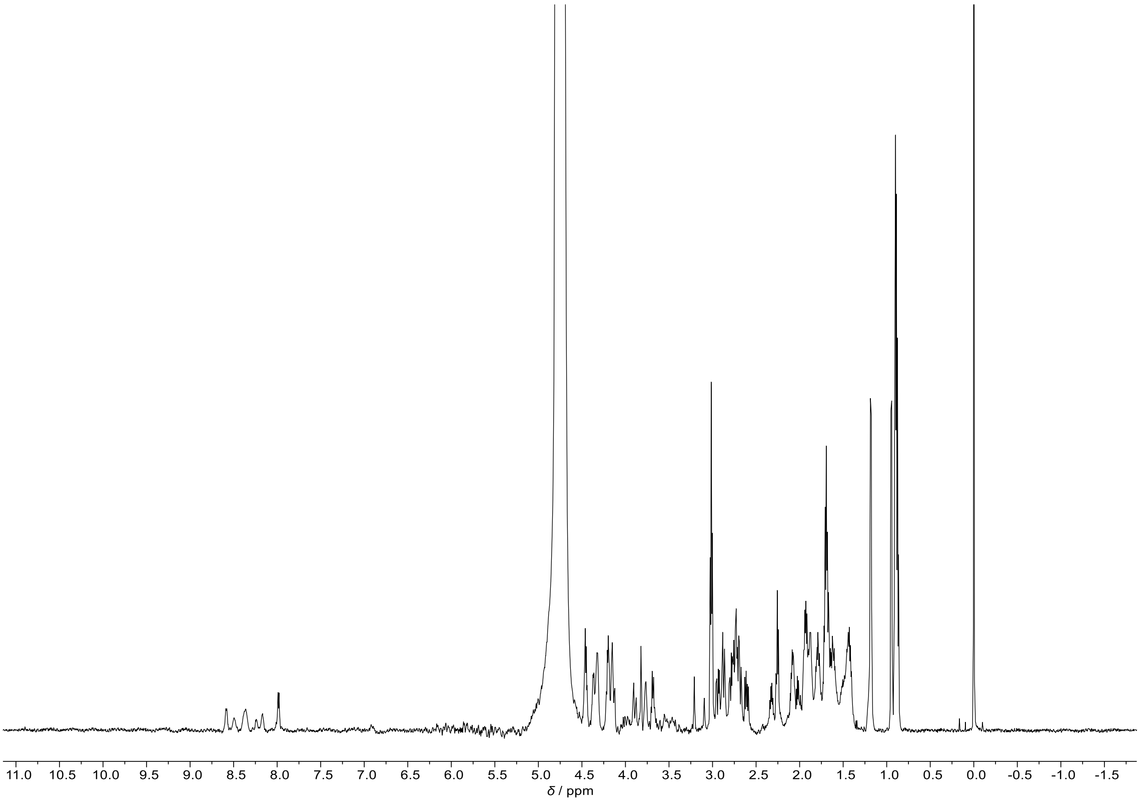

Figure S54 ^{1}H NMR spectrum of the last titration step (1:10 EF:La(III) ratio) of the La(III) to EF1 titration series (Figure S36) measured with a higher number of scans (600 MHz, H_2O/D_2O (9:1) + 0.003% TMSP-d_4, 30 mM MES-d_{13}, 100 mM KCl, pH 6.6).

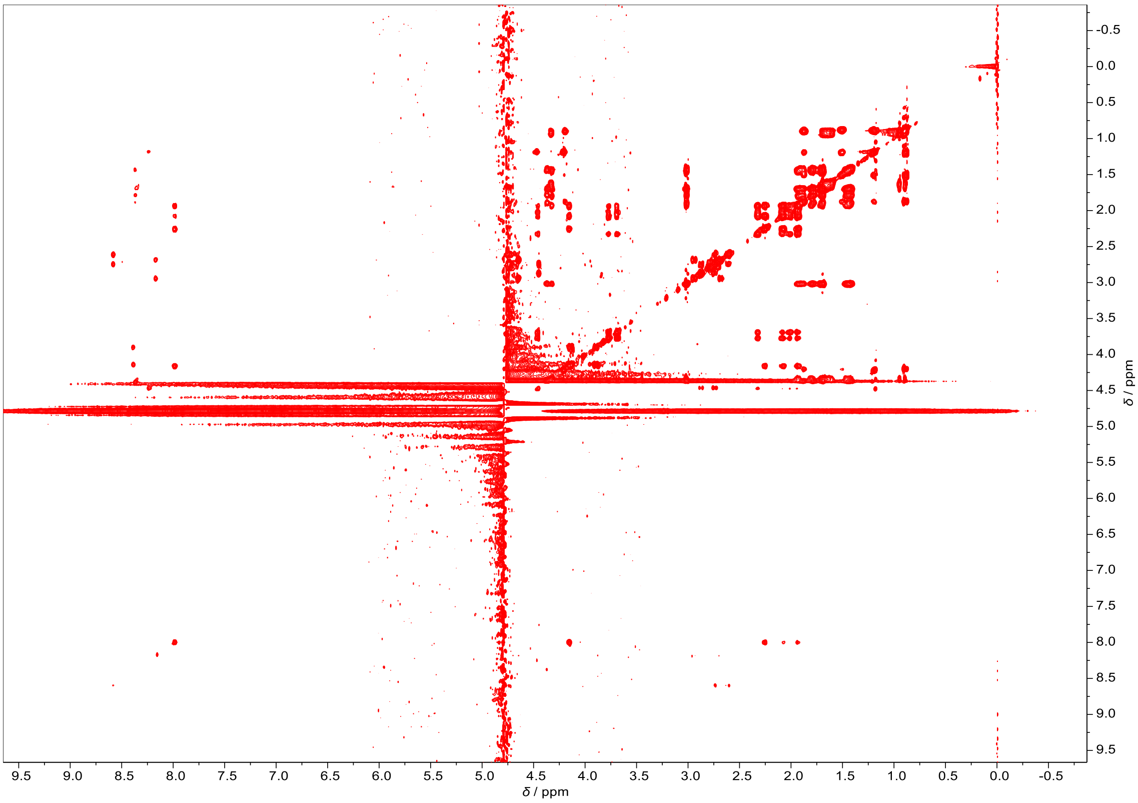

Figure S55 TOCSY spectrum of the last titration step (1:10 EF:La(III) ratio) of the $LaCl_3$ to EF1 titration series (Figure S36) (600 MHz, H_2O/D_2O (9:1) + 0.003% TMSP-d_4, 30 mM MES-d_{13}, 100 mM KCl, pH 6.6).

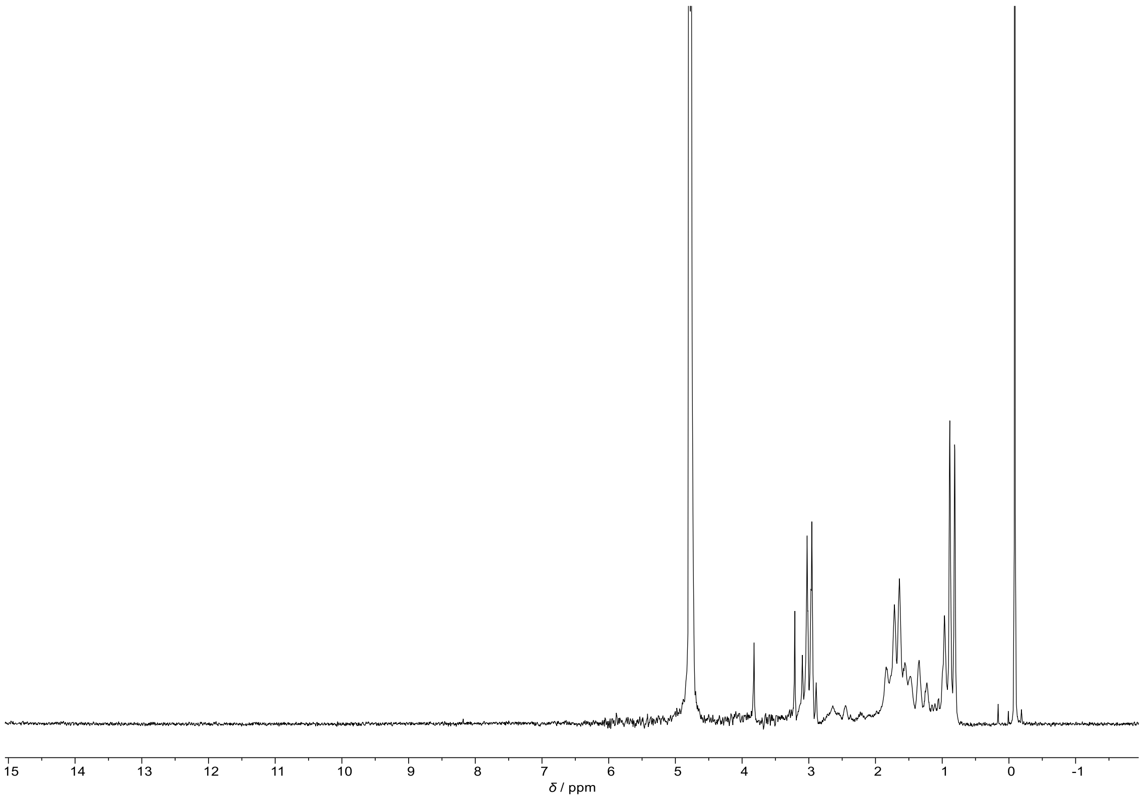

Figure S56 ^{1}H NMR spectrum of the last titration step (1:10 EF:Eu(III) ratio) of the $EuCl_3$ to EF1 titration series (Figure S37) measured with a higher number of scans (600 MHz, H_2O/D_2O (9:1) + 0.003% TMSP-*d*$_4$, 30 mM MES-*d*$_{13}$, 100 mM KCl, pH 6.6). Referenced to the buffer signal at 3.21 ppm as described in Figure S37 and S39.

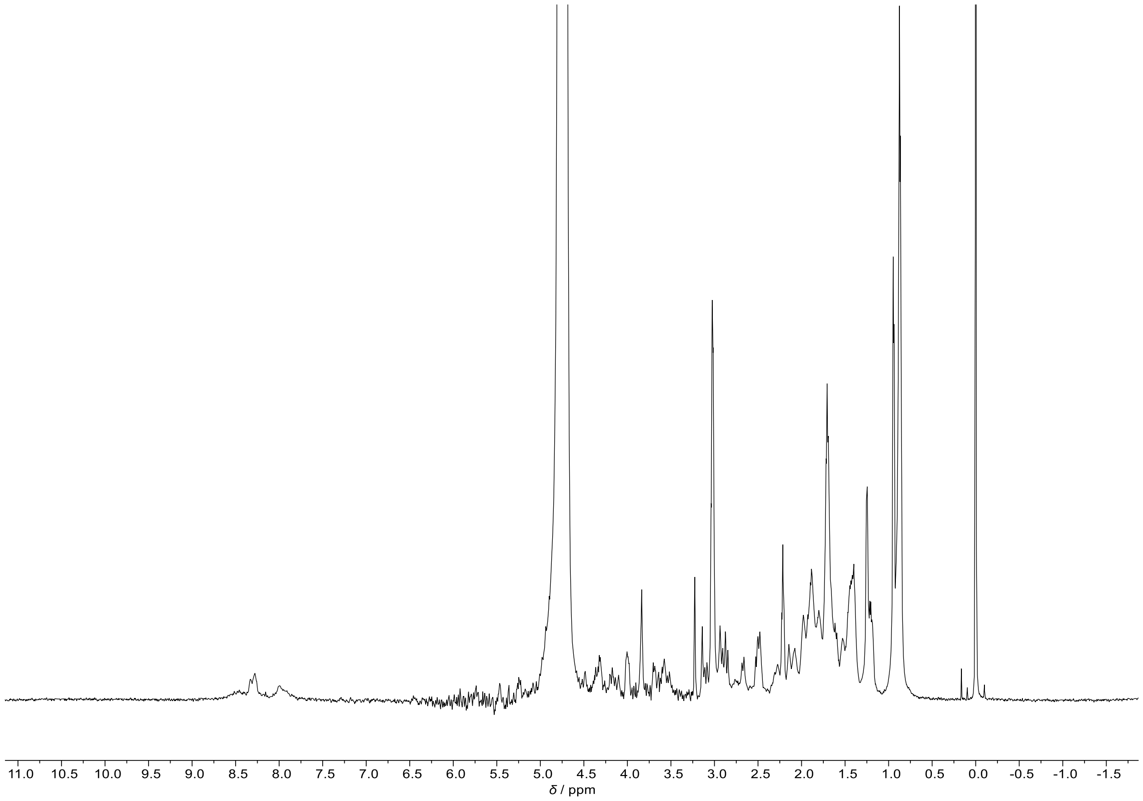

Figure S57 ^{1}H NMR spectrum of the last titration step (1:10 EF:Lu(III) ratio) of the $LuCl_3$ to EF1 titration series (Figure S38) measured with a higher number of scans (600 MHz, H_2O/D_2O (9:1) + 0.003% TMSP-*d*$_4$, 30 mM MES-*d*$_{13}$, 100 mM KCl, pH 6.6).

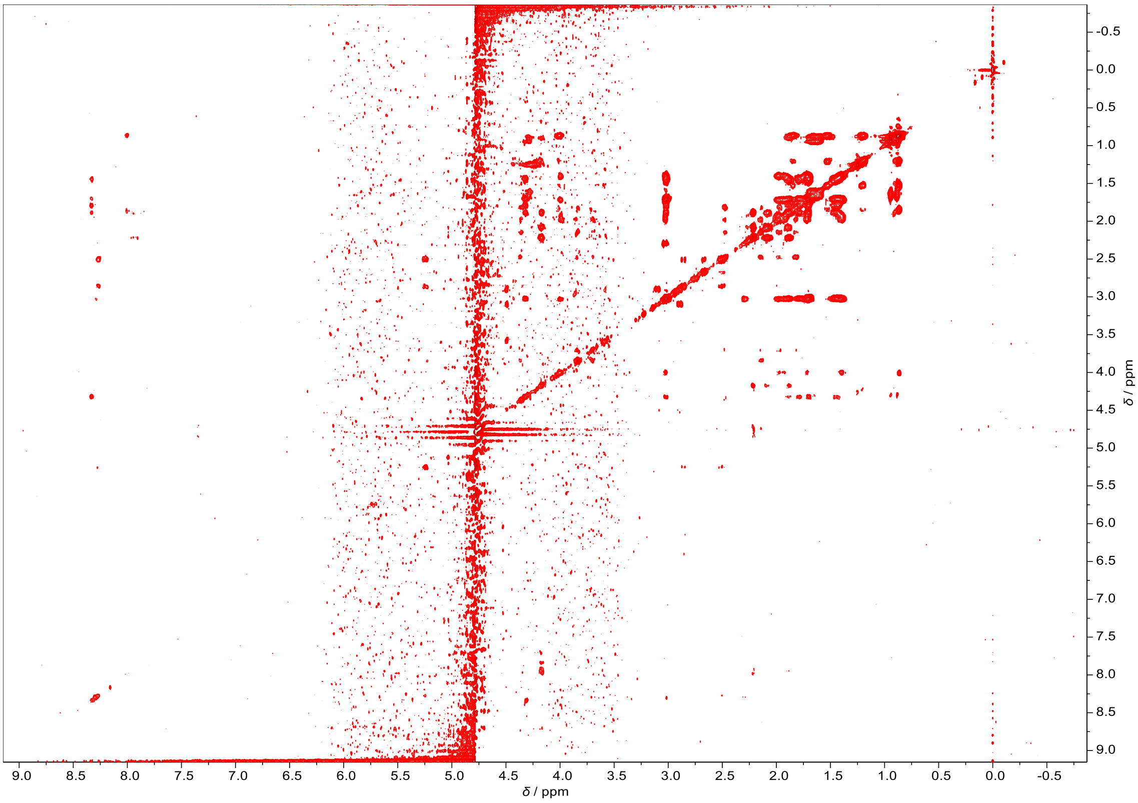

Figure S58 TOCSY spectrum of the last titration step (1:10 EF:Lu(III) ratio) of the $LuCl_3$ to EF1 titration series shown in Figure S38 (600 MHz, H_2O/D_2O (9:1) + 0.003% TMSP-d_4, 30 mM MES-d_{13}, 100 mM KCl, pH 6.6).

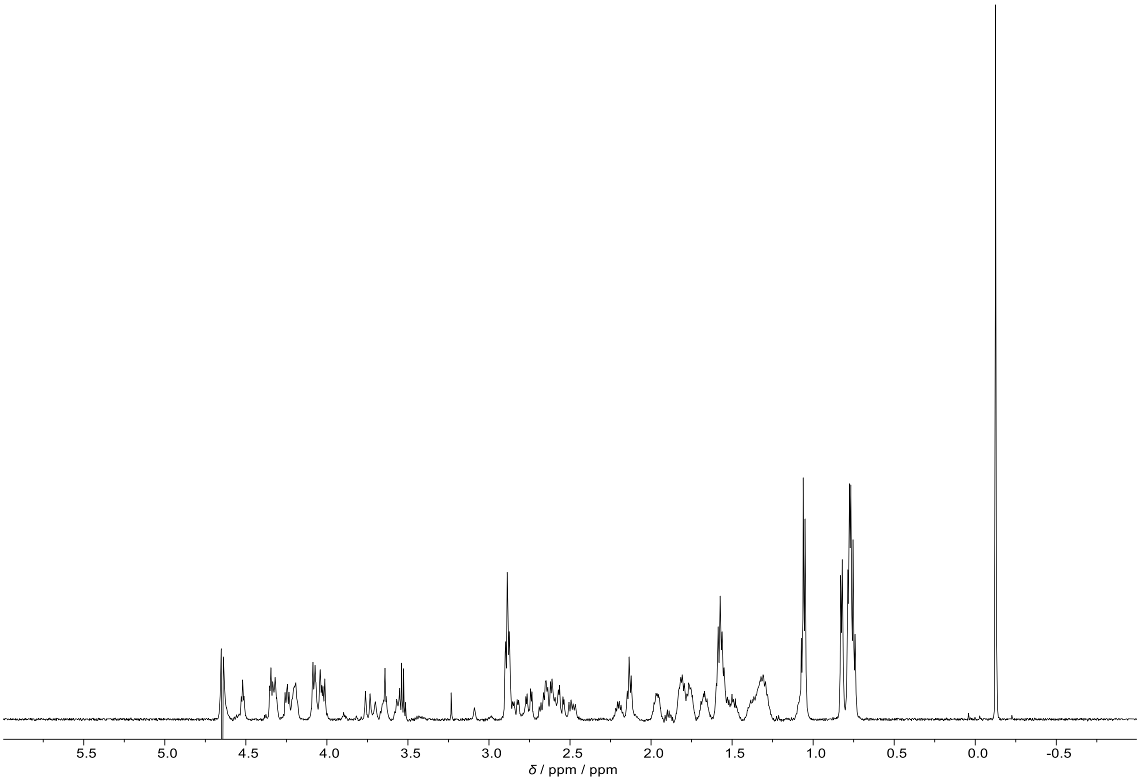

Figure S59 ^1H NMR spectrum of the last titration step (1:10 EF:La(III) ratio) of the La(III) to EF1 titration series (Figure S36) after lyophilisation and redissolving in the same volume of D_2O (600 MHz, 0.003% TMSP-d_4, 30 mM MES-d_{13}, 100 mM KCl).

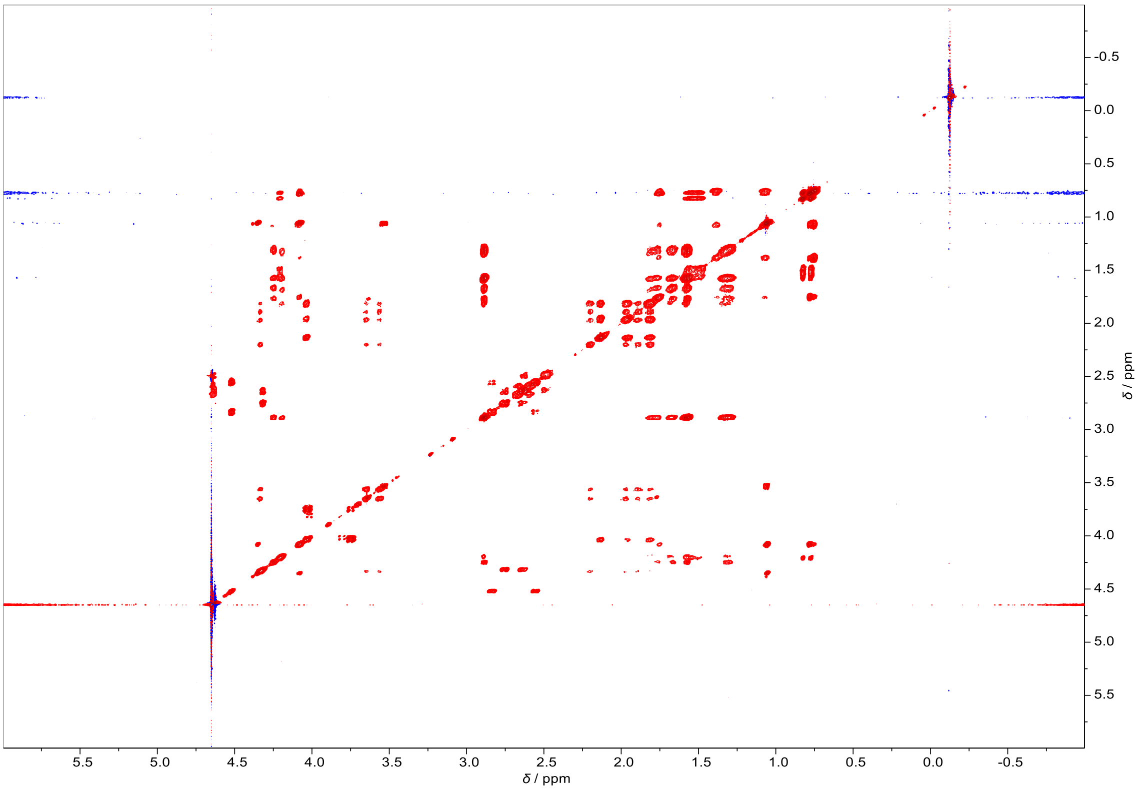

Figure S60 TOCSY spectrum of the last titration step (1:10 EF:La(III) ratio) of the La(III) to EF1 titration series (Figure S36) after lyophilisation and in the same volume of D_2O (600 MHz, 0.003% TMSP, 30 mM MES-d_{13}, 100 mM KCl).

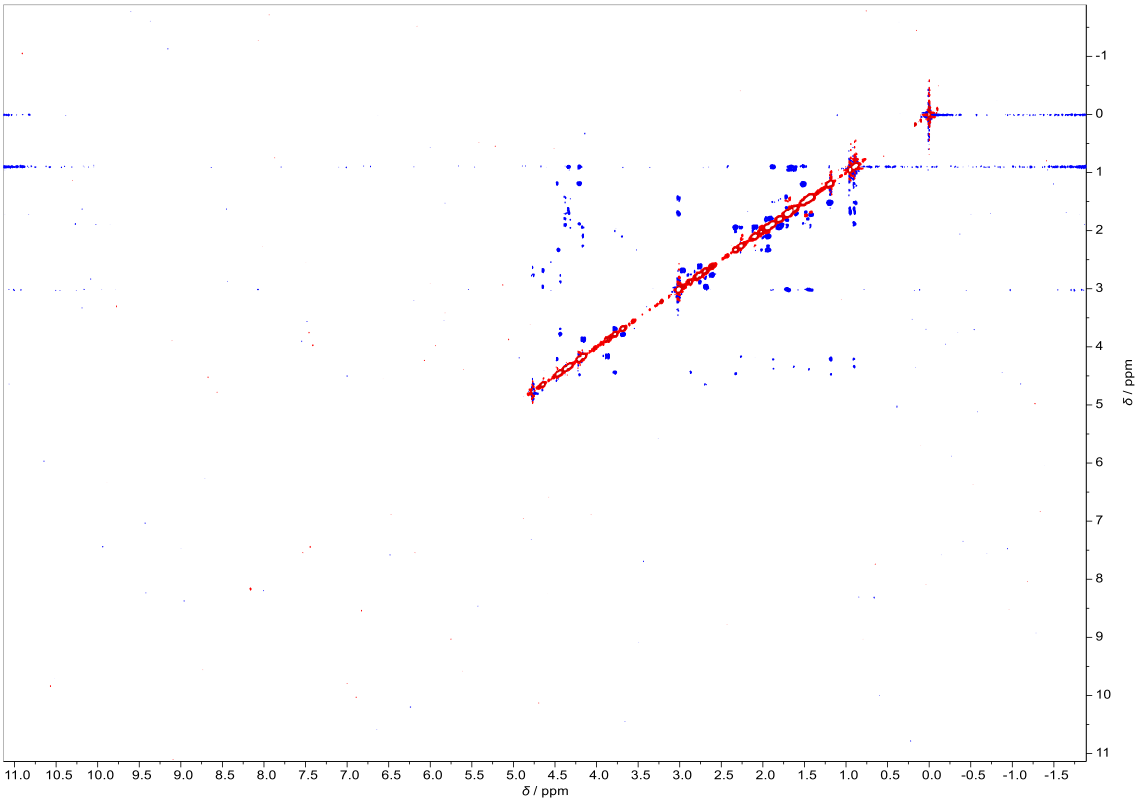

Figure S61 ROESY spectrum of the last titration step (1:10 EF:La(III) ratio) of the La(III) to EF1 titration series (Figure S36) after lyophilisation and in the same volume of D_2O (600 MHz, 0.003% TMSP-d_4, 30 mM MES-d_{13}, 100 mM KCl).

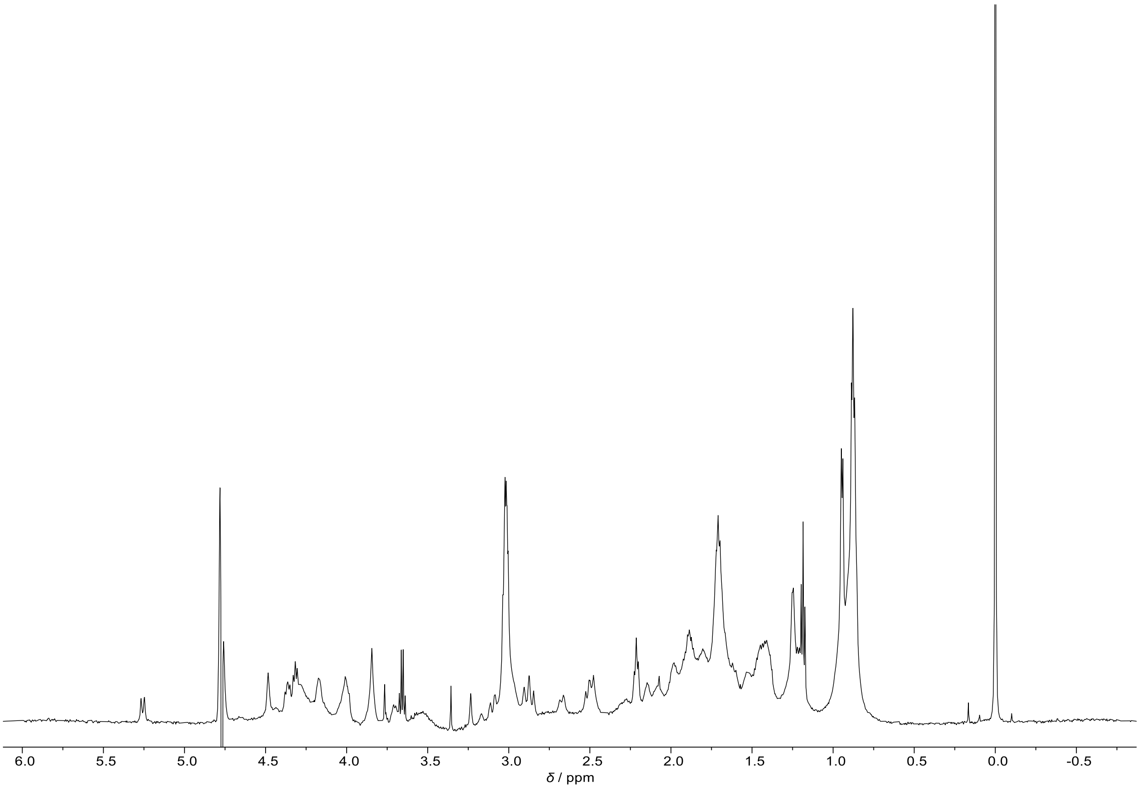

Figure S62 ^{1}H NMR spectrum of the last titration step (1:10 EF:Lu(III) ratio) of the Lu(III) to EF1 titration series (Figure S38) after lyophilisation and in the same volume of D_2O (600 MHz, 0.003% TMSP-d_4, 30 mM MES-d_{13}, 100 mM KCl).

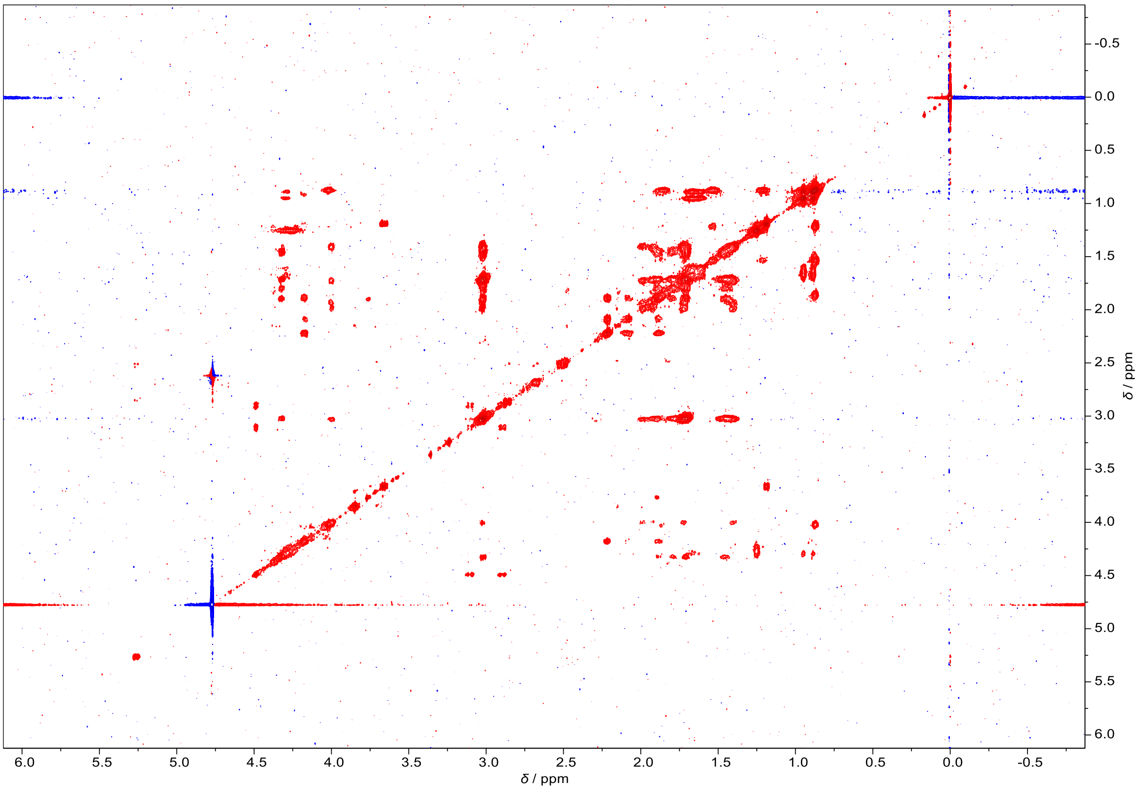

Figure S63 TOCSY spectrum of the last titration step (1:10 EF:Lu(III) ratio) of the Lu(III) to EF1 titration series (Figure S38) after lyophilisation and in the same volume of D_2O (600 MHz, 0.003% TMSP-d_4, 30 mM MES-d_{13}, 100 mM KCl).

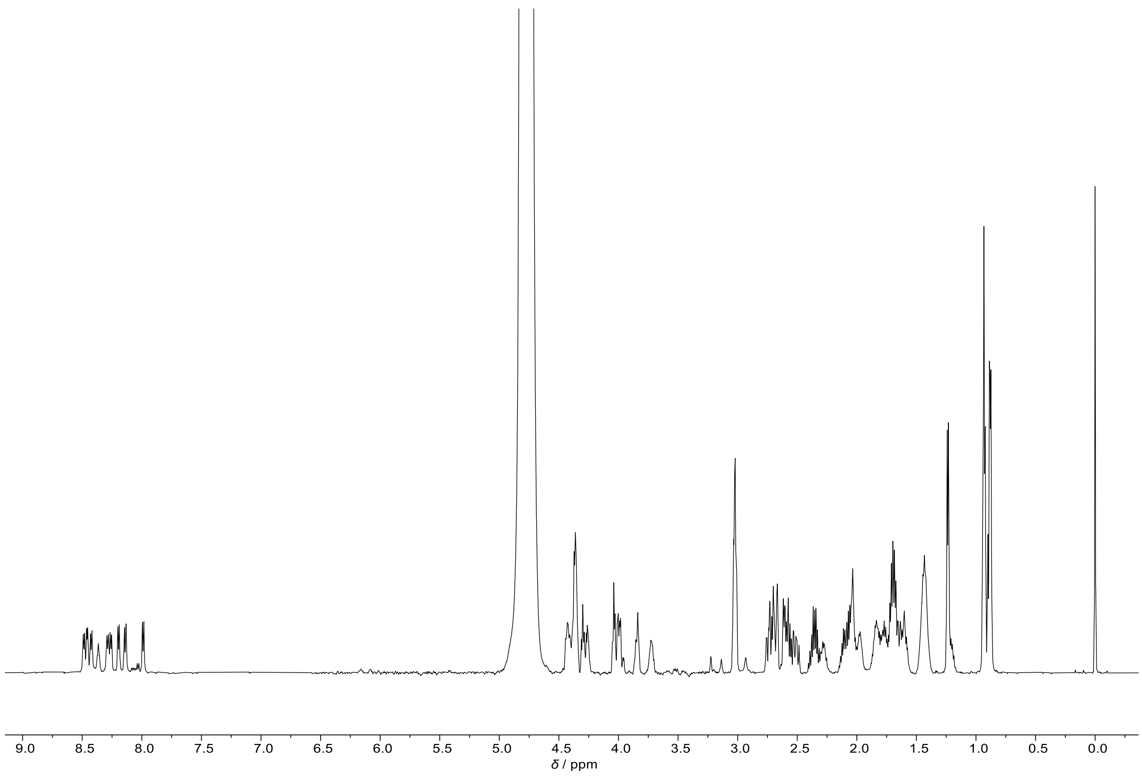

Figure S64 ^{1}H NMR spectrum of free EF1-R (600 MHz, H_2O/D_2O (9:1) + 0.003% TMSP-*d*$_4$, 30 mM MES-*d*$_{13}$, 100 mM KCl).

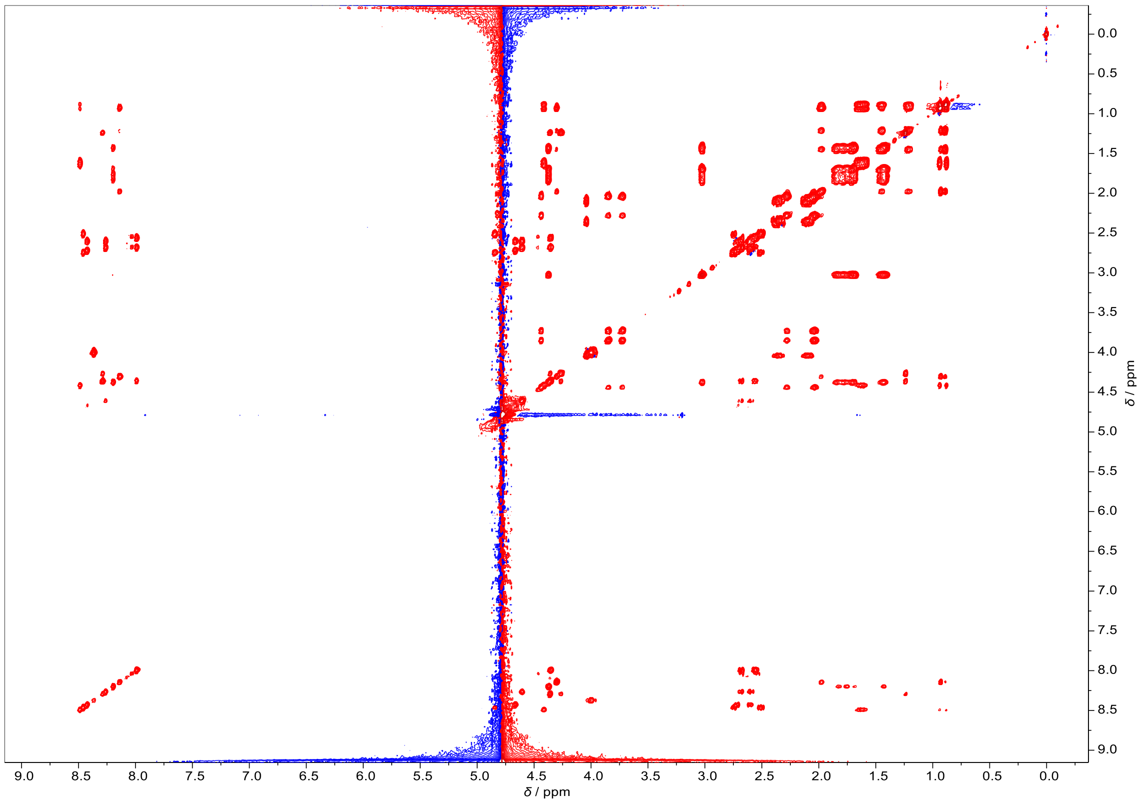

Figure S65 TOCSY spectrum of free EF1-R (600 MHz, H_2O/D_2O (9:1) + 0.003% TMSP-*d*$_4$, 30 mM MES-*d*$_{13}$, 100 mM KCl).

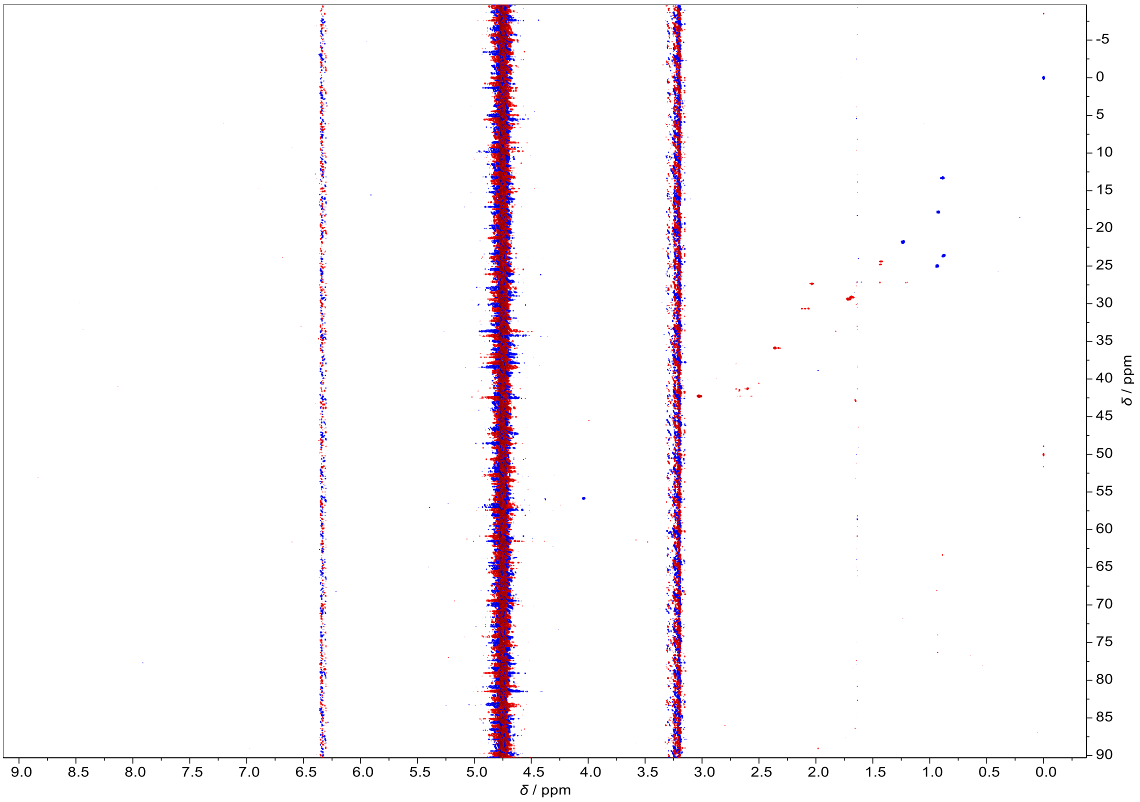

Figure S66 HSQC spectrum of free EF1-R (600 MHz, H_2O/D_2O (9:1) + 0.003% TMSP-d_4, 30 mM MES-d_{13}, 100 mM KCl).

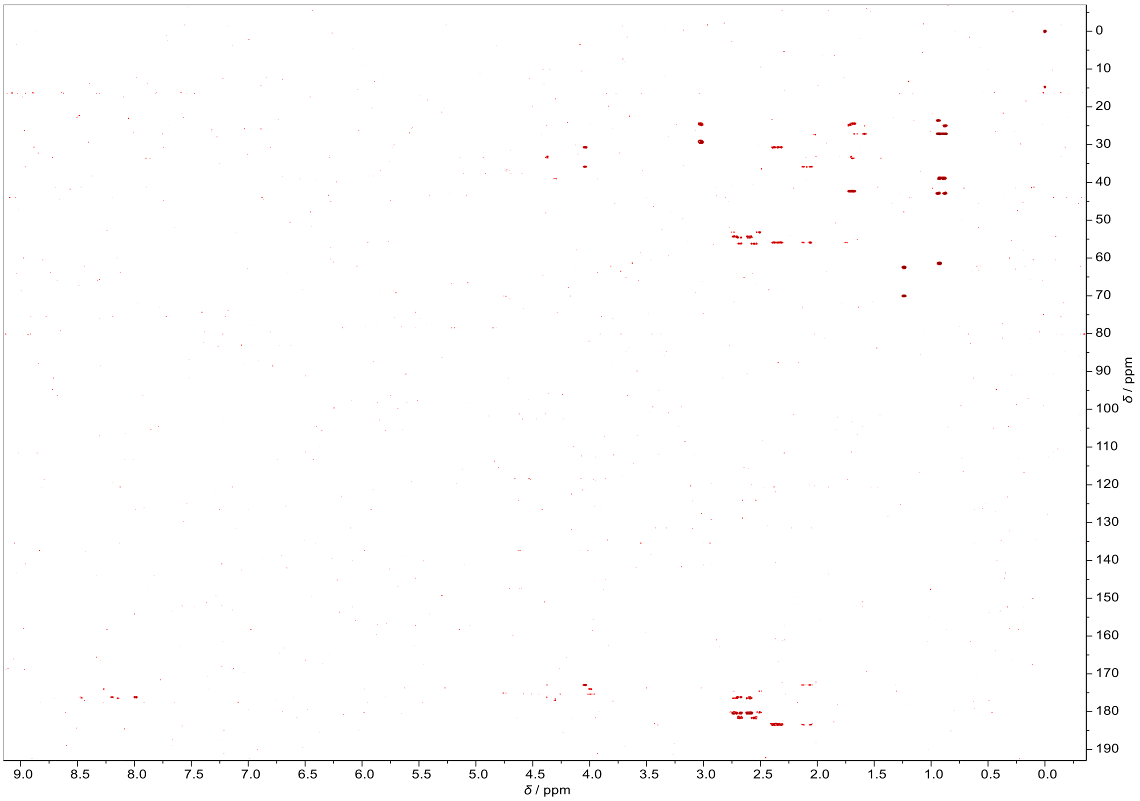

Figure S67 HMBC spectrum of free EF1-R (600 MHz, H_2O/D_2O (9:1) + 0.003% TMSP-d_4, 30 mM MES-d_{13}, 100 mM KCl).

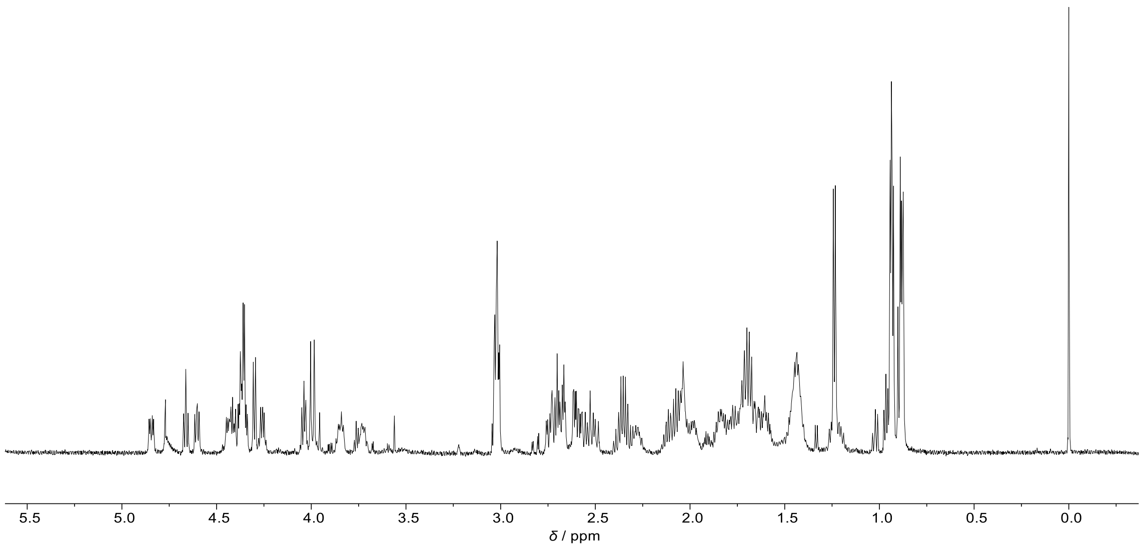

Figure S68 ^{1}H NMR spectrum of free EF1-R redissolved in the same volume of D_2O after lyophilisation (600 MHz, D_2O + 0.003% TMSP-d_4, 30 mM MES-d_{13}, 100 mM KCl).

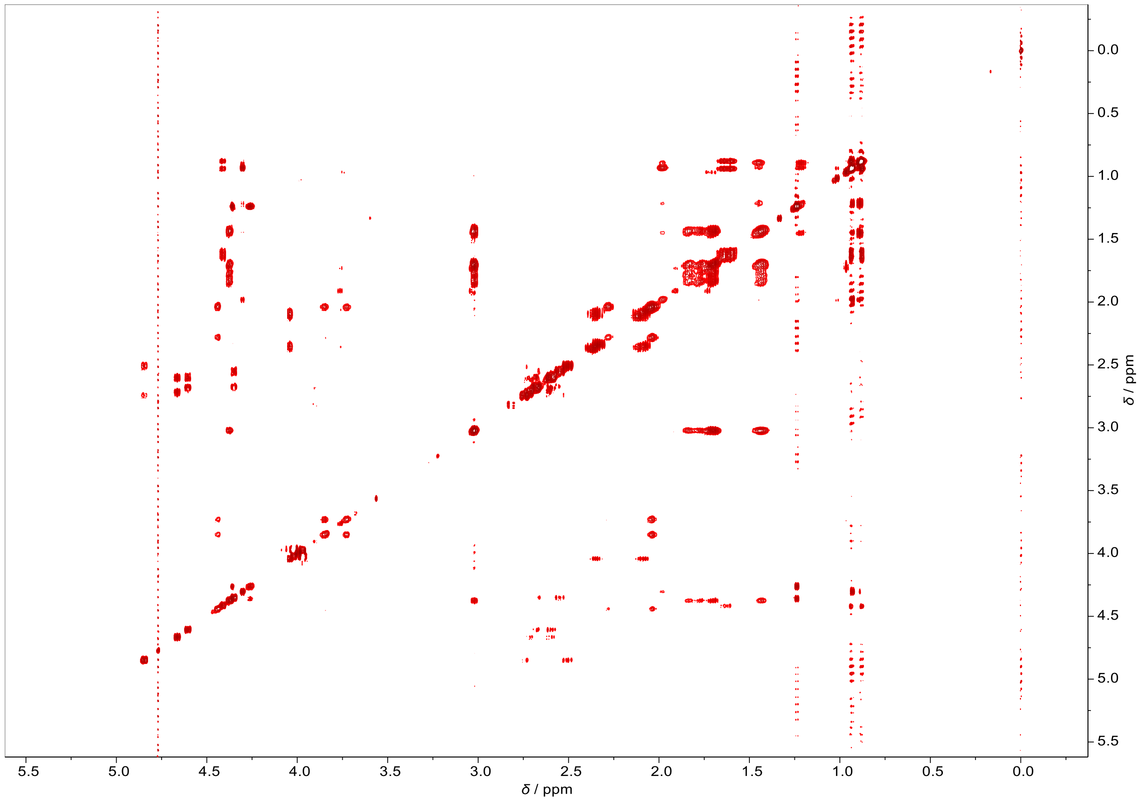

Figure S69 TOCSY spectrum of free EF1-R redissolved in the same volume of D_2O after lyophilisation (600 MHz, D_2O + 0.003% TMSP-d_4, 30 mM MES-d_{13}, 100 mM KCl).

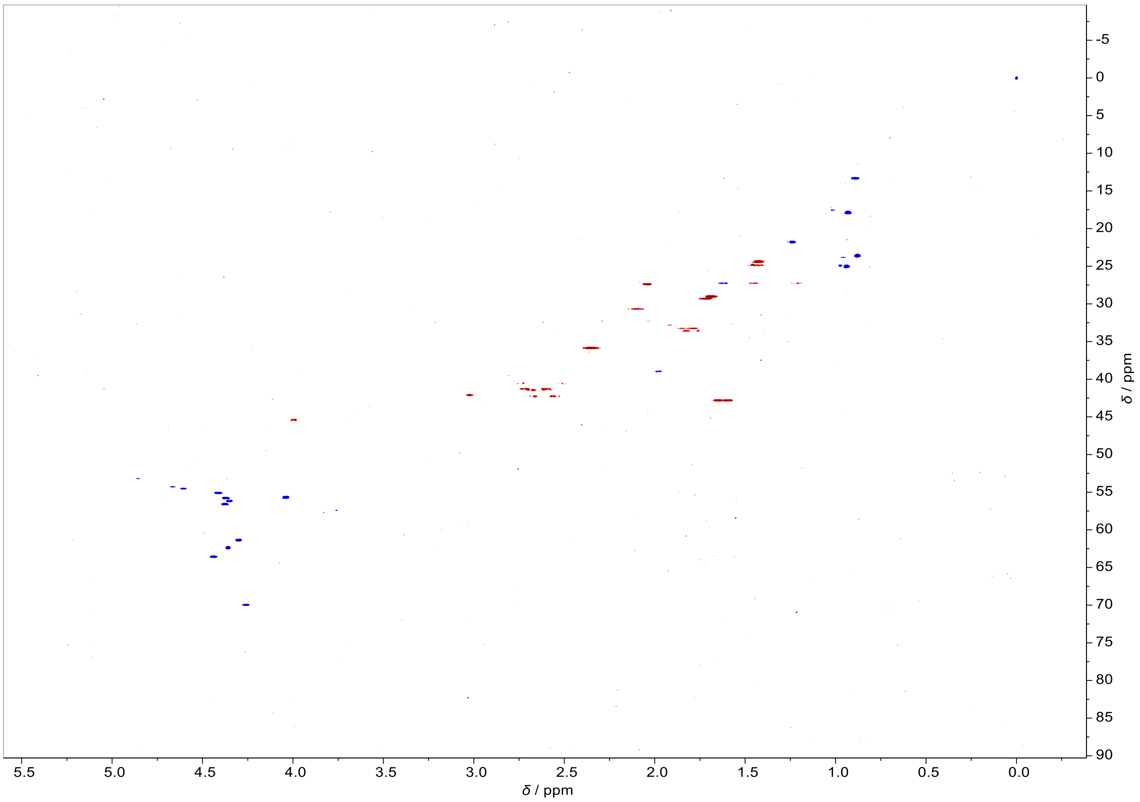

Figure S70 HSQC spectrum of free EF1-R redissolved in the same volume of D_2O after lyophilisation (600 MHz, D_2O + 0.003% TMSP-d_4, 30 mM MES-d_{13}, 100 mM KCl).

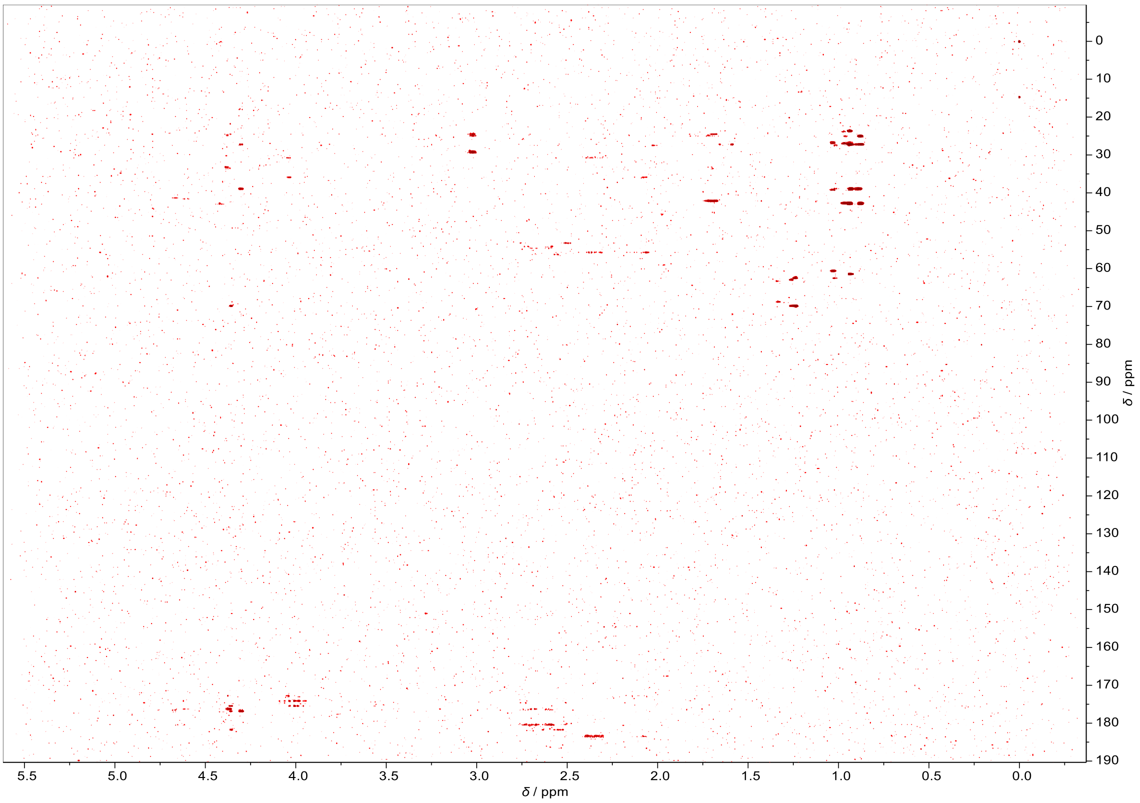

Figure S71 HMBC spectrum of free EF1-R redissolved in the same volume of D_2O after lyophilisation (600 MHz, D_2O + 0.003% TMSP-d_4, 30 mM MES-d_{13}, 100 mM KCl).

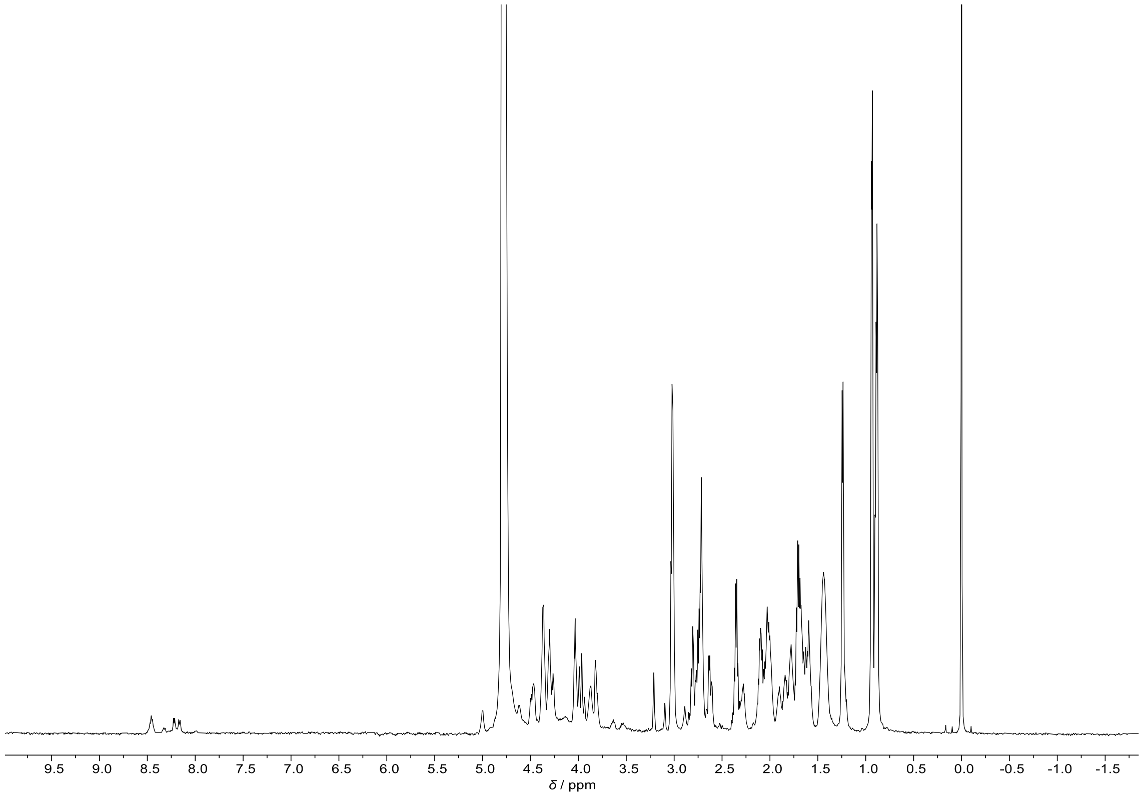

Figure S72 ^{1}H NMR spectrum of the last titration step (1:10 EF:La(III) ratio) of the $LaCl_3$ to EF1-R titration series (Figure S40) measured with a higher number of scans (600 MHz, H_2O/D_2O (9:1) + 0.003% TMSP-d_4, 30 mM MES-d_{13}, 100 mM KCl, pH 6.6).

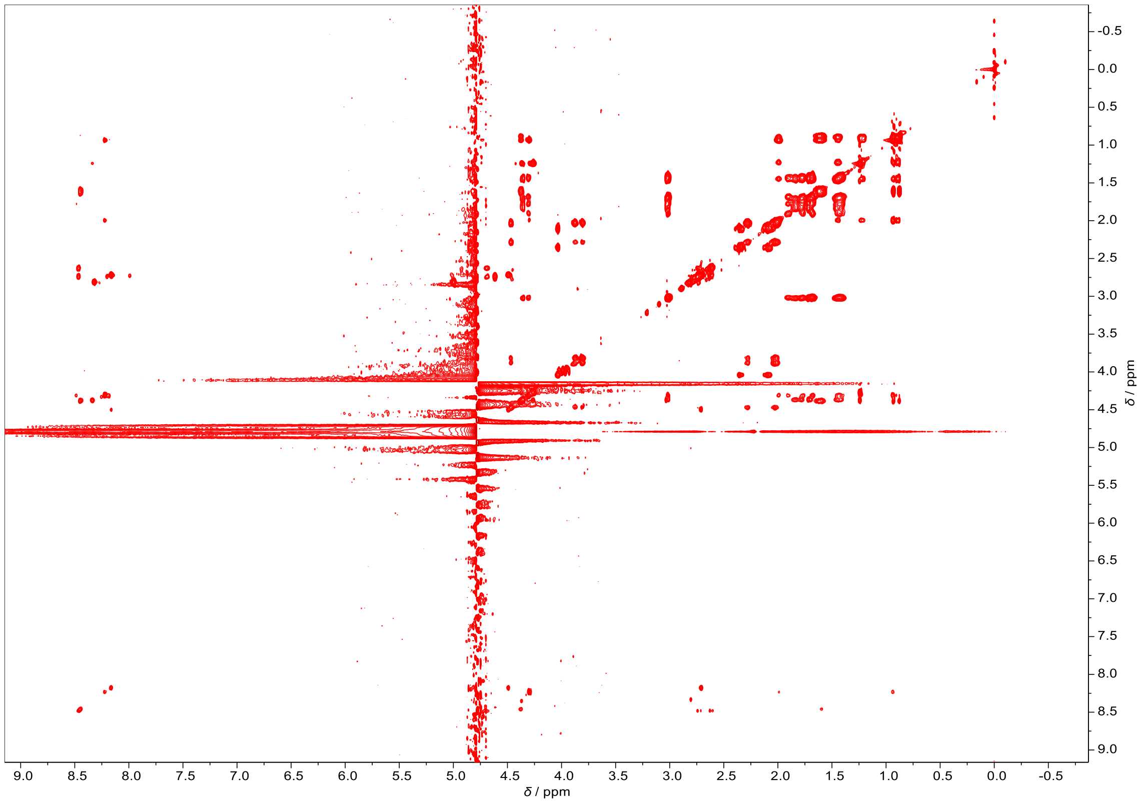

Figure S73 TOCSY spectrum of the last titration step (1:10 EF:La(III) ratio) of the $LaCl_3$ to EF1-R titration series shown in Figure S40 (600 MHz, H_2O/D_2O (9:1) + 0.003% TMSP-d_4, 30 mM MES-d_{13}, 100 mM KCl, pH 6.6).

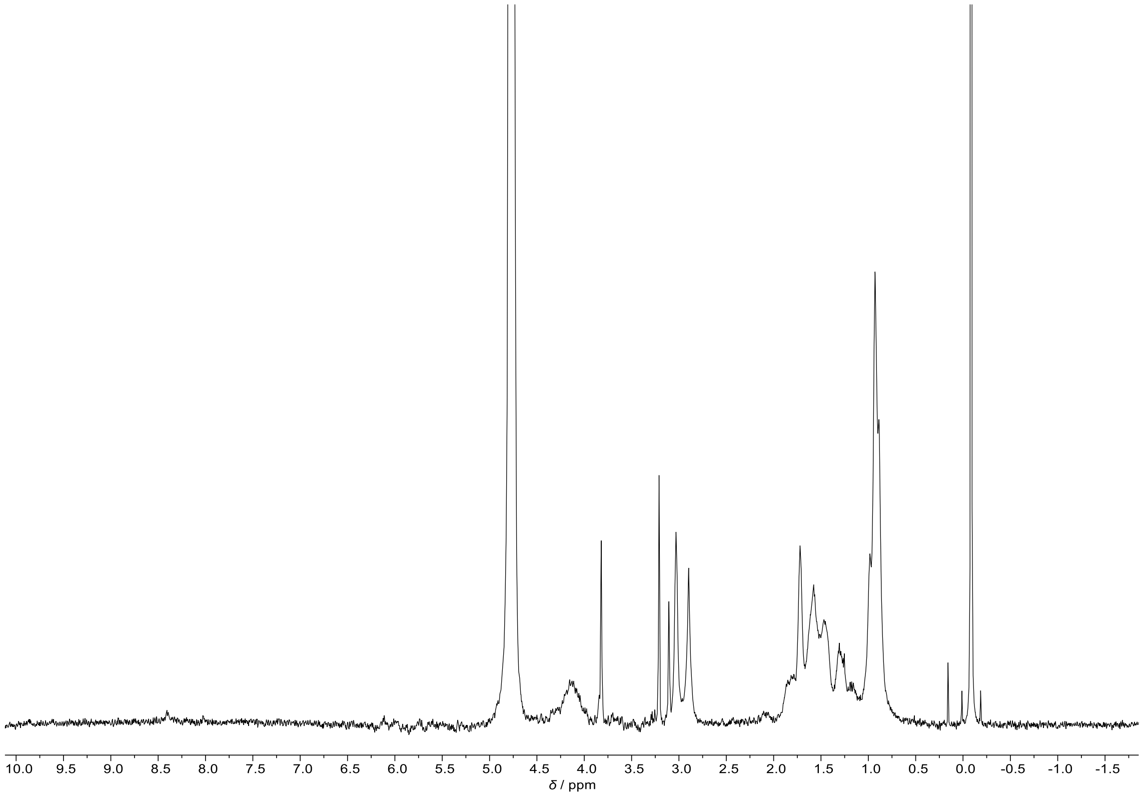

Figure S74 ^{1}H NMR spectrum of the last titration step (1:10 EF:Eu(III) ratio) of the $EuCl_3$ to EF1-R titration series (Figure S41) measured with a higher number of scans (600 MHz, H_2O/D_2O (9:1) + 0.003% TMSP-d_4, 30 mM MES-d_{13}, 100 mM KCl, pH 6.6).

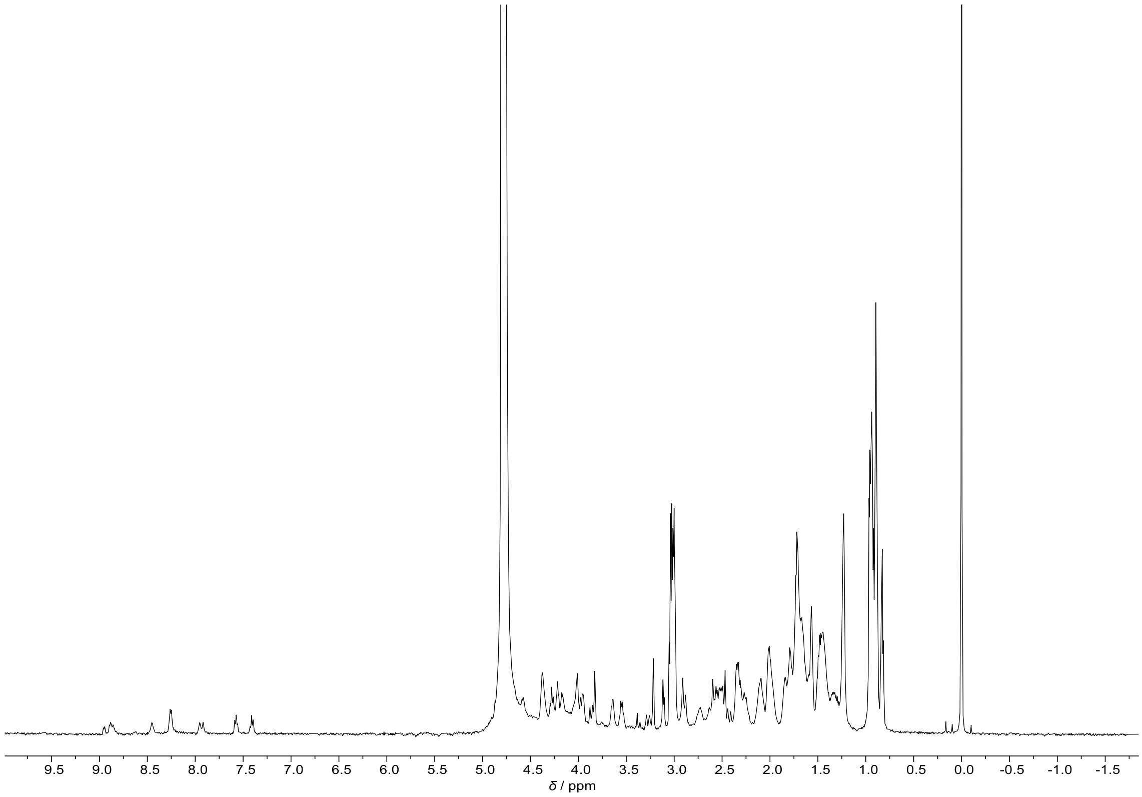

Figure S75 ^{1}H NMR spectrum of the last titration step (1:10 EF:Lu(III) ratio) of the $LuCl_3$ to EF1-R titration series (Figure S42) measured with a higher number of scans (600 MHz, H_2O/D_2O (9:1) + 0.003% TMSP-d_4, 30 mM MES-d_{13}, 100 mM KCl, pH 6.6).

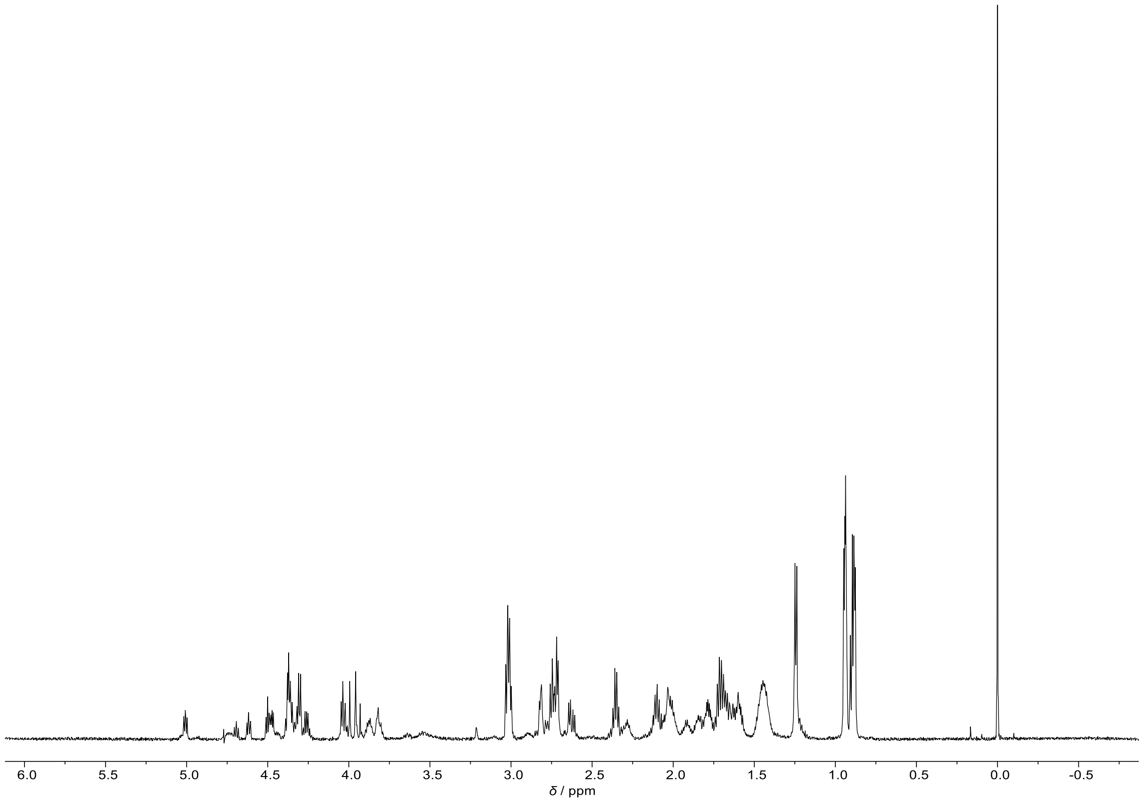

Figure S76 ^{1}H NMR spectrum of the last titration step (1:10 EF:La(III) ratio) of the La(III) to EF1-R titration series (Figure S40) after lyophilisation and redissolving in the same volume of D_2O (600 MHz, 0.003% TMSP-d_4, 30 mM MES-d_{13}, 100 mM KCl).

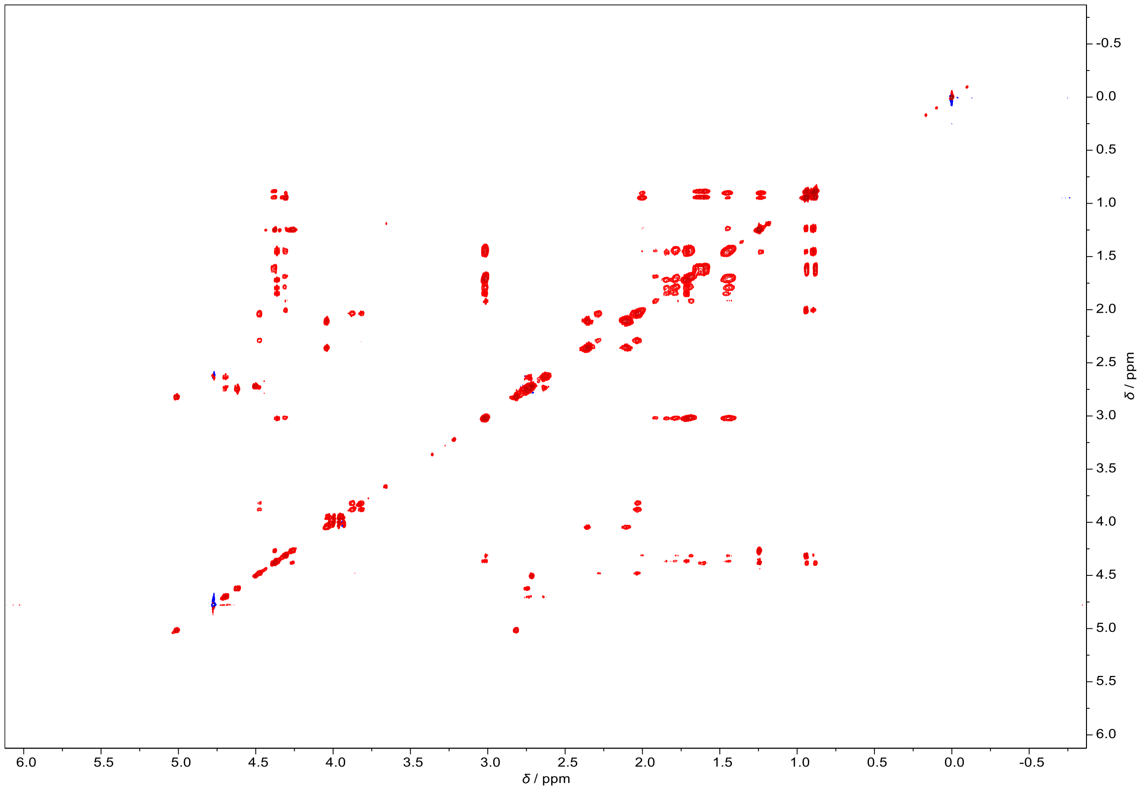

Figure S77 TOCSY spectrum of the last titration step (1:10 EF:La(III) ratio) of the La(III) to EF1-R titration series (Figure S40) after lyophilisation and redissolving in the same volume of D_2O (600 MHz, 0.003% TMSP-d_4, 30 mM MES-d_{13}, 100 mM KCl).

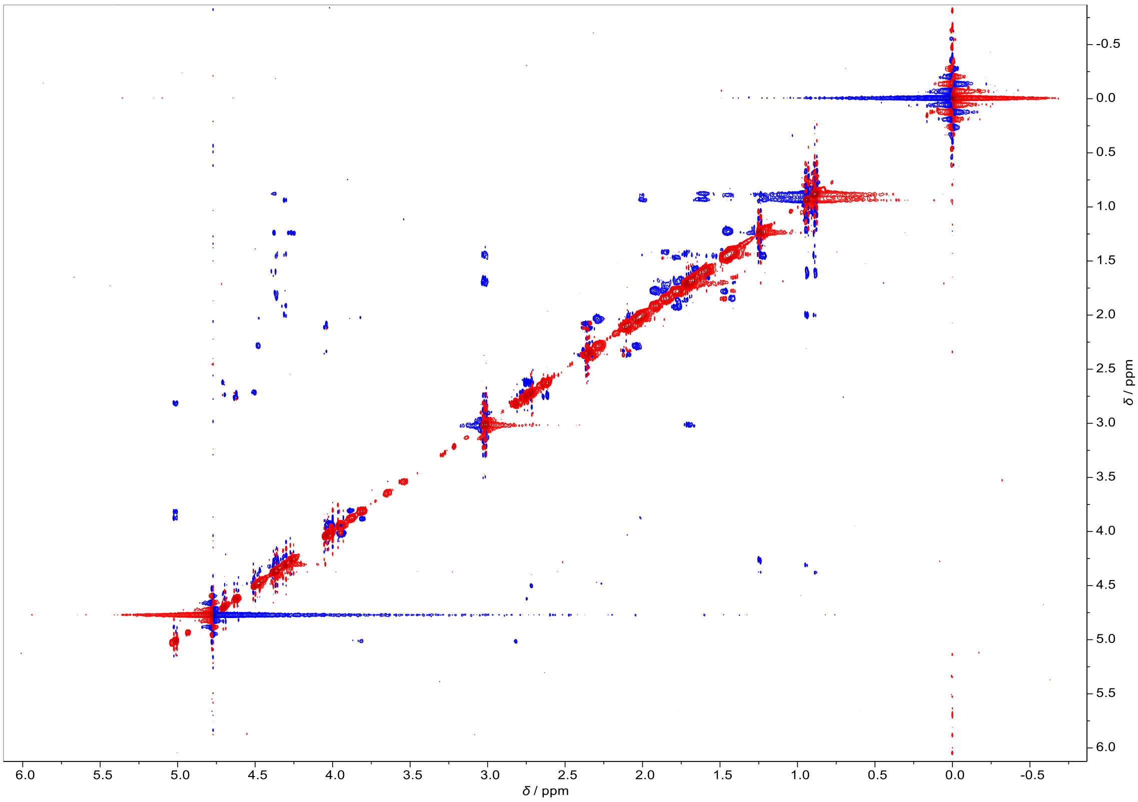

Figure S78 ROESY spectrum of the last titration step (1:10 EF:La(III) ratio) of the La(III) to EF1-R titration series (Figure S40) after lyophilisation and redissolving in the same volume of D_2O (600 MHz, 0.003% TMSP-d_4, 30 mM MES-d_{13}, 100 mM KCl).

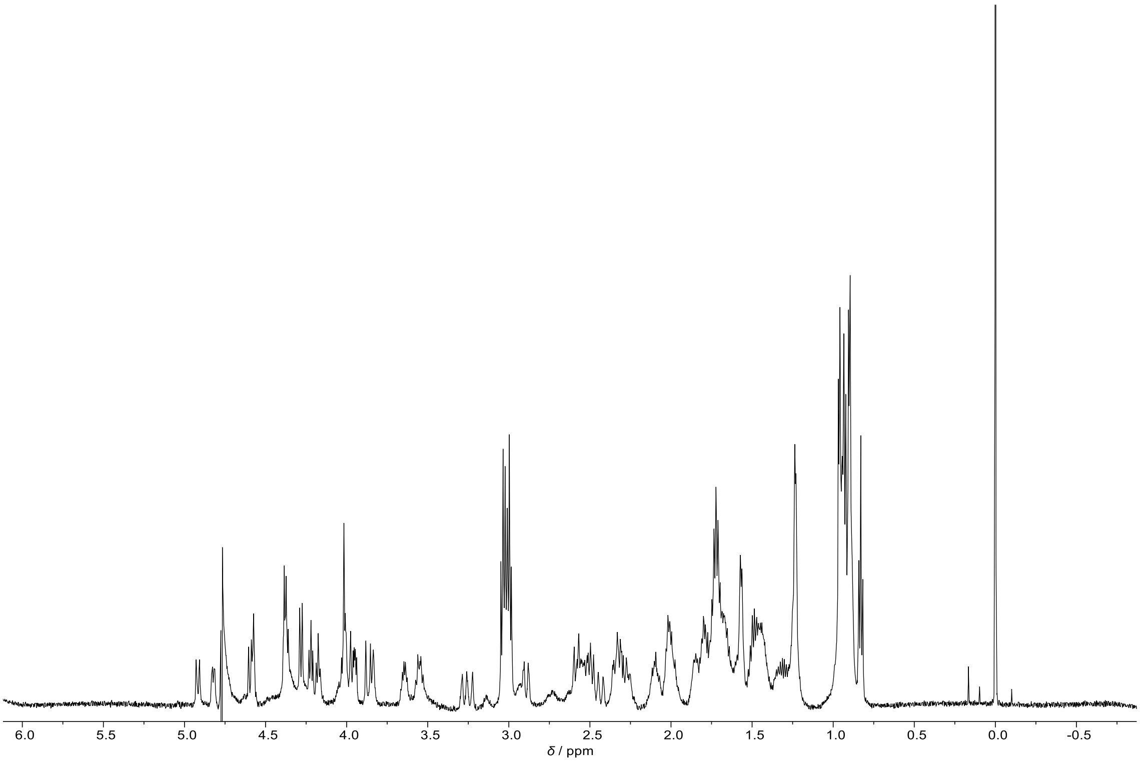

Figure S78 ^{1}H NMR spectrum of the last titration step (1:10 EF:Lu(III) ratio) of the Lu(III) to EF1-R titration series (Figure S42) after lyophilisation and redissolving in the same volume of D_2O (600 MHz, 0.003% TMSP-d_4, 30 mM MES-d_{13}, 100 mM KCl).

5.5 Supplementary MD Data

Table S15: Average coordination numbers of Eu(III) to the respective amino acid residues and to water in the reverse peptides EF1-R to EF4-R from MD trajectories (without initial restraints); bb = backbone, C-Ter. = C-terminal carboxyl group.

EF1-R	EF2-R	EF3-R	EF4-R
Glu^1 1.02	Glu^1 1.00	Glu^1 0.00	Glu^1 1.05
Asp^4 1.00	Asp^4 1.14	Asp^4 0.00	Asp^4 1.07
Thr^6 0.00	Thr^6 0.00	Thr^6 0.97	Thr^6 0.00
Asp^8 0.00	Asp^8 0.00	Asp^8 1.00/ Asp^8 0.98 (bb)	Asp^8 1.01
Lys^9 0.00	Lys^9 0.00	Asn^9 0.00	Asn^9 0.11
Asp^{10} 1.96	Asp^{10} 0.00	Asp^{10} 0.97 (bb)	Asp^{10} 1.39
Asp^{12} 1.00	Asp^{12} 1.00	Asp^{12} 1.00	-
C-Ter. 1.00	C-Ter. 1.00	C-Ter. 0.00	C-Ter. 0.00
**water 2.91**	**water 4.62**	**water 3.92**	**water 4.17**
Total 8.89	Total 8.76	Total 8.84	Total 8.8

Table S16: Average coordination numbers of La(III) and Lu(III) to the amino acid residues and to waters in the peptides EF1 and EF1-R from MD trajectories (without initial restraints); C-Ter. = C-terminal carboxyl group.

EF1-La(III)	EF1-Lu(III)	EF1-R-La(III)	EF1-R-Lu(III)
Asp^1 0.00	Asp^1 0.00	Glu^1 1.24	Glu^1 1.43
Asp^3 1.00	Asp^3 1.07	-	-
-	-	Asp^4 1.00	Asp^4 1.00
Asp^5 1.94	Asp^5 1.86	-	-
Asp^9 1.00	Asp^9 1.01	-	-
-	-	Asp^{10} 1.92	Asp^{10} 1.97
Glu^{12} 1.24	Glu^{12} 1.61	Asp^{12} 1.00	Asp^{12} 1.00
-	-	C-Ter. 1.00	C-Ter. 1.00
**water 3.62**	**water 3.11**	**water 2.77**	**water 2.47**
Total 8.8	Total 8.66	Total 8.93	Total 8.87

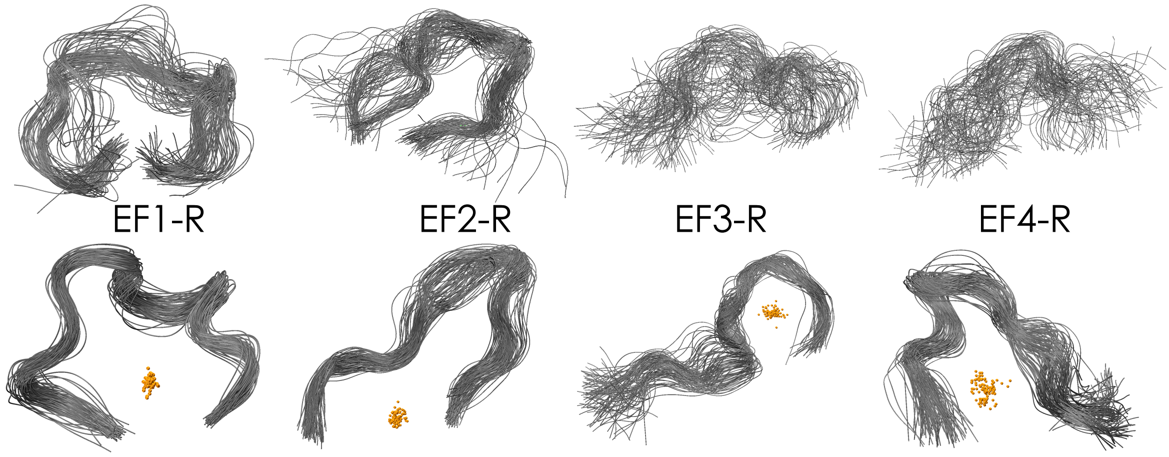

Figure S79 Superimposed MD snapshots of metal-free (upper panel) and Eu(III)-bound (lower panel, Eu in orange) reversed EF-Hand peptides (grey ribbon) for every 1 ns of the 100 ns MD trajectory (waters excluded from display), peptides are shown from N- to C-terminus (left end to right end along the ribbons).

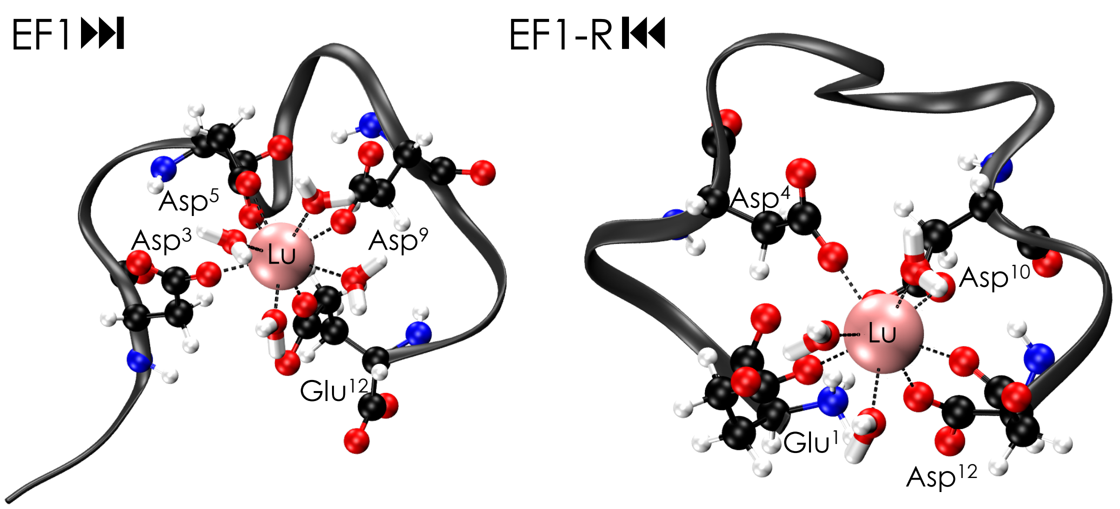

Figure S80 Representative ball-and-stick drawings of Lu(III) bound EF1 (right) and Lu(III) bound EF1-R (left). The peptide backbone is shown in grey (black: C, blue: N, red: O, white: H, pale pink: Lu) and only coordinating water molecules are depicted; peptides are shown from N- to C-terminus.

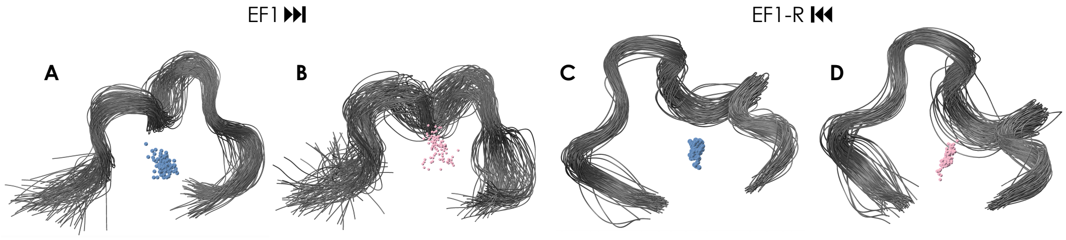

Figure S81 Superimposed MD snapshots of **(A)** La(III) bound EF1 **(B)** Lu(III) bound EF1 **(C)** La(III) bound EF1-R **(D)** Lu(III) bound EF1-R. Grey ribbons depict the peptides and La(III) is shown in light blue and Lu(III) in pale pink; peptides are shown from N- to C-terminus. Water was excluded from the superimposed snapshots for clarity.

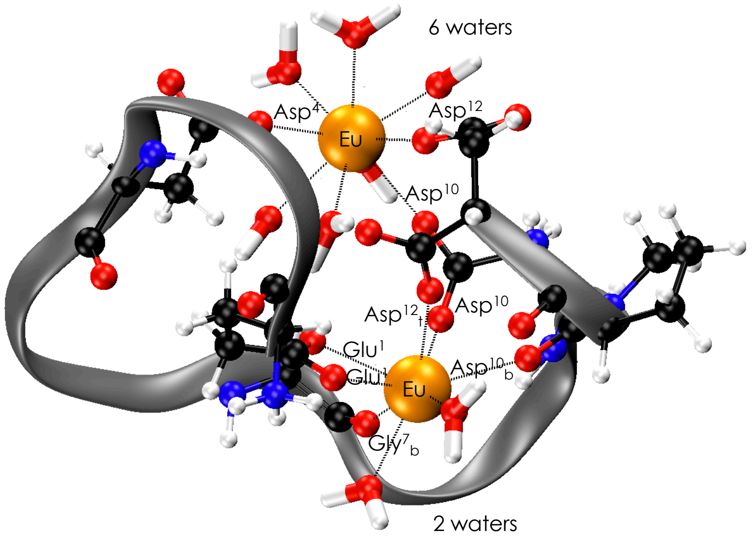

Figure S82 Representative ball-and-stick drawing of a 1:2 EF1-R to Eu(III) complex from MD trajectory. Grey ribbon depicts the peptide backbone (orange: Eu, blue: N, black: C, red: O, white: H, t = terminal and b = backbone).

6 Data Availability

Raw data files from NMR (.fid), TRLFS (.sif), ITC (.itc), and CD (.jws) experiments, along with .xyz and .pdb files of the molecular dynamics simulations are available in the RODARE repository (DOI: 10.14278/rodare.3264).

7 References

[1] S. M. Gutenthaler, S. Tsushima, R. Steudtner, M. Gailer, A. Hoffmann-Röder, B. Drobot, L. J. Daumann, *Inorg. Chem. Front.* **2022**, *9*, 4009–4021.

[2] F. Taube, B. Drobot, A. Rossberg, H. Foerstendorf, M. Acker, M. Patzschke, M. Trumm, S. Taut, T. Stumpf, *Inorg. Chem.* **2019**, *58*, 368–381.

[3] B. Drobot, M. Schmidt, Y. Mochizuki, T. Abe, K. Okuwaki, F. Brulfert, S. Falke, S. A. Samsonov, Y. Komeiji, C. Betzel, T. Stumpf, J. Raff, S. Tsushima, *Phys. Chem. Chem. Phys.* **2019**, *21*, 21213–21222.

[4] M. Leutenegger, Andor SIF image reader, *MATLAB Central File Exchange,* **2021**, https://www.mathworks.com/matlabcentral/fileexchange/11224-andor-sif-image-reader.

[5] C. A. Andersson, R. Bro, *Chemom. Intell. Lab. Syst.* **2000**, *52*, 1–4.

[6] B. Drobot, R. Steudtner, J. Raff, G. Geipel, V. Brendler, S. Tsushima, *Chem. Sci.* **2015**, *6*, 964–972.

[7] B. Drobot, A. Bauer, R. Steudtner, S. Tsushima, F. Bok, M. Patzschke, J. Raff, V. Brendler, *Anal. Chem.* **2016**, *88*, 3548–3555.

[8] D. S. Smith, Solution of Simultaneous Chemical Equilibria in Heterogeneous Systems: Implementation in Matlab, *Chemistry Faculty Publications* 14, **2019**, https://scholars.wlu.ca/chem_faculty/14.

[9] F. Menges, Spectragraphy-optical spectroscopy software (version 1.2).

[10] D. A. Case, J. T. Berryman, R.M. Betz, D.S. Cerutti, T.E. Cheatham, T.A. Darden, R.E. Duke, T.J. Giese, H. Gohlke, A.W. Goetz, N. Homeyer, S. Izadi, P. Janowski, J. Kaus, A. Kovalenko, T.S. Lee, S. LeGrand, P. Li, T. Luchko, R. Luo, B. Madej, K.M. Merz, G. Monard, P. Needham, H. Nguyen, H.T. Nguyen, I. Omelyan, A. Onufriev, D.R. Roe, A. Roitberg, R. Salomon-Ferrer, C.L. Simmerling, W. Smith, J. Swails, R.C. Walker, J. Wang, R.M. Wolf, X. Wu, D.M. York, P.A. Kollman, AMBER 2015 University of California, San Francisco **2015**.

[11] P. Li, L. F. Song, K. M. Merz, *J. Phys. Chem. B* **2015**, *119*, 883–895.
